# A Web-Based and In-Person Risk Reframing Intervention to Influence Mothers’ Tolerance for, and Parenting Practices Associated With, Children’s Outdoor Risky Play: Randomized Controlled Trial

**DOI:** 10.2196/24861

**Published:** 2021-04-27

**Authors:** Mariana Brussoni, Christina S Han, Yingyi Lin, John Jacob, Ian Pike, Anita Bundy, Guy Faulkner, Jennifer Gardy, Brian Fisher, Louise Mâsse

**Affiliations:** 1 Department of Pediatrics Faculty of Medicine University of British Columbia Vancouver, BC Canada; 2 School of Population & Public Health Faculty of Medicine University of British Columbia Vancouver, BC Canada; 3 British Columbia Injury Research & Prevention Unit British Columbia Children’s Hospital Research Institute Vancouver, BC Canada; 4 Spatial Sciences Institute University of Southern California Los Angeles, CA United States; 5 Department of Occupational Therapy Colorado State University Fort Collins, CO United States; 6 Occupational Theory Faculty of Health Sciences University of Sydney Sydney Australia; 7 School of Kinesiology Faculty of Medicine University of British Columbia Vancouver, BC Canada; 8 Bill & Melinda Gates Foundation Seattle, WA United States; 9 School of Interactive Arts & Technology Simon Fraser University Surrey, BC Canada

**Keywords:** outdoor play, mothering, independent mobility, physical activity, risk perception, risky play, risk reframing

## Abstract

**Background:**

Outdoor risky play, such as climbing, racing, and independent exploration, is an important part of childhood and is associated with various positive physical, mental, and developmental outcomes for children. Parental attitudes and fears, particularly mothers’, are a major deterrent to children’s opportunities for outdoor risky play.

**Objective:**

The aim of this study was to evaluate the efficacy of 2 versions of an intervention to reframe mothers’ perceptions of risk and change parenting behaviors: a web-based intervention or an in-person workshop, compared with the control condition.

**Methods:**

The Go Play Outside! randomized controlled trial was conducted in Canada from 2017 to 2018. Participants were recruited through social media, snowball sampling, and community notices. Mothers of children aged 6-12 years were self-assessed through eligibility questions, and those eligible and consented to participate in the study were randomized into a fully automated web-based intervention, the in-person workshop, or the control condition. The intervention was underpinned by social cognitive theory, incorporating behavior change techniques. Participants progressed through a series of self-reflection exercises and developed a goal for change. Control participants received the Position Statement on Active Outdoor Play. The primary outcome was increase in tolerance of risky play and the secondary outcome was goal attainment. Data were collected online via REDCap at baseline, 1 week, and 3 months after the intervention. Randomization was conducted using sealed envelope. Allocations were concealed to researchers at assignment and data analysis. We conducted mediation analyses to examine whether the intervention influenced elements of social cognitive theory, as hypothesized.

**Results:**

A total of 451 mothers were randomized and completed baseline sociodemographic assessments: 150 in the web-based intervention, 153 in the in-person workshop, and 148 in the control condition. Among these, a total of 351 mothers completed the intervention. At 1 week after the intervention, 113, 85, and 135 mothers completed assessments for each condition, respectively, and at 3 months after the intervention, 105, 84, and 123 completed the assessments, respectively. Compared with mothers in the control condition, mothers in the web-based intervention had significantly higher tolerance of risky play at 1 week (*P*=.004) and 3 months after the intervention (*P*=.007); and mothers in the in-person workshop had significantly higher tolerance of risky play at 1 week after the intervention (*P*=.02). No other significant outcomes were found. None of the potential mediators were found to significantly mediate the outcomes.

**Conclusions:**

The trial demonstrates that the web-based intervention was effective in increasing mothers’ tolerance for risk in play.

**Trial Registration:**

ClinicalTrials.gov NCT03374683; https://clinicaltrials.gov/ct2/show/NCT03374683

**International Registered Report Identifier (IRRID):**

RR2-10.1186/s13063-018-2552-4

## Introduction

Evidence is growing regarding the importance of outdoor play for children’s health and well-being, as are efforts to improve children’s access to these opportunities [1]. Outdoor play includes inherent risks, including those explicitly sought out by children as they explore their bodies and environments. Risky play, such as climbing trees, building dens, or even walking home from school without an adult, is an inherent and important part of outdoor play [2], and has been associated with increases in physical activity, decreases in sedentary behavior, and positive influences on physical, social, and cognitive development [3,4]. Parental attitudes and fears, particularly mothers’, are a primary barrier to children’s outdoor risky play opportunities [5-7]. They include fear of serious injury, traffic, abduction, or even the belief that time spent in outdoor play has little value in contrast to academic or other pursuits. Efforts to shift parent attitudes are a frequent focus of practitioners wanting to promote high-quality play opportunities [8], and policy makers wanting to improve rates of children walking to and from school [9,10].

Previous attempts to influence parent attitudes and practices toward risky play have been limited to in-person workshops and have not been rigorously evaluated [11]. We sought to develop and evaluate a risk reframing intervention for parents, particularly mothers as they may be typically the more limiting parent [12,13], that would be accessible through either an in-person workshop or on a web-based platform. A web-based intervention that was freely available would facilitate parents’ access, as well as the ease with which practitioners could incorporate the intervention into their existing practice by, for example, encouraging the parents in their network to complete the intervention prior to children’s enrollment in activities.

This paper reports the results of a randomized controlled trial (RCT) evaluating the efficacy of a risk reframing intervention to increase mothers’ tolerance for risky play and attain a behavior change goal related to providing risky play opportunities for their 6-12-year-old children. The intervention development, content, and theoretical framework were previously published [14]. We tested 2 versions of the intervention: a web-based and an in-person workshop. We hypothesized that participants in either intervention condition would have significantly greater increase of tolerance for risky plan than those in the control condition at 1 week and 3 months after the intervention. We also hypothesized that a greater proportion of participants in either intervention condition would attain their behavior change goal than those in the control condition.

We further examined whether social cognitive theory (SCT), the behavior change model that underpinned the development of the intervention, produced the hypothesized effect on the outcome variables. Specifically, we hypothesized that self-efficacy, outcome expectations, and knowledge of risky play would mediate the relationship between the risk reframing intervention and the outcomes: tolerance for risky play and goal attainment.

## Methods

### Study Design

A description of the protocol for this study has been published [14]. Briefly, the study was a single-blind (researchers and outcome assessors), 3-parallel condition RCT. The trial was conducted between December 2017 and September 2018 in the Metro Vancouver area of British Columbia, Canada. Measures were collected at baseline, 1 week, and 3 months after the intervention. The primary outcome was increase in tolerance of risk in play at either follow-up time point. The secondary outcome was mothers’ goal attainment at either follow-up time point.

The trial was registered with the United States National Institutes of Health’s Protocol Registration and Results System (NCT03374683) and approved by the University of British Columbia/Children’s and Women’s Health Centre of British Columbia Research Ethics Board (H15-03271). No change was made to methods after trial commencement. We followed the CONSORT-EHEALTH guidelines in reporting this study [15]. The completed checklist is presented in Multimedia Appendix 1.

### Participant Recruitment and Eligibility Criteria

Participants were recruited through advertising on online forums and social media, distributing notices through our networks, snowball sampling, and posting notices in community centers. Interested participants visited the study home page in the REDCap electronic data capture tool hosted at the British Columbia Children’s Hospital Research Institute [16]. There they were provided with a description of study procedures and information that completing the survey questions indicated consent. Potential participants were self-assessed through eligibility questions establishing that they were a mother with primary custody of a child/children aged 6-12 years; residing in the Metro Vancouver Regional District; and able to speak, read, and understand English. Computer/internet access and literacy were implicit eligibility criteria because accessing the study home page and completing the eligibility questionnaire would be otherwise impossible. Enrolled participants were emailed a unique link to the baseline questionnaire package in REDCap.

### Randomization and Blinding

Enrolled participants were automatically assigned to 1 of the 3 conditions by REDCap: control, web-based, or in-person workshop. Participants had equal likelihood of being assigned to each condition (33%, ie, nearly 170). The randomization schedule was generated beforehand via sealed envelope [17] using randomized permuted blocks of size 3, 6, and 9. The list was then transferred to REDCap. Unbeknownst to participants, randomized allocation occurred in the background before participants completed the baseline questionnaire. This had to be done because REDCap limited capabilities for real-time, streamlined randomized allocation. The nature of the intervention did not permit participant blinding, but they were informed of their allocated treatment after completing the baseline questionnaires. The in-person workshop facilitator could not be blinded to allocation as the other 2 arms did not have a facilitator. Likewise, research staff who coordinated in-person workshop schedules could not be blinded to allocation of the in-person workshop. In-person workshop participant information (name and email address) was only used to coordinate the workshop. This information was encrypted, password protected, and stored in a password-protected folder in a secured network at the British Columbia Children’s Hospital Research. However, allocations were concealed to the researchers at participant assignment and data analysis.

### Risk Reframing Intervention

Participants in the web-based intervention were provided with a link to the fully automated web-based intervention [18] which they need to complete within 1 week. A reminder was sent via email if participants did not complete the web-based intervention within 24 hours. The final reminder was sent 48 hours after their baseline entry via email. Contact information (ie, email, phone) was provided for participants to contact the research coordinator if they had any feedback or questions. Participants in the in-person workshop were scheduled to attend the in-person workshop. Briefly, the risk reframing intervention was adapted from an in-person workshop for parents and teachers developed by Bundy and colleagues [8,19] using SCT [20] to incorporate health behavior change techniques (BCTs) as per Michie et al’s taxonomy [21]. The published study protocol outlines each intervention task with the corresponding SCT construct and BCTs [14]. We sought to address common concerns about risky play and engage participants in self-reflection tasks to consider how these concepts applied to their parenting approach. The home page included a 2-minute video introduction to the topic and the tool, text defining outdoor and risky play, and outlining why they are important, as well as a brief description of the journey participants would follow in the tool. The logos of the British Columbia Children’s Hospital, the University of British Columbia, British Columbia Injury Research and Prevention Unit, and the Digital Lab were prominently displayed on the home page. As per SCT, the home page focused on building knowledge and influencing outcome expectations. BCTs included credible source, and information about health, social, and emotional consequences. Participants then proceeded through 3 chapters. Chapter 1, *Reflection*, involved considering the values and traits they most desired for their child in adulthood, their child’s favorite activities, their own favorite play activities at the same age, and what they got out of these childhood activities. As above, this chapter also focused on knowledge and outcome expectations. BCTs included framing/reframing and incompatible beliefs. Chapter 2, *What Would You Do?*, presented participants with 3 interactive video segments (climbing a tree, walking home from school, and building a den) and gave them the choice to allow or not allow the child to engage in the activity. If the participant chose to allow the activity, the child displayed excitement at the opportunity and a sense of achievement upon completion. If the participant chose to not allow the activity, the child displayed disappointment and dejection. Once the choice was made, the rest of the video played with the outcome of that choice. Participants were also asked to reflect on fears that influenced their choice, and things that helped them let go. In addition to the above SCT constructs (knowledge and outcome expectations), this chapter provided opportunities for observational learning, building self-efficacy, and identifying barriers and opportunities. BCTs included information about health, social and environmental, and emotional consequences; problem solving; demonstration of behavior; comparative imagining of future outcomes, framing/reframing; and focus on past success. Chapter 3, *Creating Your Plan*, allowed participants to review their journey and set a realistic goal as well as the timeline and steps to attain it. Participants could enter their own goal in a text box, or select from a list of suggested goals that included steps to attain it; for example, “letting my child play out in the yard without supervision” with the following steps: “Let your child play outside for a few minutes while you watch from the window. Gradually extend this time. Then try not watching out the window.” This chapter reinforced the above SCT constructs, and also encouraged building self-efficacy and outlining intentions. BCTs included goal setting (behavior and outcome), problem solving, action planning, demonstration of behavior, prompts/cues, graded tasks, comparative imagining of future outcomes, framing/reframing, and incompatible beliefs.

**Figure 1:**
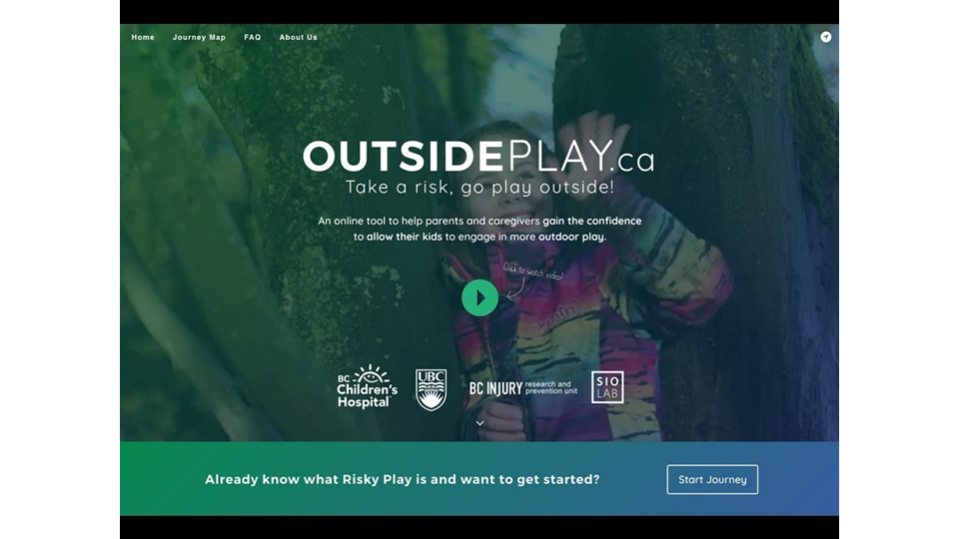
Intervention landing page.

The intervention was developed by the study authors. Once the web-based platform was complete, the in-person workshop presentation (prepared in Microsoft PowerPoint) and facilitator and participant manuals were developed using images from the web-based platform. Intervention content was frozen during the trial. The web-based intervention is available online [18]. Screenshot of the landing page can be seen in Figure 1 and the complete screenshots of the web-based intervention can be accessed in Multimedia Appendix 2. The in-person workshop materials can also be accessed in Multimedia Appendix 3.

Participants using the web-based intervention took between 15 and 45 minutes to complete it, depending on their movement through each task. The in-person workshops lasted approximately 45 minutes to 1.5 hours, depending on participant discussion. Participants in the control condition took 15-20 minutes to complete.

### Comparison Condition

Participants in the control condition were asked to review an online version of the Position Statement on Active Outdoor Play, which includes information on research and recommendations for action in addressing barriers to outdoor play [1,22].

### Outcome Measures

Measurements were taken at baseline, 1 week, and 3 months after the intervention. Participants in all conditions received an honorarium of Can $30 (US $24) at baseline and Can $15 (US $12) at each follow-up as a compensation for participation. Nonrespondents received 2 email reminders to complete survey data. Participants attending in-person workshops were provided with an additional honorarium of Can $30 (US $24) to compensate for expenses incurred in attending, such as travel or childcare.

**Figure 2:**
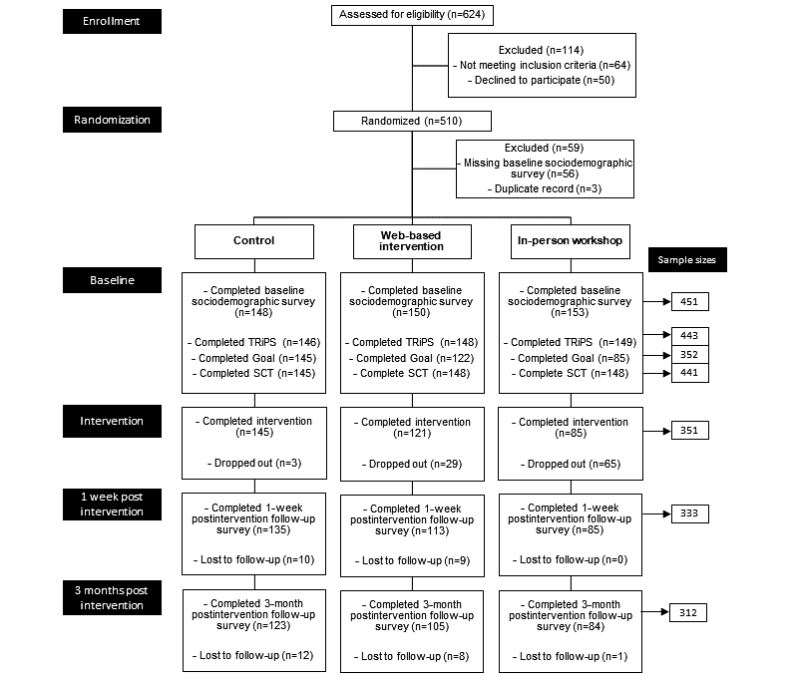
CONSORT flowchart.

The primary outcome measure was increase in the total score on the Tolerance for Risk in Play Scale (TRiPS), a 31-item measure examining adults’ tolerance of risk during children’s play, based on Sandseter’s 6-category model of risky play [23]. An earlier version of the TRiPS scale has been validated [24]. We obtained the scale from the author AB and assessed its psychometric properties in our sample using Rasch analysis. This analysis was conducted using mirt package in R software [25]. Rasch analysis of the baseline data (N=443 completed TRiPS; Figure 2) resulted in dropping 1 item (Do you allow this child to play-fight, testing who is strongest?) due to local dependence. The remaining 30 items resulted in the following model fit: root mean square error of approximation (RMSEA)=0.051 (90% CI 0.047-0.056), standardized root mean square residual (SRMSR)=0.089, Tucker–Lewis index (TLI)=0.874, comparative fit index (CFI)=0.874, and empirical reliability=0.789. Theta standardized scores from the Rasch analysis of the final 30-item TRiPS scale ranged from –3.372 to 1.975, with a mean of 0.000 (SD 0.974). A higher standardized score indicates higher tolerance of risky outdoor play.

The secondary outcome measure was self-reported behavior change, measured by participants’ self-reported progress on attaining the goal they set for themselves within the risk reframing intervention. At each follow-up, participants were reminded of their goal and asked “Did you accomplish your goal?” with “Yes” and “No” response options.

### Behavior Change Model

To assess whether the effect of the intervention was mediated by SCT as we had theorized [14], we developed measures for self-efficacy, outcome expectations, and knowledge of risky play. All measures were previously published [14]. Confirmatory factor analyses were performed on the baseline data (n=441 completed all SCT construct measures; Figure 2) to test the psychometric properties of the 3 measures, resulting in the following: (1) 4-item self-efficacy, RMSEA=0.075 (90% CI 0.020-0.139), CFI=0.987, and SRMSR=0.023; (2) 7-item outcome expectations, RMSEA=0.107 (90% CI 0.085-0.129), CFI=0.975, and SRMSR=0.023 (2 items were dropped: “promote MY CHILD’s self-confidence” and “help MY CHILD become more imaginative” due to high modification indices); and (3) 7-item knowledge of risky play: RMSEA=0.129 (90% CI 0.108-0.151), CFI=0.965, and SRMSR=0.026 (2 items were dropped: “promote A CHILD’s self-confidence” and “help A CHILD become more imaginative” due to high modification indices). Average scores of all remaining items were calculated for each SCT construct, respectively.

### Statistical Analyses

All statistical analyses were conducted in Stata 15 (StataCorp) [26].

### Power

The TRiPS is scored on a logit scale and previous research indicated that scores on the TRiPS for a sample of parents of children aged 5-13 range from 0.20 to about 1.95 with SDs in the range of 1.78-1.82 [24]. With a sample size of at least 81 mothers in each condition, a test that averaged the differences in TRiPS score from baseline to the first assessment would have 80% power at a .05 level of significance to detect a difference of 0.75 with the control condition when the SD is 1.82 and the correlation between repeated observations is 0.75.

### Descriptive Analysis

To compare sociodemographic differences between conditions, for continuous variables, one-way ANOVA was used, or Kruskal–Wallis H test (if variance was not equal between conditions). For categorical variables, chi-square test was used, or Fisher exact test if single-cell numbers were small. One-way ANOVA was used to compare TRiPS scores between different conditions at different time points. Significance level was set at *P*<.05.

### Treatment Effect of the Intervention

Linear and generalized linear mixed effects models with random intercepts and unstructured covariance were fit to analyze the effects of the intervention on TRiPS scores and goal accomplishment, respectively. In other words, the mixed effects regression analysis examined (1) whether TRiPS scores changed 1 week and 3 months after the intervention, and (2) whether these changes were greater in either experimental condition (web-based intervention or in-person workshop) compared with the control condition. Intent-to-treat analysis of TRiPS scores used last-observation-carried forward as the method of imputation, because missing data were primarily in the in-person workshop condition. Because these participants only completed baseline measures and did not receive the intervention, it is reasonable to expect their scores to remain the same throughout the study. Unstandardized (ie, raw) beta coefficients (*β*) were reported, which are interpreted as the change of TRiPS scores when comparing the experimental conditions with the control groups at baseline.

Similar to the TRiPS analyses, we conducted a generalized mixed effects regression analysis to examine the effect of the intervention on goal accomplishment, when comparing the control condition at 1 week with either of the experimental conditions at 3 months’ follow-up. Intent-to-treat analysis of goal accomplishment was not performed due to the absence of baseline data. To establish a goal, participants had to complete either intervention. As a result, there was no basis to impute values of goal accomplishment. Odds ratios (ORs) were reported, which are interpreted as the odds of attaining goals for the experimental conditions at 3 months’ follow-up, when comparing with the control conditions at 1 week after the intervention.

All models were adjusted by parents’ age, ethnicity, marital status, education, employment, housing, and household income; and children’s previous exposure to risky play, age, gender, weekday/weekend outdoor time, and chronic condition status.

### Behavior Change Model Testing

**Figure 3:**
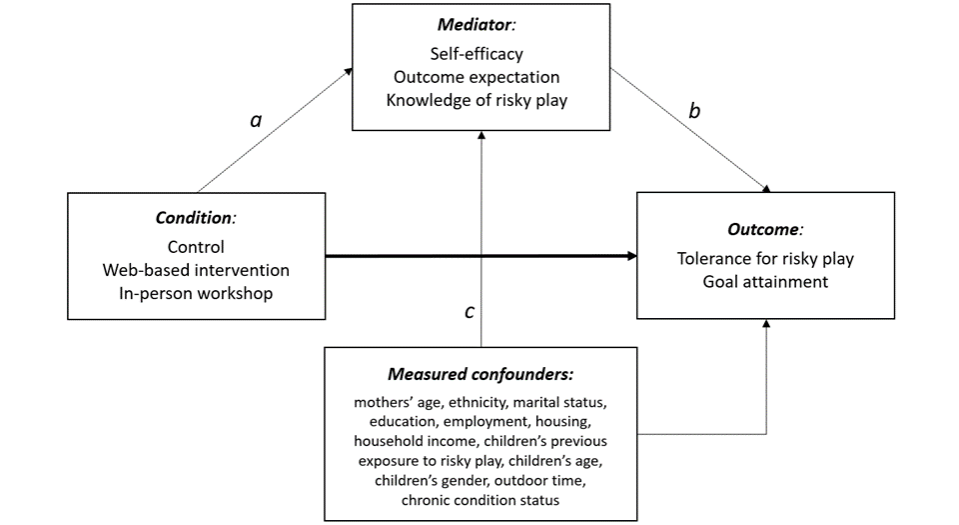
Mediation analysis.

To test whether SCT produced the hypothesized effect on the outcomes, we tested whether the effect of the intervention was mediated by self-efficacy, outcome expectations, and knowledge of risky play, as outlined in Figure 3. We followed the steps of mediation analysis for RCTs suggested by Whittle et al [27]. First, univariable linear regression models were fitted to the potential mediators to test whether there was an association between treatment and each mediator, respectively. Second, because a variable can only be a mediator of treatment, if there is a significant effect (*P*<.05) of treatment on the mediator (path *a*), a mediation analysis in the second step was only fitted to variables that were significantly associated with treatment. In the second step, the test of indirect (mediating) effect was performed by fitting regression models to the outcome, with treatment and the significant mediator found in step 1 included as covariates (path *ab*). This step of analysis controlled for baseline covariates, attempting to control for these as potential confounders in order to add robustness to our analyses. These confounders included mothers’ age, ethnicity, marital status, education, employment, housing, household income; and children’s previous exposure to risky play, children’s age and gender, weekday/weekend outdoor time, and chronic condition status. Similar to the TRiPS analyses, intent-to-treat analysis was conducted using the last-observation-carried forward.

## Results

### Overview

Figure 2 shows the study flow diagram. Recruitment began on December 1, 2017, and closed on June 18, 2018, once we had a minimum of 81 participants in each condition with complete data. A total of 510 mothers of a child between 6 and 12 years old residing in Metro Vancouver were automatically allocated to 1 of the 3 conditions by REDCap. Of these, 351 completed the intervention. While randomization produced roughly equal numbers of participants allocated to each condition, the in-person workshop condition experienced the most drop-outs (n=65). This condition involved the most time commitment for the participant and produced scheduling challenges. As such, despite the additional Can $30 (US $24) incentive to participate, it was most difficult to maintain participants in this condition. However, of the mothers attending the workshop, only 1 was lost to follow-up. We were able to ensure fidelity to the web-based intervention as we received a summary of all responses when a participant completed the intervention. These were reviewed to ensure full completion. Fidelity to the in-person workshop was established through attendance to the workshop.

### Participant Characteristics

Baseline sociodemographic data from 451 mothers were included in our analyses. Participant demographics are displayed in Table 1. There were no statistically significant differences between conditions with respect to demographic characteristics at baseline (see the “*P* value column” in Table 1). There were statistically significant differences between conditions for child’s disability/chronic condition at both 1 week (*P*=.03) and 3 months (*P*=.03) after the intervention, with the percentage of children having chronic conditions in the web-based intervention higher than that in the other 2 conditions (10/113 [8.8%] for 1 week after the intervention, and 9/105 [8.6%] for 3 months after the intervention, respectively). We also further compared the sociodemographic characteristics between those who completed the intervention (N=351) and those who did not complete the intervention (N=100), and found that employed mothers were more likely to complete the intervention (75.8% [266/351] versus 65.0% [65/100], respectively, *P*=.03). There were no statistical differences for other sociodemographic characteristics (see Multimedia Appendix 4).

**Table 1:** Participant demographics at baseline, after randomization.

Demographics		Control	Web-based intervention	In-person workshop	*P* value
Participants who completed the baseline sociodemographic assessment (N=451), n		148	150	153	.09
Age (N=450)^a^, mean (SD)		40.7 (5.3)	40.8 (5.5)	39.6 (5.0)	
**Ethnicity (N=451), n (%)**					.24
	White	101 (68.2)	112 (74.7)	117 (76.5)	
	Others	47 (31.8)	38 (25.3)	36 (23.5)	
**Marital status (N=451), n (%)**					.39
	Married/Common-law	118 (79.7)	125 (83.3)	131 (85.6)	
	Others	30 (20.3)	25 (16.7)	22 (14.4)	
**Education (N=447)^b^, n (%)**					.32
	Less than university/college	36 (24.3)	36 (24.3)	33 (21.9)	
	University/college	66 (44.6)	80 (54.1)	72 (47.7)	
	More than university/college	46 (31.1)	32 (21.6)	46 (30.5)	
**Employment (N=451), n (%)**					.35
	Employed for wages/self-employed	115 (77.7)	107 (71.3)	44 (28.8)	
	Unemployed	33 (22.3)	43 (28.7)	109 (71.2)	
**Home dwelling (N=451), n (%)**					.66
	Single detached	69 (46.6)	77 (51.3)	72 (47.1)	
	Others	79 (53.4)	73 (48.7)	81 (52.9)	
**Income (N=451), n (%)^c^**					.80
	<Can $63,300	36 (24.3)	40 (26.7)	33 (21.6)	
	Can $63,300-Can $103,299	49 (33.1)	40 (26.7)	50 (32.7)	
	≥Can $103,300	52 (35.1)	54 (36.0)	55 (35.9)	
	Prefer not to answer	11 (7.4)	16 (10.7)	15 (9.8)	
**Exposure to risky play information (N=451), n (%)**					.15
	No	22 (14.9)	28 (18.7)	36 (23.5)	
	Yes	126 (85.1)	122 (81.3)	117 (76.7)	
Child age (N=451), mean (SD)		8.4 (1.7)	8.1 (1.7)	8.0 (1.9)	.10
**Child sex (N=447), n (%)^d^**					.78
	Boy	85 (58.6)	82 (54.7)	85 (55.9)	
	Girl	60 (41.4)	68 (45.3)	67 (44.1)	
**Child’s disability/chronic condition (N=451)^e^, n (%)**					.30
	No	143 (96.6)	140 (93.3)	148 (96.7)	
	Yes	5 (3.4)	10 (6.7)	5 (3.3)	
**Outdoor time (hours) (N=451)^f^, mean (SD)**					
	Weekday	3.0 (3.6)	2.8 (3.2)	2.6 (2.8)	.90
	Weekend	2.7 (2.2)	2.8 (2.0)	2.9 (2.8)	.65

^a^One parent reported age=7 years, which is not reasonable, so we treated that data as missing.

^b^Prefer not to answer, n=4 (n=2 for web-based intervention; n=2 for in-person workshop).

^c^Can $ 1=US $0.80.

^d^A total of four children did not have information regarding their sex (n=3 in the control group, n=1 in the in-person workshop).

^e^Fisher exact test due to small sample size in single cell.

^f^Kruskal–Wallis H test due to non-equal variance between conditions.

### Primary Outcome: TRiPS

Table 2 presents the description of TRiPS scores by treatment conditions and time point, without accounting for treatment effects nor adjusting for sociodemographic characteristics. There were no statistical differences in TRiPS scores between different conditions at each time point. Table 3 describes findings of the mixed effects regression analysis. Mothers who completed the web-based intervention condition had significantly higher TRiPS scores than mothers who completed the control condition at 1 week (=.26, 95% CI 0.09-0.43; *P*=.003) and 3 months (=.24, 95% CI 0.06-0.42; *P*=.01) after the intervention, indicating sustained change. Mothers who completed the in-person workshop condition had significantly higher increases in TRiPS scores than those in the control condition at 1 week after the intervention (=.19, 95% CI 0.06-0.38; *P*=.04). No statistically significant differences were found when comparing mothers who completed the in-person workshop condition with those in the control condition at 3 months after the intervention (=.09, 95% CI –0.10 to 0.29; *P*=.33).

Results of the intention-to-treat analyses for the effects of the intervention on TRiPS score largely replicated the analyses described above, indicating that mothers in the web-based intervention condition were significantly more likely to increase their TRiPS scores at 1 week (=.25, 95% CI 0.08-0.42; *P*=.004) and 3 months (=.24, 95% CI 0.06-0.42; *P*=.007) after the intervention compared with those in the control condition. Mothers in the in-person workshop condition had significantly higher increases in TRiPS scores than those in the control condition at 1 week after the intervention (=.22, 95% CI 0.03-0.40; *P*=.02). No statistically significant differences were found when comparing mothers in the in-person workshop with those in the control condition at 3 months after the intervention (=.13, 95% CI –0.06 to 0.32; *P*=.17).

**Table 2:** Description of TRiPS^a^ scores by treatment conditions and time points.

Evaluation period	Sample size, n	Control, mean (SD)	Web-based intervention, mean (SD)	In-person workshop, mean (SD)	*P* value for 1-way ANOVA
Baseline	443	0.05 (1.03)	–0.11 (0.94)	0.06 (0.94)	.24
Completed intervention	351	0.05 (1.03)	–0.14 (0.97)	0.18 (0.87)	.05
1 week after the intervention	333	–0.09 (1.03)	–0.06 (1.05)	0.22 (0.80)	.06
3 months after the intervention	312	–0.03 (0.92)	–0.09 (0.93)	0.15 (0.80)	.19

^a^TRiPS: Tolerance for Risk in Play Scale.

**Table 3:** Results of mixed effects regression analysis for change in TRiPS^a^ scores by treatment condition and time point.

Regression and group comparisons				Coefficients (95% CI)		*P* value for coefficients		*P* value for joint test of main effects
**Raw TRiPS theta scores (N=351, for those who completed the intervention)^b^**								
	**Treatment effects**						.21	
		Web based versus control	–0.14 (–0.36 to 0.08)		.21			
		In-person versus control	0.09 (–0.15 to 0.34)		.46			
	**Time effects**						.86	
		1 week versus baseline	–0.16 (–0.27 to –0.04)		.007			
		3 months versus baseline	–0.13 (–0.25 to –0.01)		.03			
	**Treatment by time effects**						.02	
		Web based versus control by 1 week versus baseline	0.26 (0.09 to 0.43)		.003			
		Web based versus control by 3 months versus baseline	0.24 (0.06 to 0.42)		.01			
		In-person versus control by 1 week versus baseline	0.19 (0.06 to 0.38)		.04			
		In-person versus control by 3 months versus baseline	0.10 (0.00 to 0.29)		.33			
**Intention-to-treat analysis (imputed TRiPS theta scores) (N=443, for those who were randomized to a condition and completed baseline sociodemographic and TRiPS survey)^c^**								
	**Treatment effects**						.47	
		Web based versus control	–0.13 (–0.34 to 0.07)		.20			
		In-person versus control	0.00 (–0.21 to 0.21)		.98			
	**Time effects**						.90	
		1 week versus baseline	–0.16 (–0.27 to –0.04)		.008			
		3 months versus baseline	–0.14 (–0.26 to –0.02)		.02			
	**Treatment by time effects**						.01	
		Web based versus control by 1 week versus baseline	0.25 (0.08 to 0.42)		.004			
		Web based versus control by 3 months versus baseline	0.24 (0.06 to 0.42)		.007			
		In-person versus control by 1 week versus baseline	0.22 (0.03 to 0.40)		.02			
		In-person versus control by 3 months versus baseline	0.13 (–0.06 to 0.32)		.17			

^a^TRiPS: Tolerance for Risk in Play Scale.

^b^Significant (*P*<.10) sociodemographic predictors include ethnicity, housing condition, previous exposure to risky play information, child’s age, child’s disability/chronic conditions, and weekend outdoor time.

^c^Significant (*P*<.10) sociodemographic predictors include ethnicity, parental employment status, housing condition, previous exposure to risky outdoor play information, child’s age, child’s disability/chronic conditions, and weekend outdoor time.

### Secondary Outcome: Goal Attainment

Table 4 presents the results of the generalized mixed effects regression analysis. There was no statistical difference in goal attainment between mothers in the web-based intervention and the control condition at 3 months after the intervention as compared with 1 week after the intervention (OR 0.59, 95% CI 0.18-1.91; *P*=.37). There was also no statistical difference in goal attainment between mothers in the in-person workshop and the control condition at 3 months after the intervention as compared with 1 week after the intervention (OR 0.55, 95% CI 0.17-1.76; *P*=.31).

**Table 4:** Results of the mixed effects regression analysis for goal attainment by treatment condition and time.^a^

Regression and group comparisons			Odds ratios (95% CI)		*P* value for coefficients		*P* value for joint test of main effects
**Treatment effects**							.004
	Web based versus control	3.36 (1.32-8.54)		.01			
	In-person versus control	0.72 (0.28-1.85)		.49			
**Time effects**							<.001
	3 months versus 1 week	7.27 (3.11-17.02)		<.001			
**Treatment by time effects**							.53
	Web based versus control by 3 months versus 1 week	0.59 (0.18-1.91)		.37			
	In-person versus control by 3 months versus 1 week	0.55 (0.17-1.76)		.31			

^a^Significant (*P*<.05) demographic predictors include ethnicity, child’s disability/chronic conditions, and weekend outdoor time.

### Behavior Change Model Mediation Analysis

Because only significant intervention effects were observed for TRiPS score and not goal attainment, mediation analyses were only conducted for TRiPS data. With regard to the direct effects of treatment conditions on the 3 SCT constructs, none of the 3 SCT constructs (self-efficacy, *P*=.16; outcome expectations, *P*=.24; and knowledge of risky play, *P*=.12) was associated with treatment exposures in the unadjusted models, neither for those who completed the intervention (N=351) nor for the intention-to-treat analysis sample (N=441, for those who completed baseline sociodemographic, TRiPS, and SCT survey; all *P* values were >.05). As there was no direct association between treatment and the SCT constructs (path *a*), further mediation analyses were not conducted because it was not possible for the SCT constructs to have an impact on the study outcome via mediation (ie, path *ab*). Therefore, none of the potential mediators were found to significantly mediate outcome.

## Discussion

### Principal Findings

Our hypotheses were partially supported. Mothers receiving both the web-based intervention and the in-person workshop intervention at 1 week after the intervention reported significantly higher increases in their tolerance for risky play than mothers in the control condition at baseline. These differences remained significant at 3 months after the intervention for mothers receiving the web-based intervention but not mothers receiving the in-person workshop intervention. There were no significant differences in goal attainment.

We did not have sufficient statistical power to test the difference in efficacy between the 2 versions of the intervention. In any case, the significant results for the web-based intervention and not for the in-person workshop at 3 months after the intervention are unexpected, as the in-person workshops provided arguably a higher intervention dose given the greater length required in participation and the more social aspect of the experience that could influence participant perceptions of social support, a construct of SCT [20]. Workshop participation required fitting into a set schedule and demanded significantly more time commitment and effort than the other conditions. Thus, it required a higher motivation level and had greater attrition. Those who ended up attending the in-person workshop might have already been fully aware of the benefits of children’s outdoor play, which inadvertently left limited room for improvement. However, this possibility is not supported by baseline scores on the TRiPS, which did not differ between conditions. Another possible explanation could result from the fact that the number of participants attending each workshop varied considerably (2-12 participants), which could negatively impact the extent of discussion [28]. Further, workshops were facilitated by a professional facilitator, without content expertise in outdoor play and who was not a parent. SCT stresses the importance of relatable peers modeling behavior to encourage behavior change [28]. Thus, appropriate probing to foster discussion and participants’ engagement with the topic may have been hampered.

It is not readily apparent why there was a null finding for goal attainment. It is possible that the binary yes/no outcome may have hampered participant responses in that participants may not have indicated they had attained their goal unless they perceived that all aspects were complete. In addition, goals may have been overly ambitious and did not have clear steps to attain them, such as “give my child more independence to enjoy freedom.” Future iterations of the intervention should encourage users to set a more realistic and actionable goal. Sample actionable goals were provided in the tool but it is possible that these required further details on actions.

In sum, our findings indicate the efficacy of both versions of the risk reframing intervention for changing mothers’ tolerance to risky play, with the web-based intervention displaying long-term effects.

### Behavior Change Model

The hypothesized relationship between the constructs of SCT (self-efficacy, outcome expectation, and knowledge) and the intervention outcomes was not supported. We offer the following explanations for these findings. First, the intervention may have been of insufficient intensity or duration to influence these constructs. To reduce access and engagement barriers, we sought to develop an intervention that was efficient, would require limited time commitment, and would not necessitate repeat visits [14]. Therefore, future iterations may attempt to increase the intervention dose to determine whether this would impact the mediators as we hypothesized. Second, it is possible that our SCT constructs were not sensitive enough to detect change over time. While we evaluated the psychometric properties of our measures, we do not know whether these measures are sensitive to change as they were developed for this study. Third, although we hypothesized that both self-efficacy and outcomes expectations would increase as a result of the intervention, it is possible that going through the intervention and attempting to change parenting practices proved more difficult than anticipated for some parents and might explain why no change was observed for the SCT constructs. We did not collect data on other potential factors that could influence parents’ success, such as self-efficacy in overcoming barriers, thus limiting our insight on these results.

### Strengths and Limitations

Our research is the first to use health behavior change theory and BCTs to develop an intervention to reframe mothers’ perceptions and influence their parenting behaviors related to outdoor risky play. As recommended by published guidelines [29,30], we are also among the first to test the active ingredients of the intervention, examining the hypothesized relationships between SCT constructs and the outcomes. In addition, our use of RCT methods represents a gold-standard methodological evaluation technique. Previous research reported on but did not evaluate an in-person workshop intervention separately from a loose parts intervention in the playground [11]. Furthermore, our RCT evaluated 2 versions of the risk reframing intervention, providing insight into the efficacy of alternative delivery methods with important implications for practice.

There were several limitations to the study. Given the nature of the intervention, it was not possible to blind participants to their allocation, thus potentially introducing sources of bias. Furthermore, the intervention was developed based on research conducted in Western settings and was only available in English. As such, it may not reflect the perspectives and needs of other cultural and linguistic conditions and non-Western settings. Likewise, because one of our conditions necessitated in-person participation, we were only able to recruit participants within a limited geographic area, thus potentially limiting the applicability of our findings to other areas, such as rural communities.

### Conclusions

Our findings provide confidence in encouraging use and broad dissemination of the web-based intervention. Given the ease of distribution, no cost to users, and low resource requirement for ongoing maintenance of the web-based tool, it is encouraging that this was an effective model and can provide the basis for further iterations and versions. Future research is necessary to examine the risk perceptions of parents from other cultural conditions and settings to facilitate the development of interventions that are applicable to their settings and realities.

## Appendix

Multimedia Appendix 1 CONSORT E-HEALTH checklist.

Multimedia Appendix 2 Screenshots of the web-based risk reframing intervention at OutsidePlay.ca.

Multimedia Appendix 3 Risk reframing intervention in-person workshop.

Multimedia Appendix 4 Nonstatistical differences for other sociodemographic characteristics.

## Abbreviations

BCT: behavior change technique
CFI: comparative fit index
OR: odds ratios
RCT: randomized controlled trial
RMSEA: root mean square error of approximation
SCT: social cognitive theory
SRMSR: standardized root mean square residual
TLI: Tucker–Lewis index
TRiPS: Tolerance for Risk in Play Scale

## References

[1] Tremblay MS, Gray C, Babcock S, Barnes J, Bradstreet CC, Carr D, Chabot G, Choquette L, Chorney D, Collyer C, Herrington S, Janson K, Janssen I, Larouche R, Pickett W, Power M, Sandseter EBH, Simon B, Brussoni M (2015). Position Statement on Active Outdoor Play. Int J Environ Res Public Health.

[2] Hansen Sandseter EB (2007). Categorising risky play—how can we identify risk‐taking in children's play?. European Early Childhood Education Research Journal.

[3] Gray C, Gibbons R, Larouche R, Sandseter E, Bienenstock A, Brussoni M, Chabot G, Herrington S, Janssen I, Pickett W, Power M, Stanger N, Sampson M, Tremblay M (2015). What Is the Relationship between Outdoor Time and Physical Activity, Sedentary Behaviour, and Physical Fitness in Children? A Systematic Review. Int J Environ Res Public Health.

[4] Brussoni M, Gibbons R, Gray C, Ishikawa T, Sandseter EBH, Bienenstock A, Chabot G, Fuselli P, Herrington S, Janssen I, Pickett W, Power M, Stanger N, Sampson M, Tremblay MS (2015). What is the Relationship between Risky Outdoor Play and Health in Children? A Systematic Review. Int J Environ Res Public Health.

[5] Lee H, Tamminen KA, Clark AM, Slater L, Spence JC, Holt NL (2015). A meta-study of qualitative research examining determinants of children's independent active free play. Int J Behav Nutr Phys Act.

[6] Jelleyman C, McPhee J, Brussoni M, Bundy A, Duncan S (2019). A Cross-Sectional Description of Parental Perceptions and Practices Related to Risky Play and Independent Mobility in Children: The New Zealand State of Play Survey. Int J Environ Res Public Health.

[7] Boxberger K, Reimers A (2019). Parental Correlates of Outdoor Play in Boys and Girls Aged 0 to 12-A Systematic Review. Int J Environ Res Public Health.

[8] Niehues A, Bundy A, Broom A, Tranter P (2016). Reframing healthy risk taking: Parents’ dilemmas and strategies to promote children’s well-being. Journal of Occupational Science.

[9] McDonald N, Aalborg A (2009). Why Parents Drive Children to School: Implications for Safe Routes to School Programs. Journal of the American Planning Association.

[10] Council of Chief Medical Officers of Health (2018). Active Outdoor Play Statement from the Council of Chief Medical Officers of Health.

[11] Niehues A, Bundy A, Broom A, Tranter P, Ragen J, Engelen L (2013). Everyday uncertainties: reframing perceptions of risk in outdoor free play. Journal of Adventure Education & Outdoor Learning.

[12] Schoeppe S, Duncan MJ, Badland HM, Alley S, Williams S, Rebar AL, Vandelanotte C (2015). Socio-demographic factors and neighbourhood social cohesion influence adults' willingness to grant children greater independent mobility: A cross-sectional study. BMC Public Health.

[13] Brussoni M, Olsen LL, Creighton G, Oliffe JL (2013). Heterosexual gender relations in and around childhood risk and safety. Qual Health Res.

[14] Brussoni M, Ishikawa T, Han C, Pike I, Bundy A, Faulkner G, Mâsse LC (2018). Go Play Outside! Effects of a risk-reframing tool on mothers' tolerance for, and parenting practices associated with, children's risky play: study protocol for a randomized controlled trial. Trials.

[15] Eysenbach G (2011). CONSORT-EHEALTH: improving and standardizing evaluation reports of Web-based and mobile health interventions. J Med Internet Res.

[16] Harris PA, Taylor R, Thielke R, Payne J, Gonzalez N, Conde JG (2009). Research electronic data capture (REDCap)--a metadata-driven methodology and workflow process for providing translational research informatics support. J Biomed Inform.

[17] sealed envelope.

[18] Brussoni M, Han C, Jacob J (2017). OutsidePlay.ca: Take a risk, go play outside!.

[19] Bundy AC, Naughton G, Tranter P, Wyver S, Baur L, Schiller W, Bauman A, Engelen L, Ragen J, Luckett T, Niehues A, Stewart G, Jessup G, Brentnall J (2011). The Sydney playground project: popping the bubblewrap--unleashing the power of play: a cluster randomized controlled trial of a primary school playground-based intervention aiming to increase children's physical activity and social skills. BMC Public Health.

[20] Bandura A (2001). Social cognitive theory: an agentic perspective. Annu Rev Psychol.

[21] Michie S, Richardson M, Johnston M, Abraham C, Francis J, Hardeman W, Eccles MP, Cane J, Wood CE (2013). The behavior change technique taxonomy (v1) of 93 hierarchically clustered techniques: building an international consensus for the reporting of behavior change interventions. Ann Behav Med.

[22] ParticipACTION (2015). The Biggest Risk is Keeping Kids Indoors. The 2015 ParticipACTION Report Card on Physical Activity for Children and Youth.

[23] Sandseter EBH (2009). Characteristics of risky play. Journal of Adventure Education & Outdoor Learning.

[24] Hill A, Bundy AC (2014). Reliability and validity of a new instrument to measure tolerance of everyday risk for children. Child Care Health Dev.

[25] Chalmers RP (2012). : A Multidimensional Item Response Theory Package for the Environment. J. Stat. Soft.

[26] StataCorp (2017). Stata Statistical Software: Release 15 Notes.

[27] Whittle R, Mansell G, Jellema P, van der Windt D (2017). Applying causal mediation methods to clinical trial data: What can we learn about why our interventions (don't) work?. Eur J Pain.

[28] Bartholomew LK, Markham C, Ruiter RAC, Fernandez ME, Kok G, Parcel GS (2016). Planning Health Promotion Programs: An Intervention Mapping Approach (4th ed.).

[29] Craig P, Dieppe P, Macintyre S, Michie S, Nazareth I, Petticrew M, Medical RCG (2008). Developing and evaluating complex interventions: the new Medical Research Council guidance. BMJ.

[30] Gottfredson DC, Cook TD, Gardner FEM, Gorman-Smith D, Howe GW, Sandler IN, Zafft KM (2015). Standards of Evidence for Efficacy, Effectiveness, and Scale-up Research in Prevention Science: Next Generation. Prev Sci.

