# Supplementary material for: A Web-Based and In-Person Risk Reframing Intervention to Influence Mothers’ Tolerance for, and Parenting Practices Associated With, Children’s Outdoor Risky Play: Randomized Controlled Trial
Source: J Med Internet Res. 2021 Apr 27;23(4):e24861. doi: 10.2196/24861 (PMC8114163; doi:10.2196/24861)
Supplement: Multimedia Appendix 2 [file jmir_v23i4e24861_app2.pdf]

OUTSIDEPLAY.ca

Take a risk, go play outside!

An online tool to help parents and caregivers **gain the confidence** to **allow their kids** to engage in more **outdoor play.**

Click to watch video!

BC Children's Hospital

UBC

BC INJURY research and prevention unit

digital LAB

Chapter 1

REFLECTION

Think about a child you care about (between the age of 6 - 12 if possible). This is the child you'll be thinking about during your journey, and could be your son or daughter, a student, or another child close to you.

Child's First Name/Nickname

Child's Age

Child's Gender

I am the child's...

Begin Journey

Skip

VALUES & TRAITS

The 3 most important attributes I want **my child** to have when she grows up are:

Assertive	Compassionate	Confident
Courageous	Curious	Independent
Joyful	Kind	Learns from Mistakes
Makes good decisions	Passionate	Powerful
Recognition	Resilient	Sense of Belonging
Self-Content	Socially Just	Spirituality
Imaginative	Wealth	Wisdom

MY CHILD'S ACTIVITIES

What are some of my child's' favourite things to do on their own or with friends?

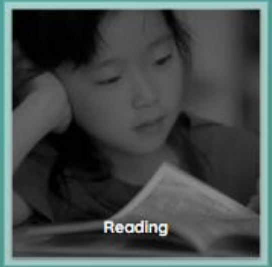

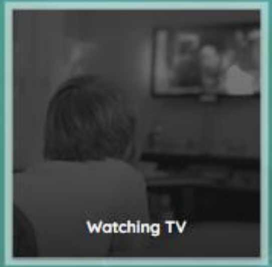

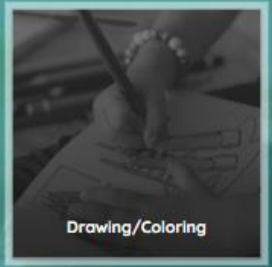

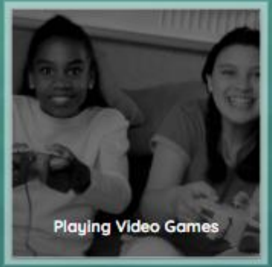

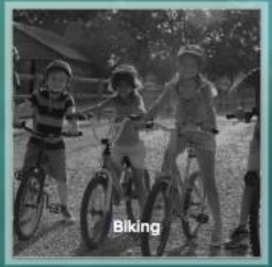

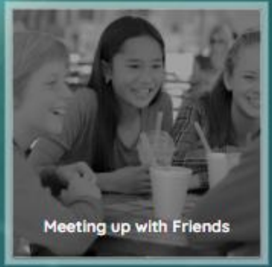

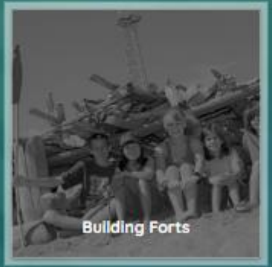

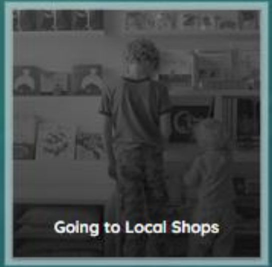

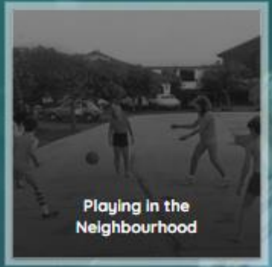

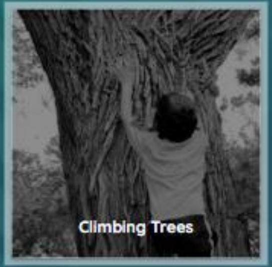

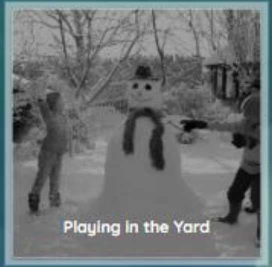

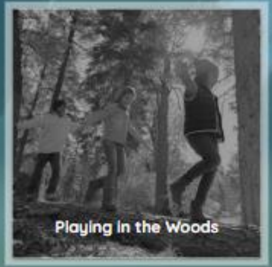

THINK BACK TO YOUR OWN CHILDHOOD PLAY

What were some of your favourite things to do on your own or with friends growing up? Where were you? Who was typically with you? What were you doing?

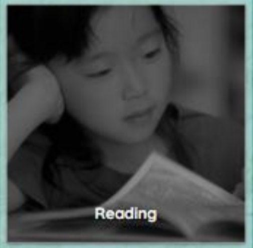

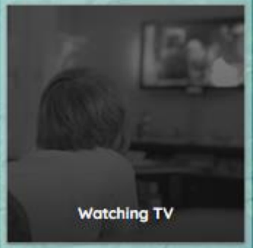

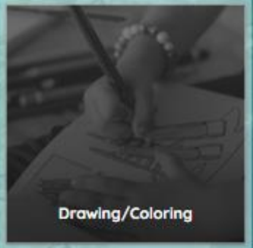

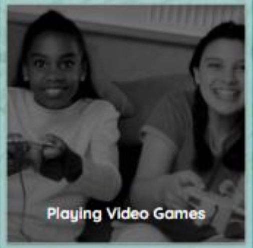

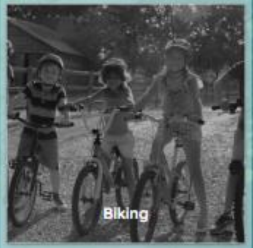

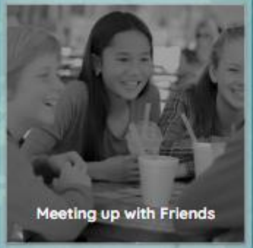

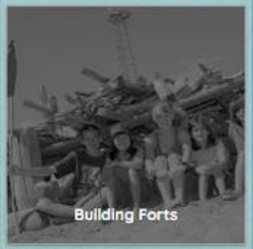

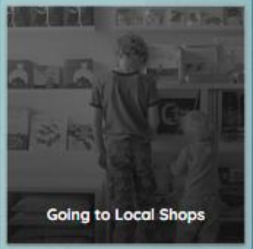

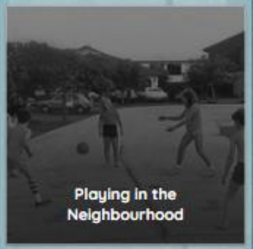

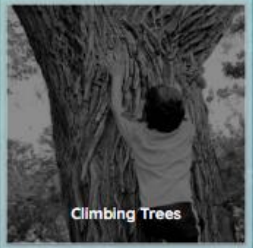

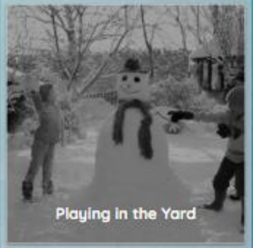

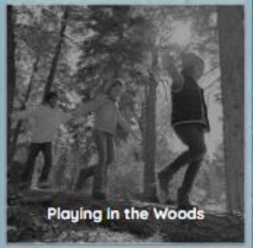

WHAT DID YOU GET OUT OF IT?

Think back to what you learned from these experiences and how they influenced you. What did you get out of these play activities?

Assertive	Compassionate	Confident
Courageous	Curious	Independent
Joyful	Kind	Learns from Mistakes
Makes good decisions	Passionate	Powerful
Recognition	Resilient	Sense of Belonging
Self-Content	Socially Just	Spirituality
Imaginative	Wealth	Wisdom

Continue

SAME OR DIFFERENT?

How do my child's activities compare to what you remember doing at that age?

MY CHILDHOOD ACTIVITIES

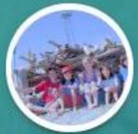

ACTIVITIES WE SHARE

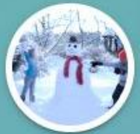

MY CHILD'S ACTIVITIES

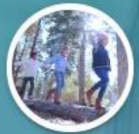

WHAT I GOT OUT OF IT

Compassionate
Joyful

WHAT I WANT FOR MY CHILD

Courageous
Resilient
Imaginative

Next

Chapter 2

WHAT WOULD YOU DO?

Imagine yourself as the parent in each interactive video segment. Think about what you would do in these situations. Your choices will impact what happens in the story and the outcomes. Reflect on the factors that influenced your decisions and discover what you could have done differently.

Next

CLIMBING A TREE

Imagine you and your daughter are going for an afternoon walk around a park. You decided to take a break and sit on a nearby bench while your daughter begins to play around some trees.

Begin Scenario

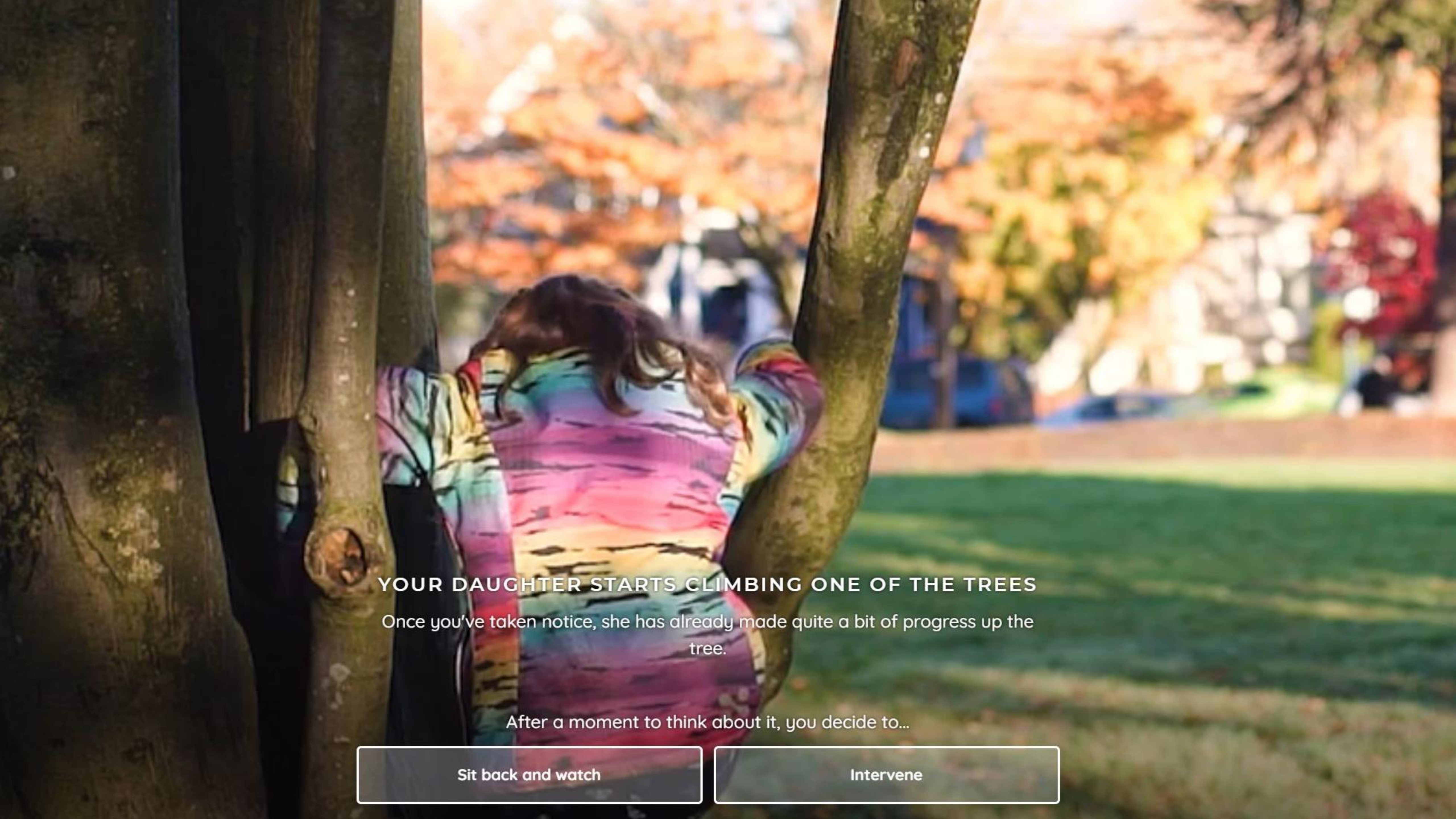

YOUR DAUGHTER STARTS CLIMBING ONE OF THE TREES

Once you've taken notice, she has already made quite a bit of progress up the tree.

After a moment to think about it, you decide to...

Sit back and watch

Intervene

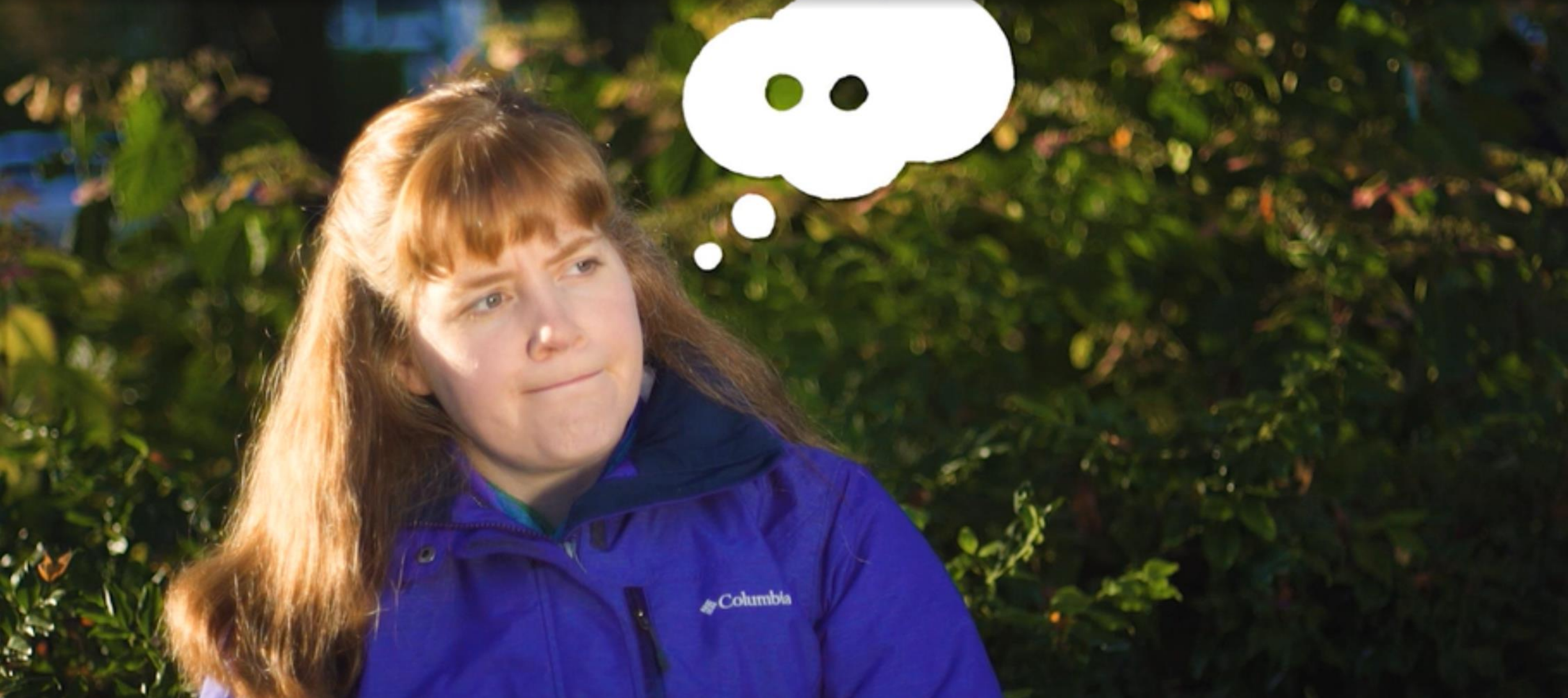

YOUR DAUGHTER STARTS CLIMBING ONE OF THE TREES

Once you've taken notice, she has already made quite a bit of progress up the tree.

After a moment to think about it, you decide to...

Sit back and watch | Intervene

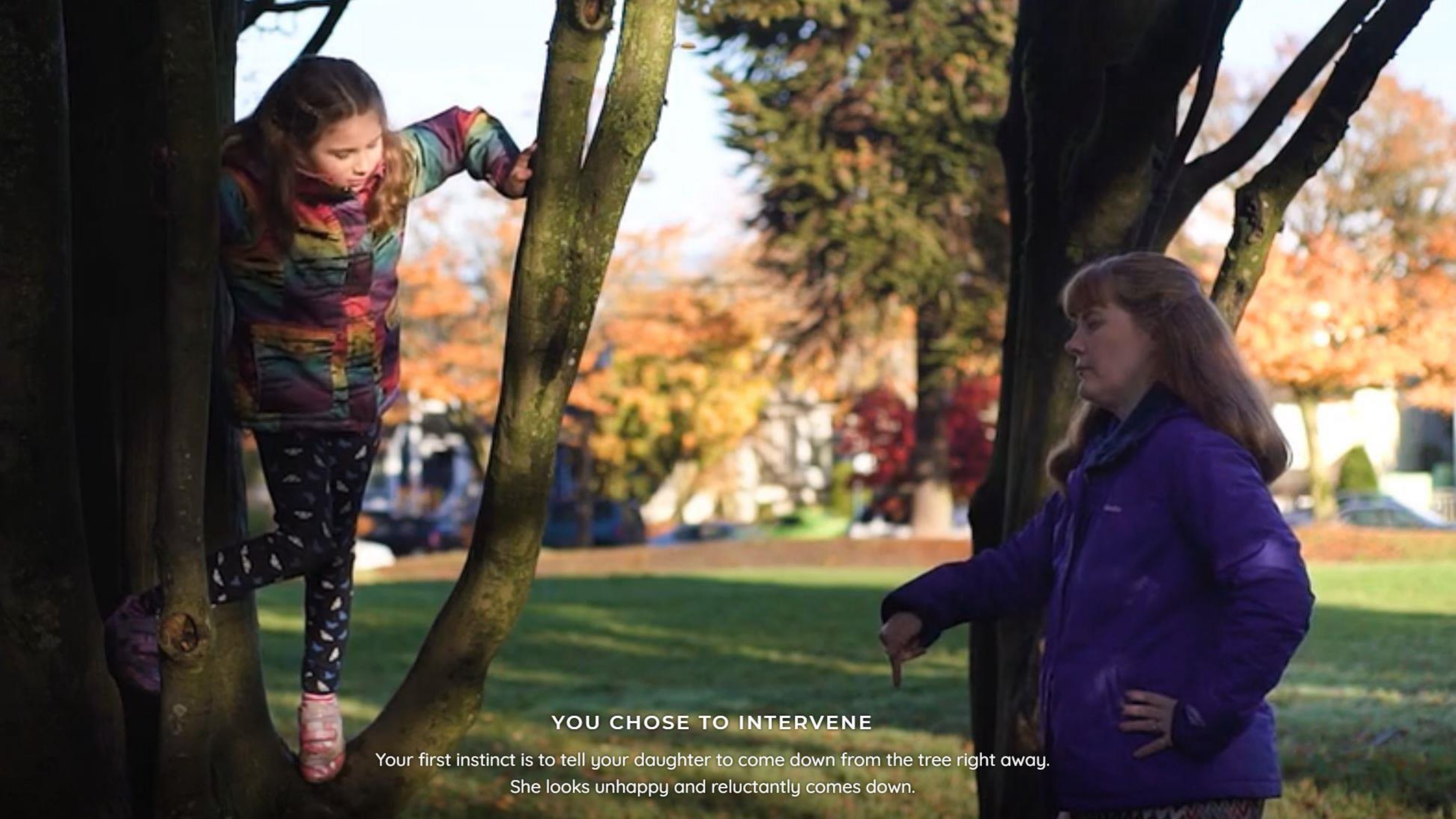
YOU CHOSE TO INTERVENE
Your first instinct is to tell your daughter to come down from the tree right away.
She looks unhappy and reluctantly comes down.

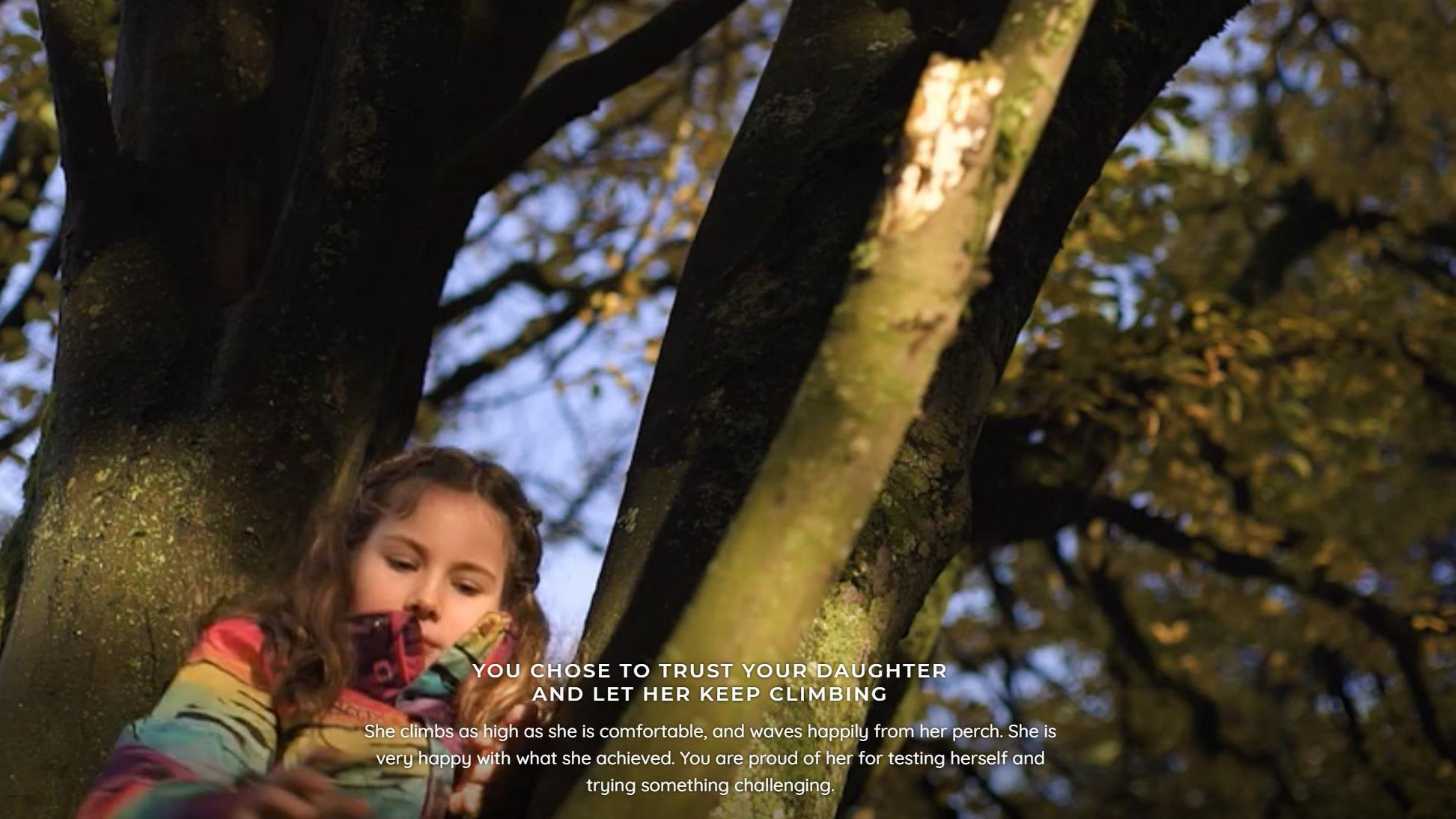
YOU CHOSE TO TRUST YOUR DAUGHTER
AND LET HER KEEP CLIMBING
She climbs as high as she is comfortable, and waves happily from her perch. She is very happy with what she achieved. You are proud of her for testing herself and trying something challenging.

WALKING HOME FROM SCHOOL

Imagine you and your son are walking home from school, a walk you've done together many times before.

Begin Scenario

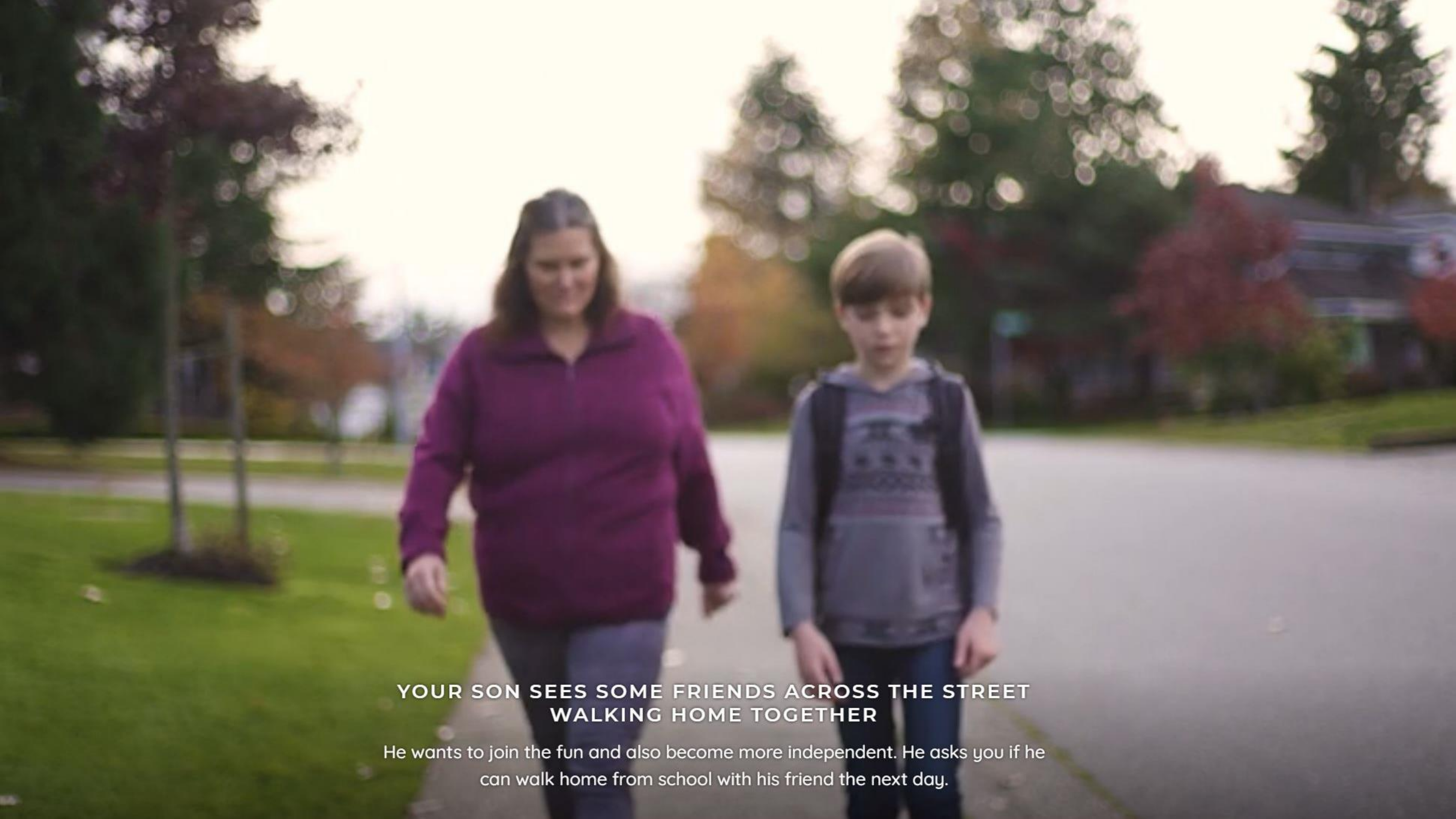

YOUR SON SEES SOME FRIENDS ACROSS THE STREET WALKING HOME TOGETHER

He wants to join the fun and also become more independent. He asks you if he can walk home from school with his friend the next day.

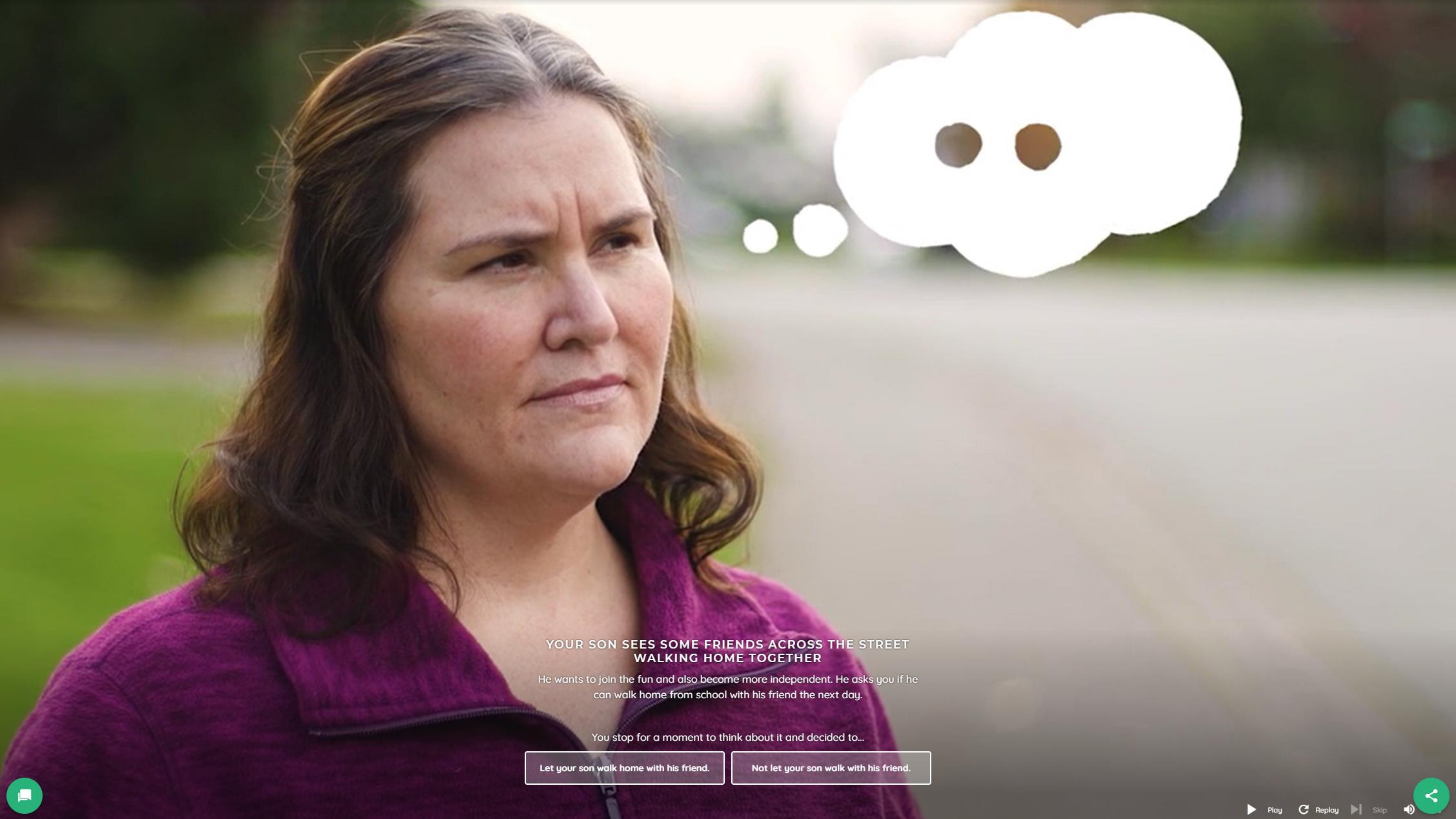

YOUR SON SEES SOME FRIENDS ACROSS THE STREET WALKING HOME TOGETHER

He wants to join the fun and also become more independent. He asks you if he can walk home from school with his friend the next day.

You stop for a moment to think about it and decided to...

Let your son walk home with his friend.

Not let your son walk with his friend.

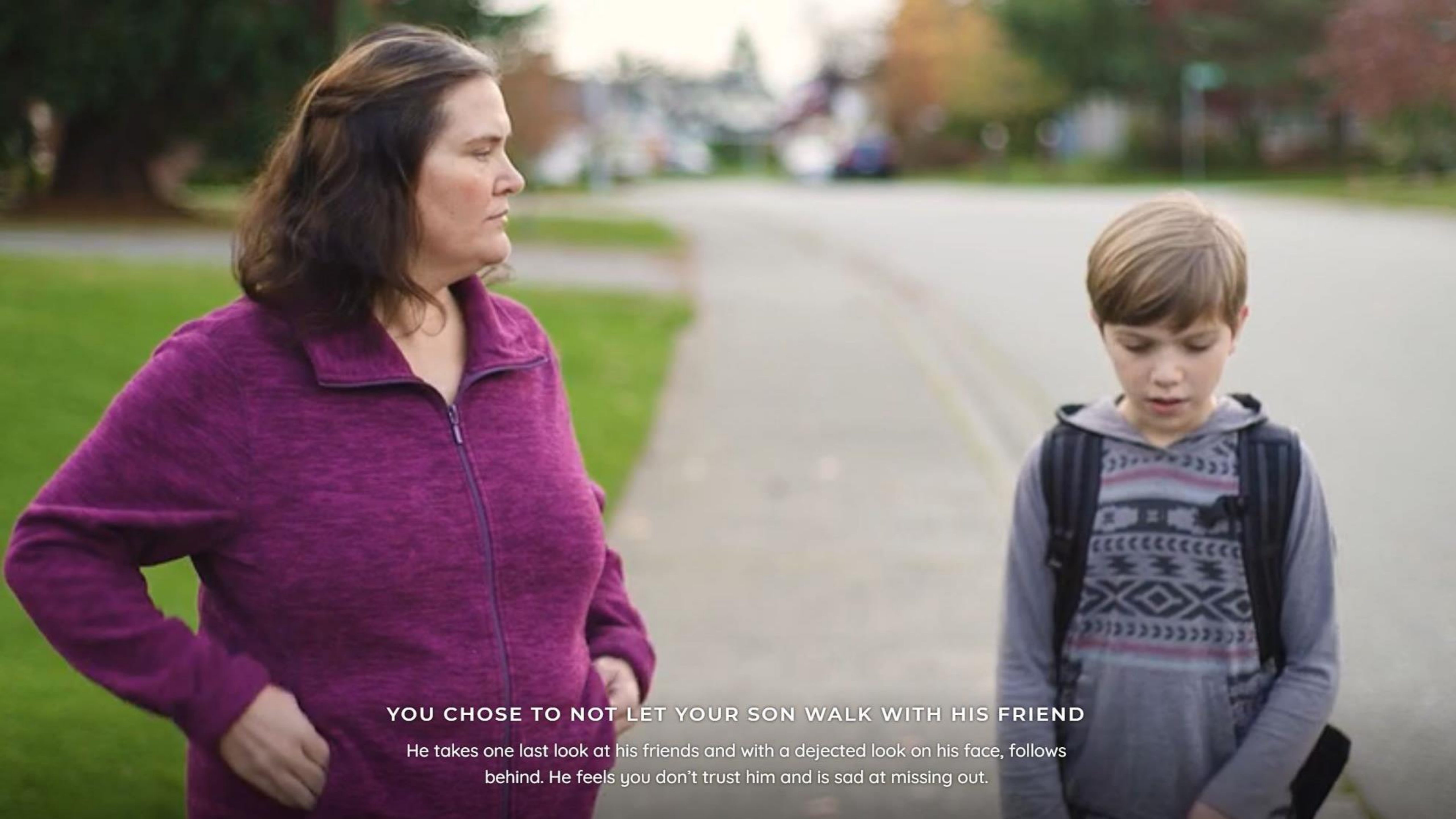
YOU CHOSE TO NOT LET YOUR SON WALK WITH HIS FRIEND
He takes one last look at his friends and with a dejected look on his face, follows behind. He feels you don't trust him and is sad at missing out.

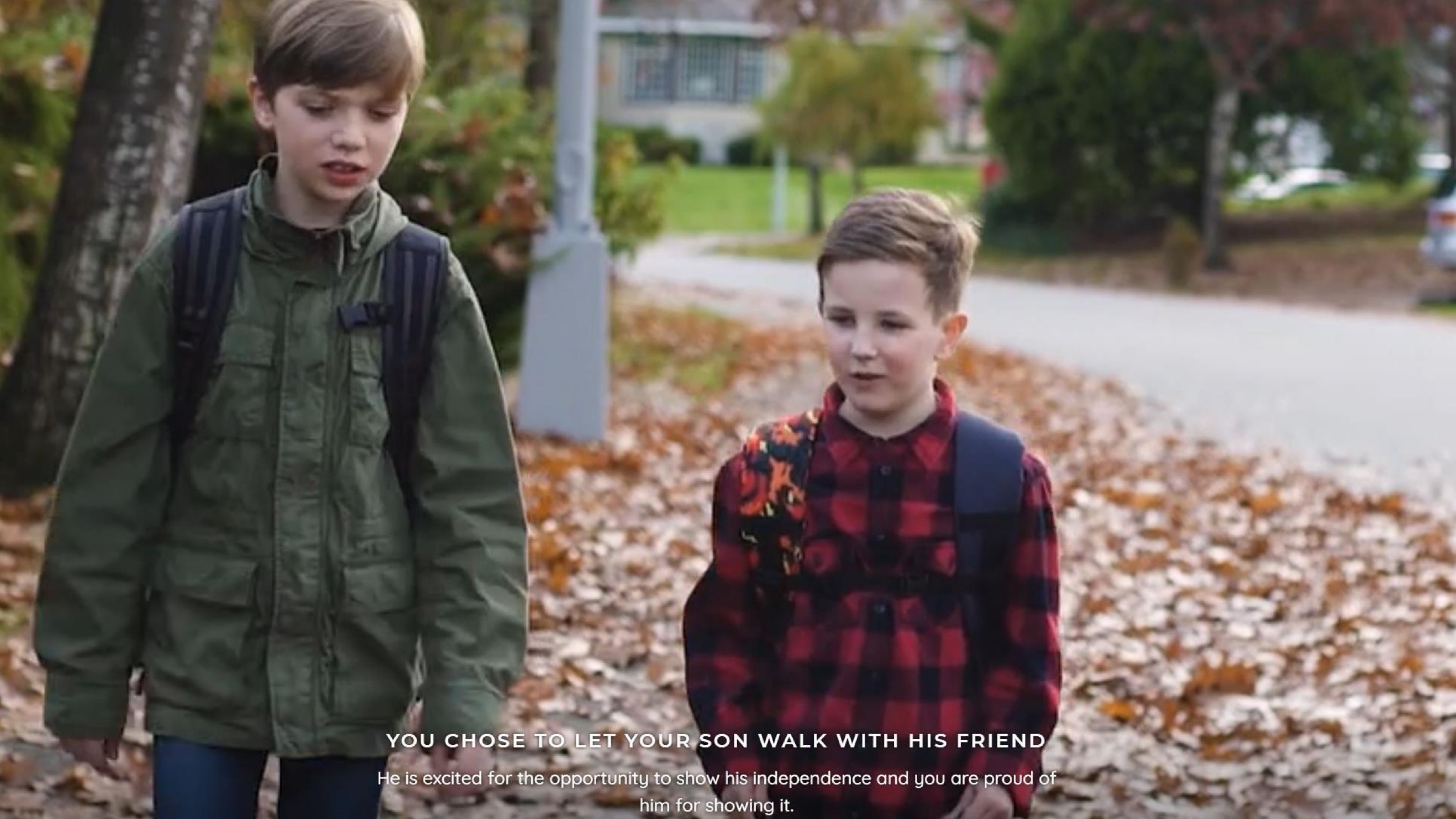
YOU CHOSE TO LET YOUR SON WALK WITH HIS FRIEND
He is excited for the opportunity to show his independence and you are proud of him for showing it.

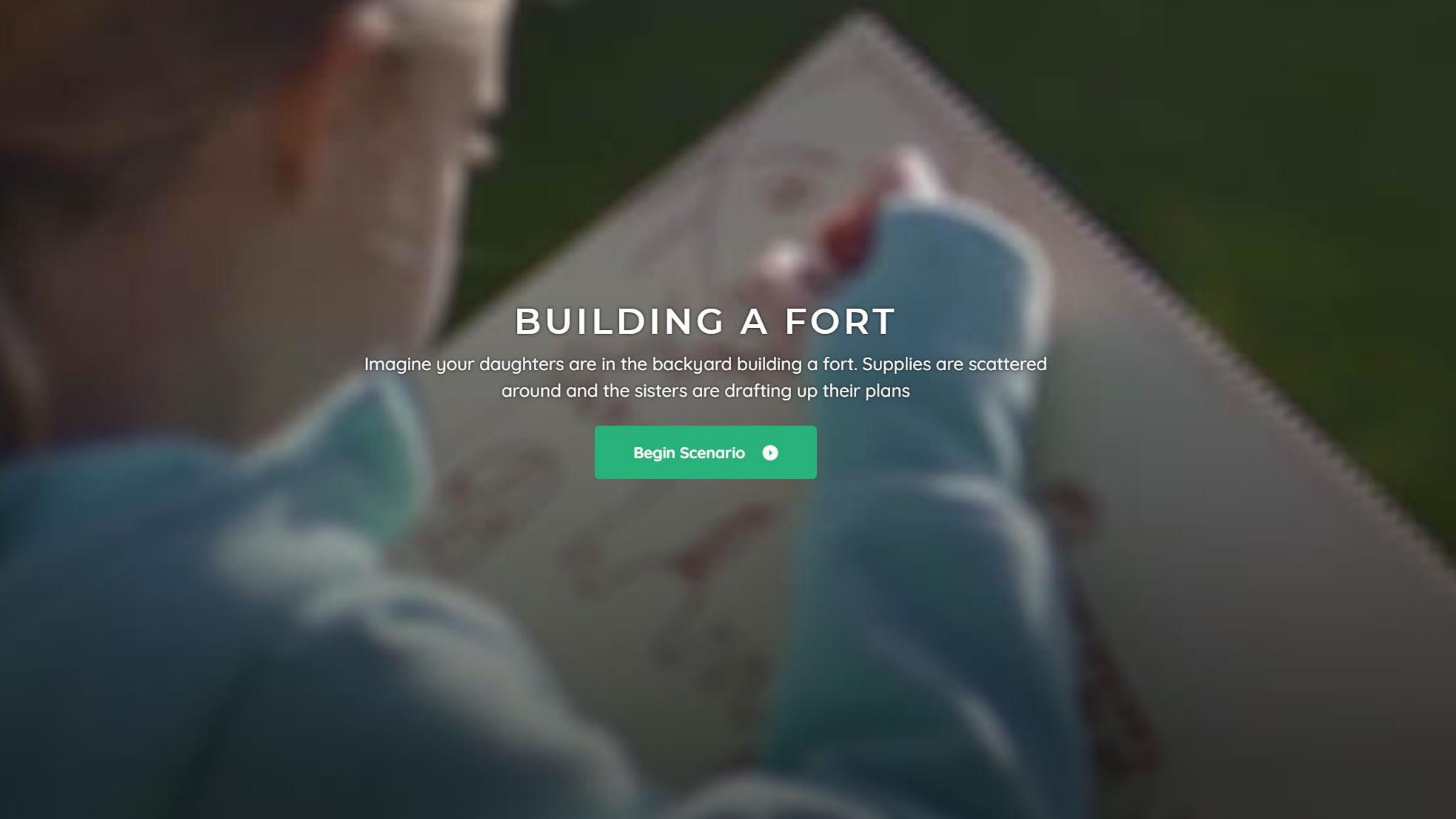

BUILDING A FORT

Imagine your daughters are in the backyard building a fort. Supplies are scattered around and the sisters are drafting up their plans

Begin Scenario

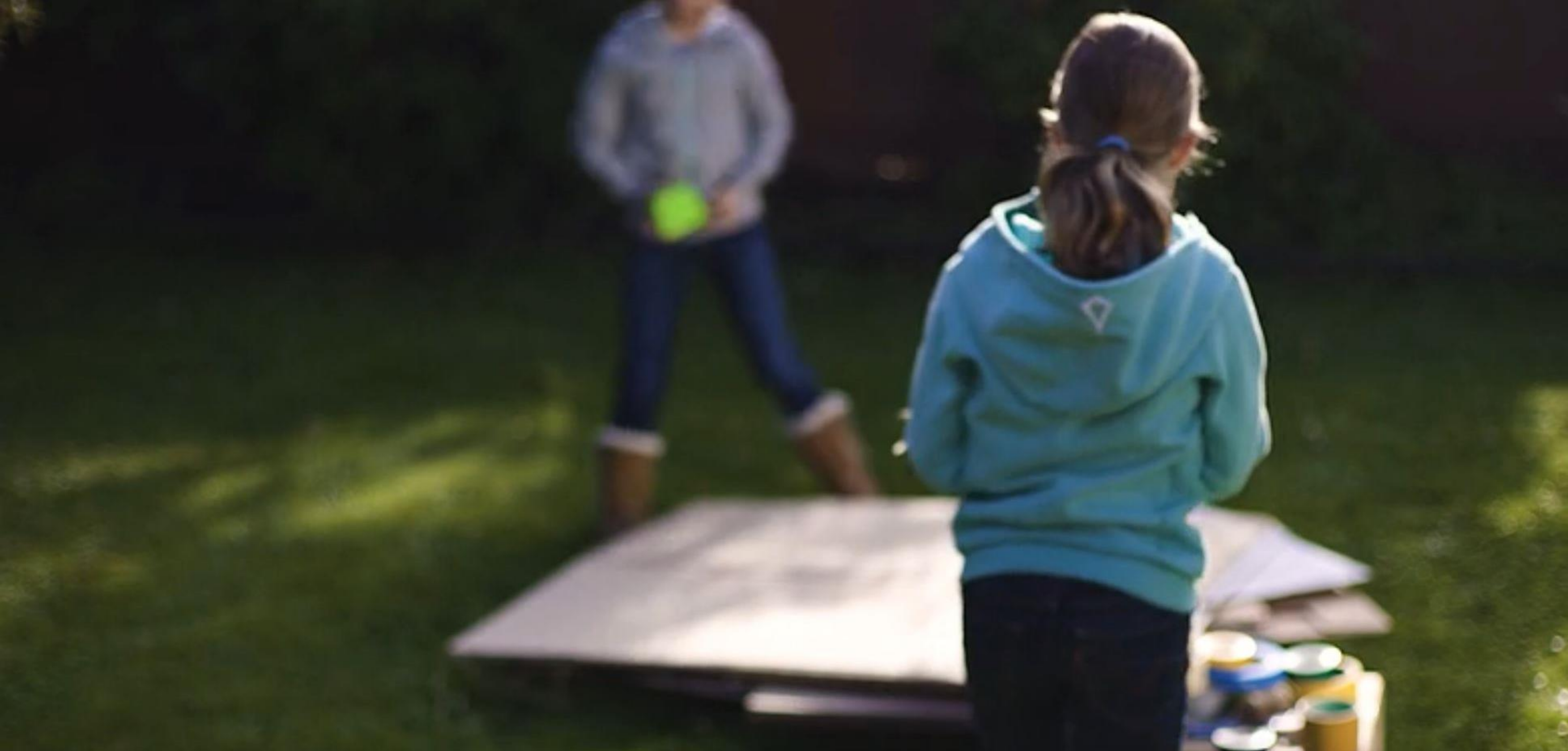

THE SISTERS WOULD LIKE TO CUT SOME WINDOWS

They realized they don't have anything to cut the windows out of the fort. Your daughter walks over to you and explains how they'll be needing the box cutters to cut the windows out of fort for their plan to work.

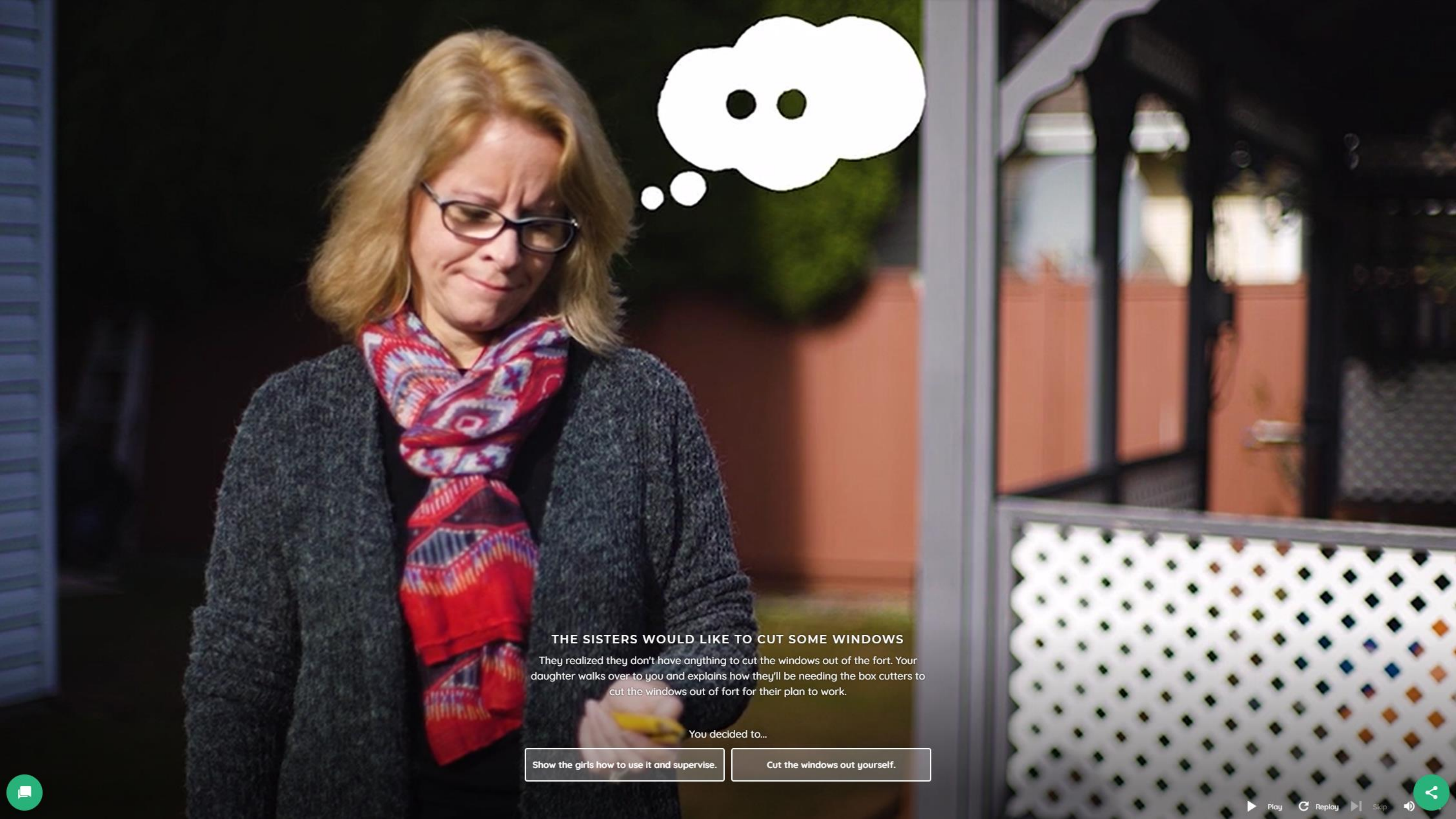
THE SISTERS WOULD LIKE TO CUT SOME WINDOWS
They realized they don't have anything to cut the windows out of the fort. Your daughter walks over to you and explains how they'll be needing the box cutters to cut the windows out of fort for their plan to work.
You decided to...
Show the girls how to use it and supervise.
Cut the windows out yourself.
Play
Replay
Skip

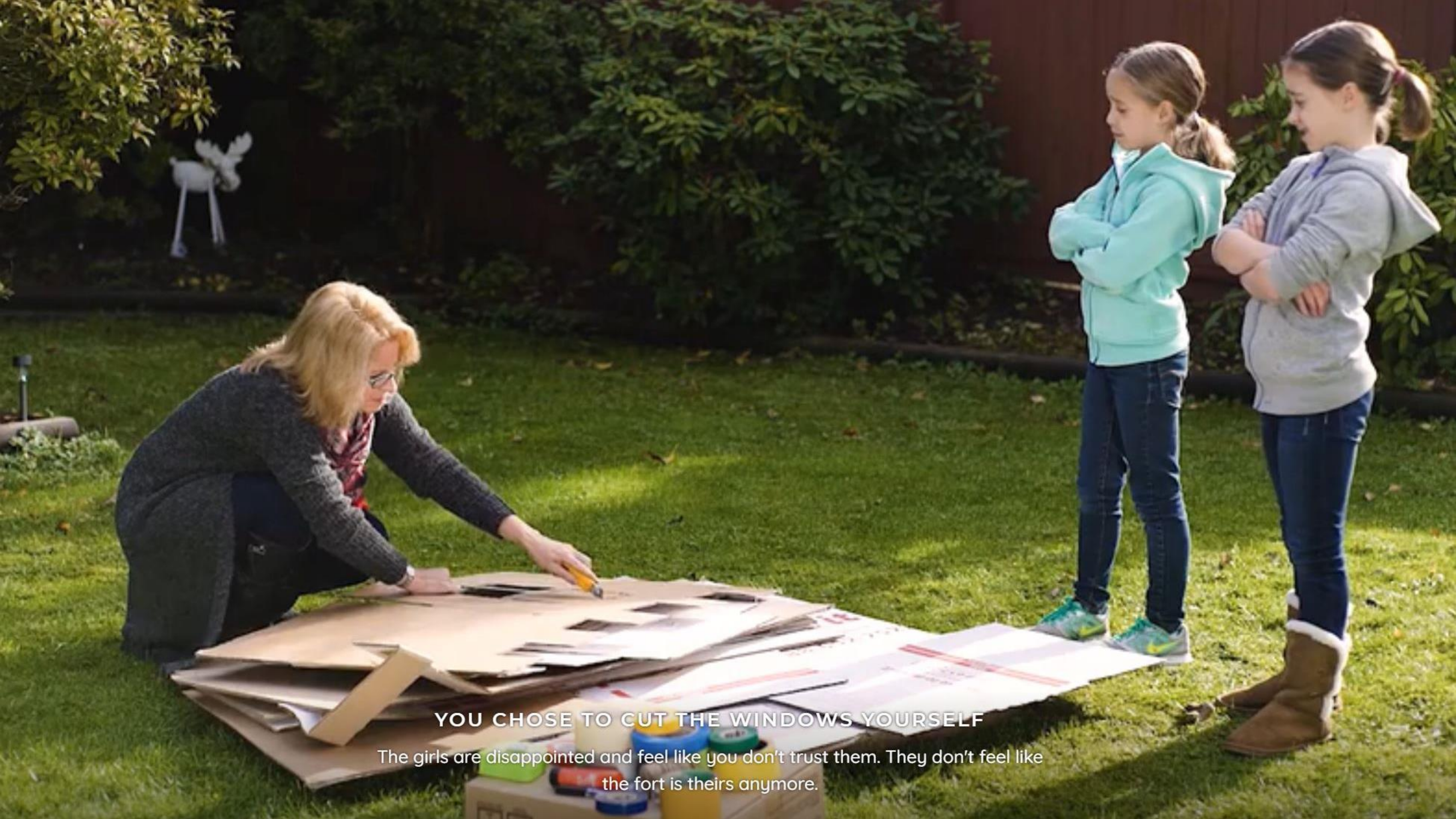
YOU CHOSE TO CUT THE WINDOWS YOURSELF
The girls are disappointed and feel like you don't trust them. They don't feel like the fort is theirs anymore.

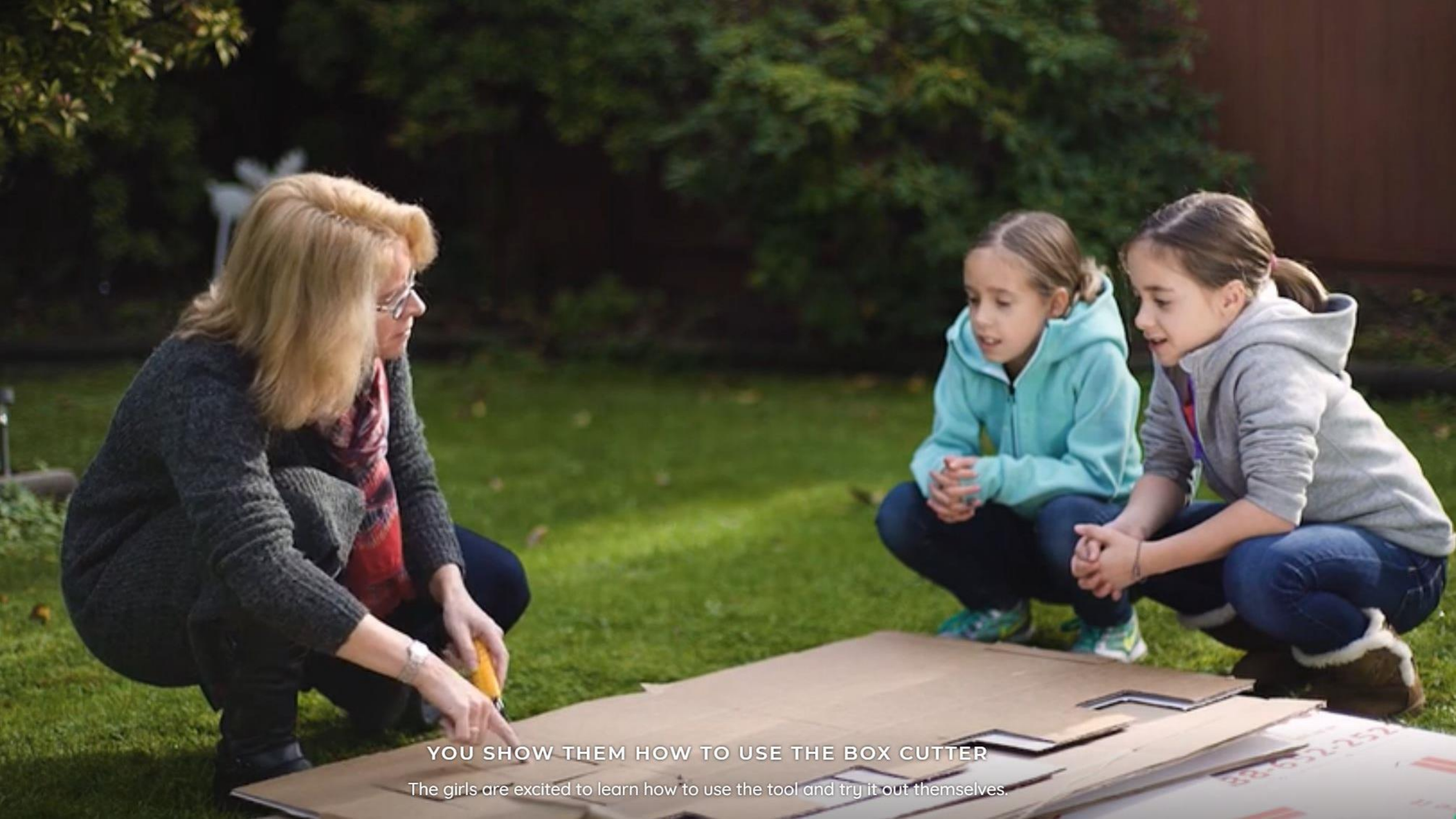
YOU SHOW THEM HOW TO USE THE BOX CUTTER
The girls are excited to learn how to use the tool and try it out themselves.

COMMON CONCERNS

As parents, our fears can sometimes get in the way of our decisions about the things we let our kids do. In situations like the ones in these scenarios, what are some of the fears that have gotten in your way before?

CLICK ON ANY THOUGHT BUBBLES THAT APPLY TO YOU:

I am concerned my child is going to get seriously hurt.

I think my child does not know how to stay safe.

I am worried that someone is going to harm my child, either kidnapping or assault by an adult, or bullying by other children.

I am concerned that other people are going to think I am a bad parent for letting my child take risks.

THINGS THAT HELPED ME LET GO

In situations like the ones in these scenarios, what are some of the things that helped you let my child keep going.

CLICK ON ANY THOUGHT BUBBLES THAT APPLY TO YOU:

I have seen my child manage things like this before and trust that my child can handle it.

I believe my child really wants to do this and I want to give my child my support.

It is important to me that my child has opportunities to learn, build skills and try new challenges.

I know my child really wants to do this and I think it is a good learning opportunity.

Chapter 3

CREATING YOUR PLAN

To help you create your plan for what you want to do next, here's an overview of your journey and decisions so far.

Next

Chapter 1 - Reflection

Revisit

Think about a child you care about (between the age of 6 - 12 if possible). This is the child you'll be thinking about during your journey, and could be your son or daughter, a student, or another child close to you.

Child's Name	my child
Child's Age	6
Child's Gender	Girl
I am the..	Mother

Value & Traits

What are some of the most important attributes I want **my child** to have when she grows up?

my child's Activities

What are some of my child's favourite things to do on their own or with friends?

Your Childhood Activities

What were some of your favourite things to do on your own or with friends?

What did you get out of it?

Think back to what you learned from these experiences and how they influenced you. What did you get out of these play activities?

Same or Different?

How do my child's activities compare to what you remember doing at that age?

My childhood activities	Activities we share	my child's Activities
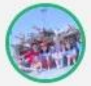	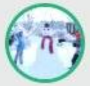	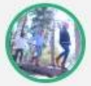

What I got out of it	What I want for my child
Compassionate Joyful	Courageous Resilient Imaginative

Chapter 2 - What would you do?

Imagine yourself as the parent in each interactive video segment. Think about what you would do in these situations. Your choices will impact what happens in the story and the outcomes. Reflect on the factors that influenced your decisions and discover what you could have done differently.

Revisit

Climbing a Tree

Imagine you and your daughter are going for an afternoon walk around a park. You decided to take a break and sit on a nearby bench while your daughter begins to play around some trees.

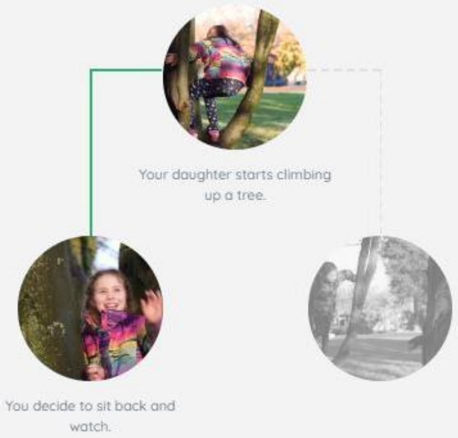

Revisit

Walking Home from School

Imagine you and your son are walking home from school, a walk you've done together many times before.

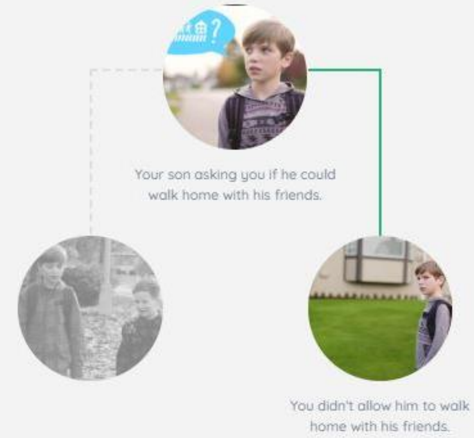

Revisit

Building a Fort

Imagine your two daughters are in the backyard building a fort. Supplies are scattered around and the sisters are drafting up their plans.

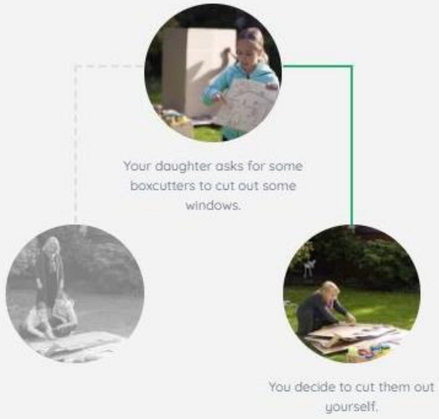

Common Concerns

As parents, our fears can sometimes get in the way of our decisions about the things we let our kids do. In situations like the ones in these scenarios. What are some of the common concerns you may have?

Things That Helped Me Let Go

In situations like the ones in these scenarios, what are some of the things that helped you let my child keep going.

Take a risk, go play outside!

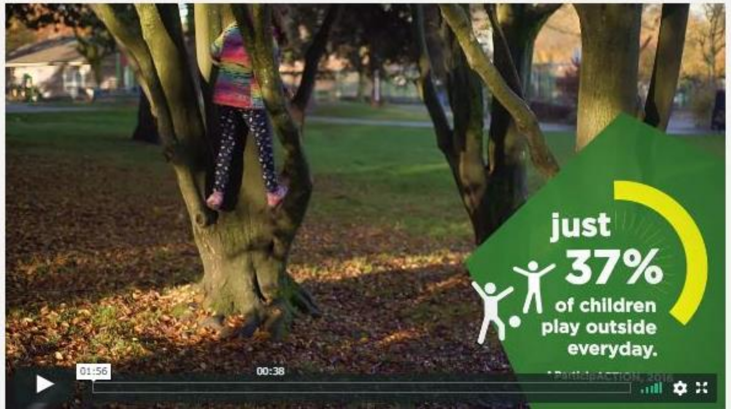

Chapter 3 - My Plan of Action

As you revisit what you want most for my child when she grows up, think about what you are doing to promote those things. Is there anything you want to change?

Add Goal

SETTING GOALS

You have the opportunity to make a change based on your journey. Set yourself one goal. It can be whatever you like, as long as it is realistic and doable for you. For your goal, think about the different steps that can help you make the change.

MY GOAL IS TO...

Examples

- Letting my child climb up a tree
- Letting my child use more tools
- Letting my child play out in the yard without supervision
- Letting my child walk to school
- Make time for my child to play outside every day
- Encourage my child to play outside independently.

STEPS I WOULD TAKE TO ACHIEVE MY GOAL ARE...

- Share this tool with my partner and talk about the importance of risky play
- Guide my child through new activity while coaching on safety
- Watch without interfering
- Take away supervision gradually

Add your own step

Add Step

I WILL BEGIN MY PLAN...

Jul 28 2020

Next

MY PLAN OF ACTION

Letting my child use more tools

Start Date: Jul 27, 2020
Steps:

- Share this tool with my partner and talk about the importance of risky play
- Guide my child through new activity while coaching on safety
- Watch without interfering
- Take away supervision gradually

Edit Goal | Delete Goal

Letting my child play out in the yard without supervision

Start Date: Jul 27, 2020
Steps:

- Guide my child through new activity while coaching on safety
- Take away supervision gradually
- Share this tool with my partner and talk about the importance of risky play
- Watch without interfering

Edit Goal | Delete Goal

+

Add New Goal

FINISH MY ACTION PLAN

Once you're satisfied with your action plan, you can share or print it out

YOUR JOURNEY IS ONLY GETTING STARTED!

Thank you for taking the time to go on this personal journey. Keep your action plan somewhere that it can be easily seen (eg. On your fridge or at your desk) as a reminder.

DOWNLOAD SUMMARY & ACTION PLAN

Click the button below to download a PDF copy of your journey map and action plan.

Download PDF

OR

EMAIL JUST ACTION PLAN

Send **just** your action plan to an email by entering it below and hitting send! It could be to yourself or to a family/friend.

sample@123.com

Send Email

Note: Your email is only used to send you your action plan and is not being stored.

Please note that **your progress will not be saved** if you **refresh the page** or **navigate off this website.**
