# Supplementary material for: A Web-Based and In-Person Risk Reframing Intervention to Influence Mothers’ Tolerance for, and Parenting Practices Associated With, Children’s Outdoor Risky Play: Randomized Controlled Trial
Source: J Med Internet Res. 2021 Apr 27;23(4):e24861. doi: 10.2196/24861 (PMC8114163; doi:10.2196/24861)
Supplement: Multimedia Appendix 3 [file jmir_v23i4e24861_app3.pdf]

Home Journey Map FAQ About Us

OUTSIDEPLAY.ca

Take a risk, go play outside!

An online tool to help parents and caregivers gain the confidence to allow their kids to engage in more outdoor play.

Click to watch video!

BC Children's Hospital | UBC | BC INJURY research and prevention unit | SIO LAB

Already know what Risky Play is and want to get started?

Start Journey

Icebreaker!

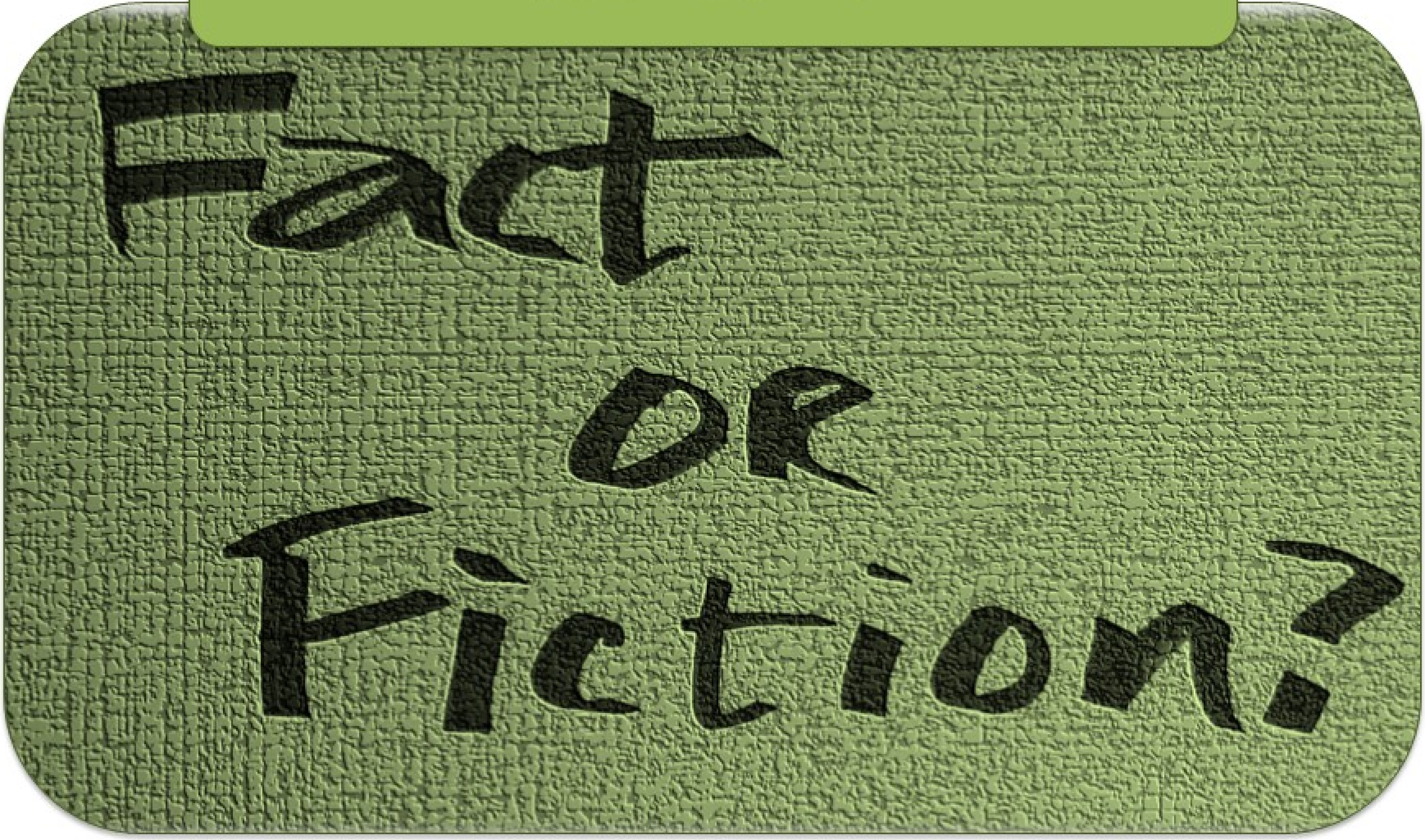

OUTSIDEPLAY.ca

Take a risk, go play outside!

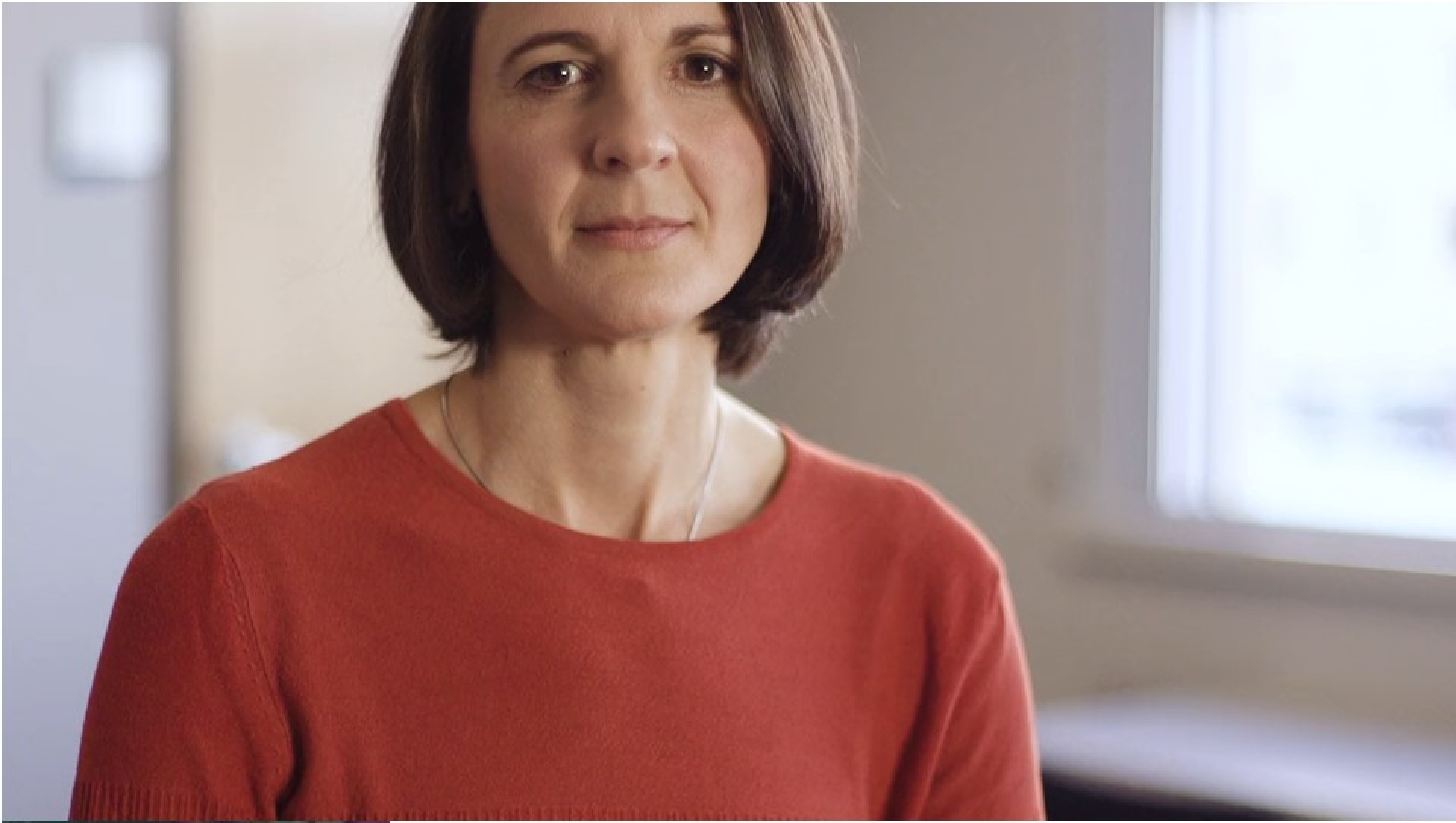

BC Children's Hospital UBC BC INJURY research and prevention unit SIO LAB

Introduction

(Worksheet 1-1)

Q

1-1

Introduction
Think about your child (between the age of 6-12). This is the child you'll be thinking about during your journey, and could be your son or daughter.

Child's first name/nickname: ________________________________.

Child's age: ___________.

Child's gender: _____________.

Values & Traits

(Worksheet 1-2)

1-2

Values & Traits

The most important attributes I want my child to have when they grow up are:

1 ____

2 ____

3 ____

Child's Activities

(worksheet 1-3)

Q

Think about your child's favourite things to do on their own or with friends.

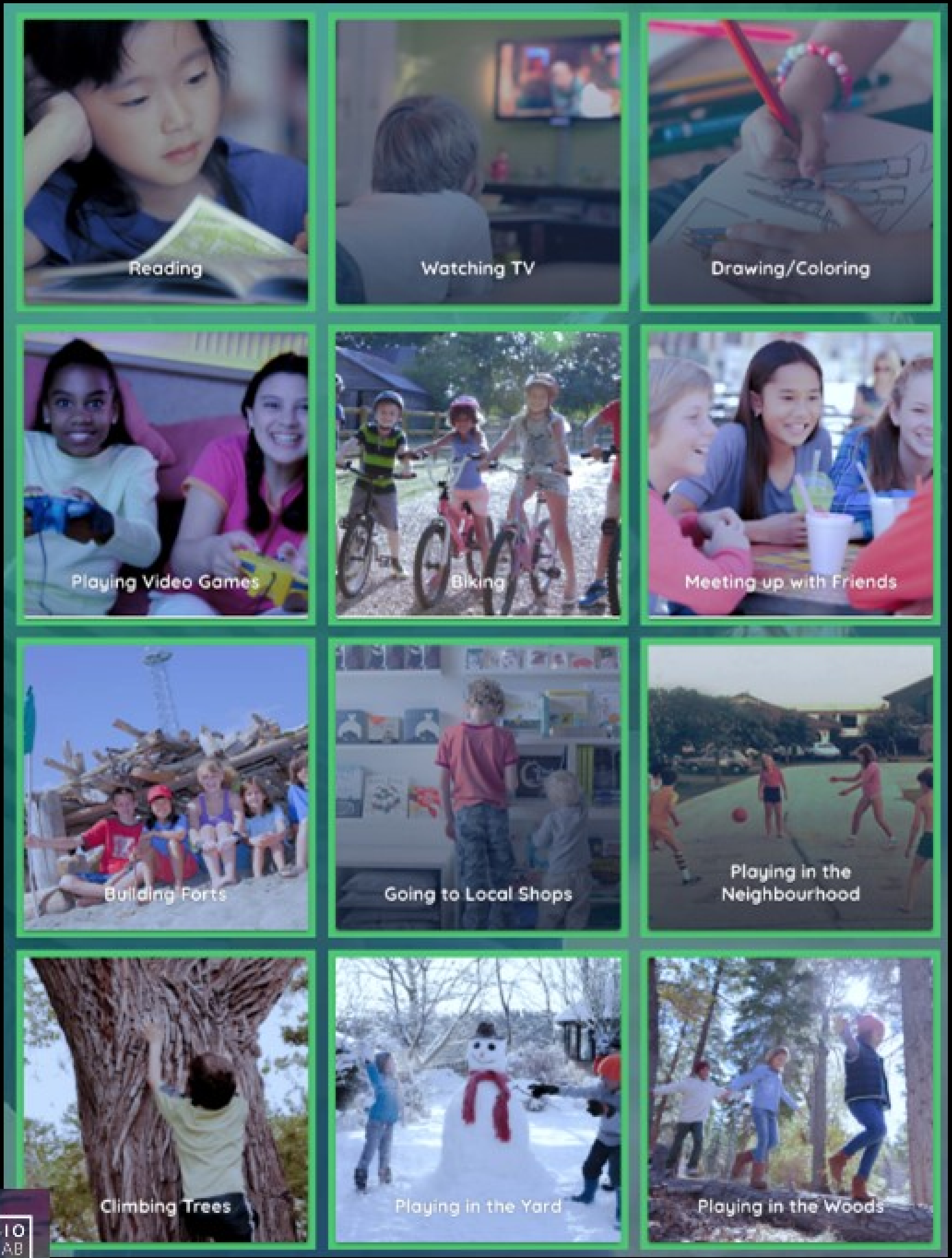
Reading
Watching TV
Drawing/Coloring
Playing Video Games
Biking
Meeting up with Friends
Building Forts
Going to Local Shops
Playing in the Neighbourhood
Climbing Trees
Playing in the Yard
Playing in the Woods

Your Activities

(worksheet 1-4)

Q

Think about your childhood play activities…

What Did You Get Out of It?
(worksheet 1-5)

- Think back to what you learned from these experiences and how they influenced you?
- What did you get out of these play activities?
- What made them fun?

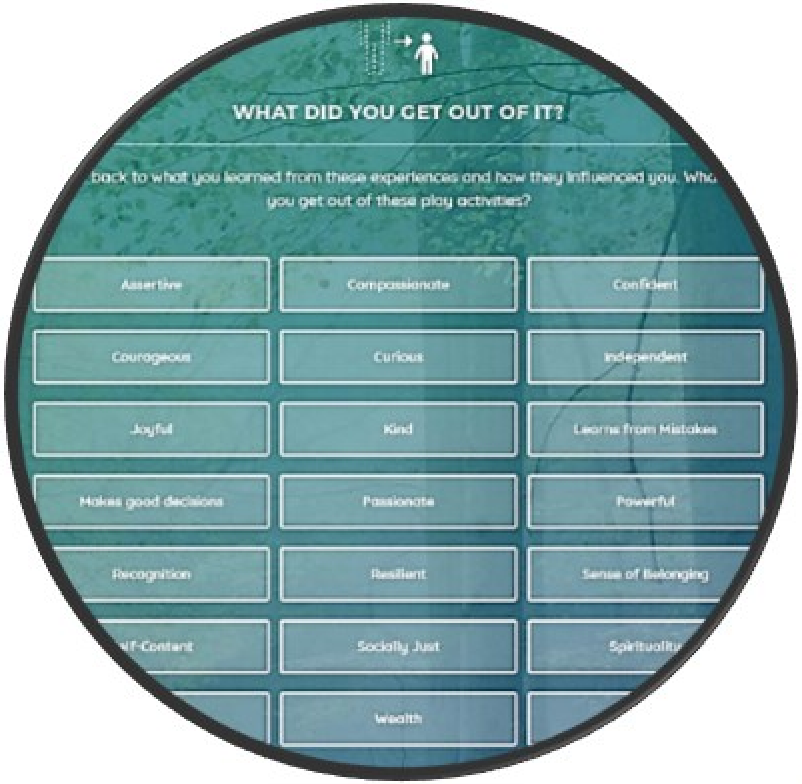

WHAT DID YOU GET OUT OF IT?

Think back to what you learned from these experiences and how they influenced you. What did you get out of these play activities?

Assertive	Compassionate	Confident
Courageous	Curious	Independent
Joyful	Kind	Learns from Mistakes
Makes good decisions	Passionate	Powerful
Recognition	Resilient	Sense of Belonging
Self-Content	Socially Just	Spirituality
Imaginative	Wealth	Wisdom

Same or Different?

(Worksheet 1-6)

Q

1-6 **Same or different?**

How do my child's activities compare to what I remember doing at that age?

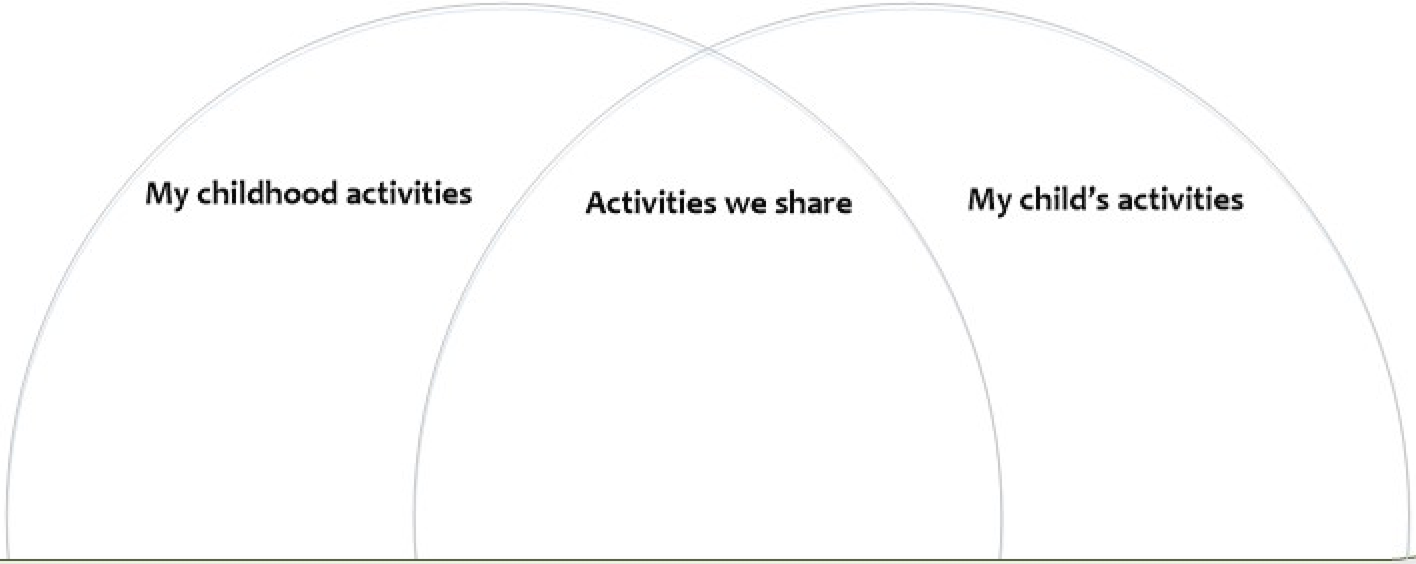

What Would You Do?

Q

- Imagine yourself as the parent in each scenario.

- Think about what you would do in these situations.

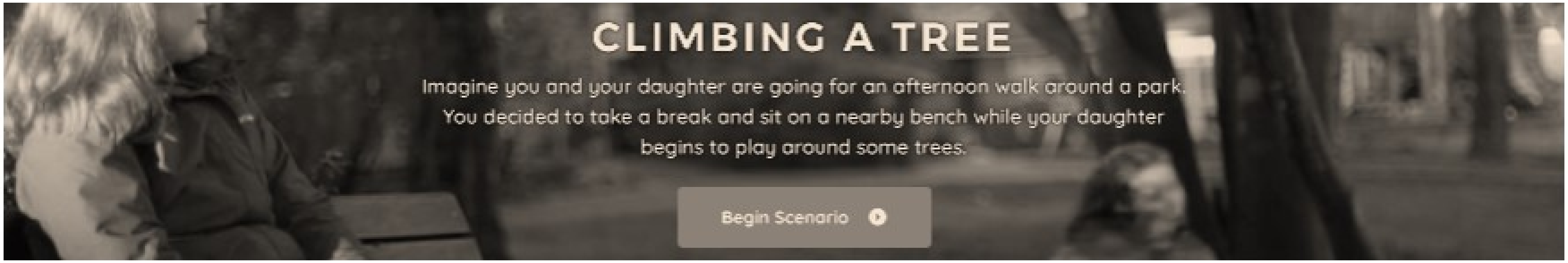
CLIMBING A TREE
Imagine you and your daughter are going for an afternoon walk around a park.
You decided to take a break and sit on a nearby bench while your daughter
begins to play around some trees.
Begin Scenario

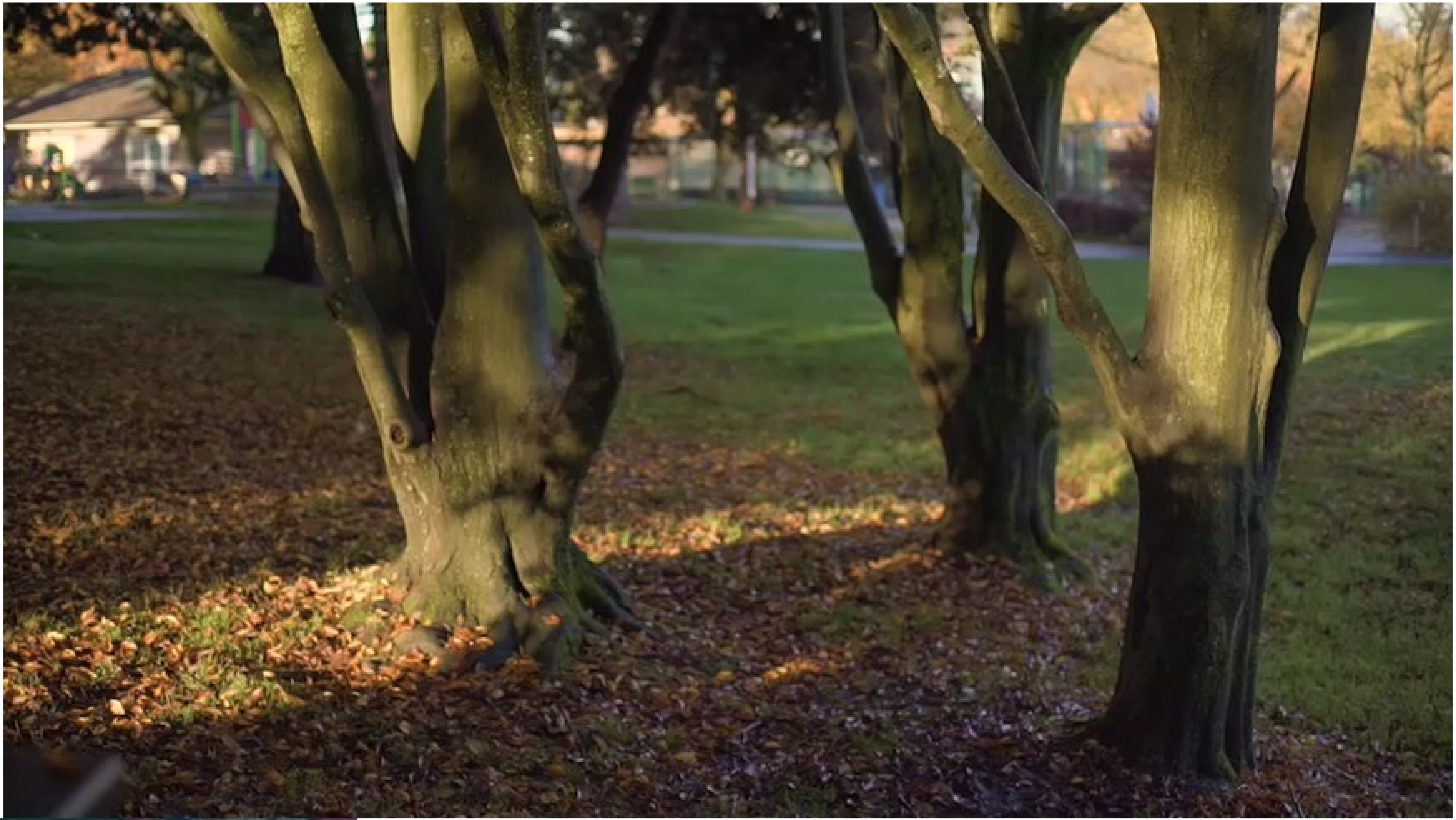

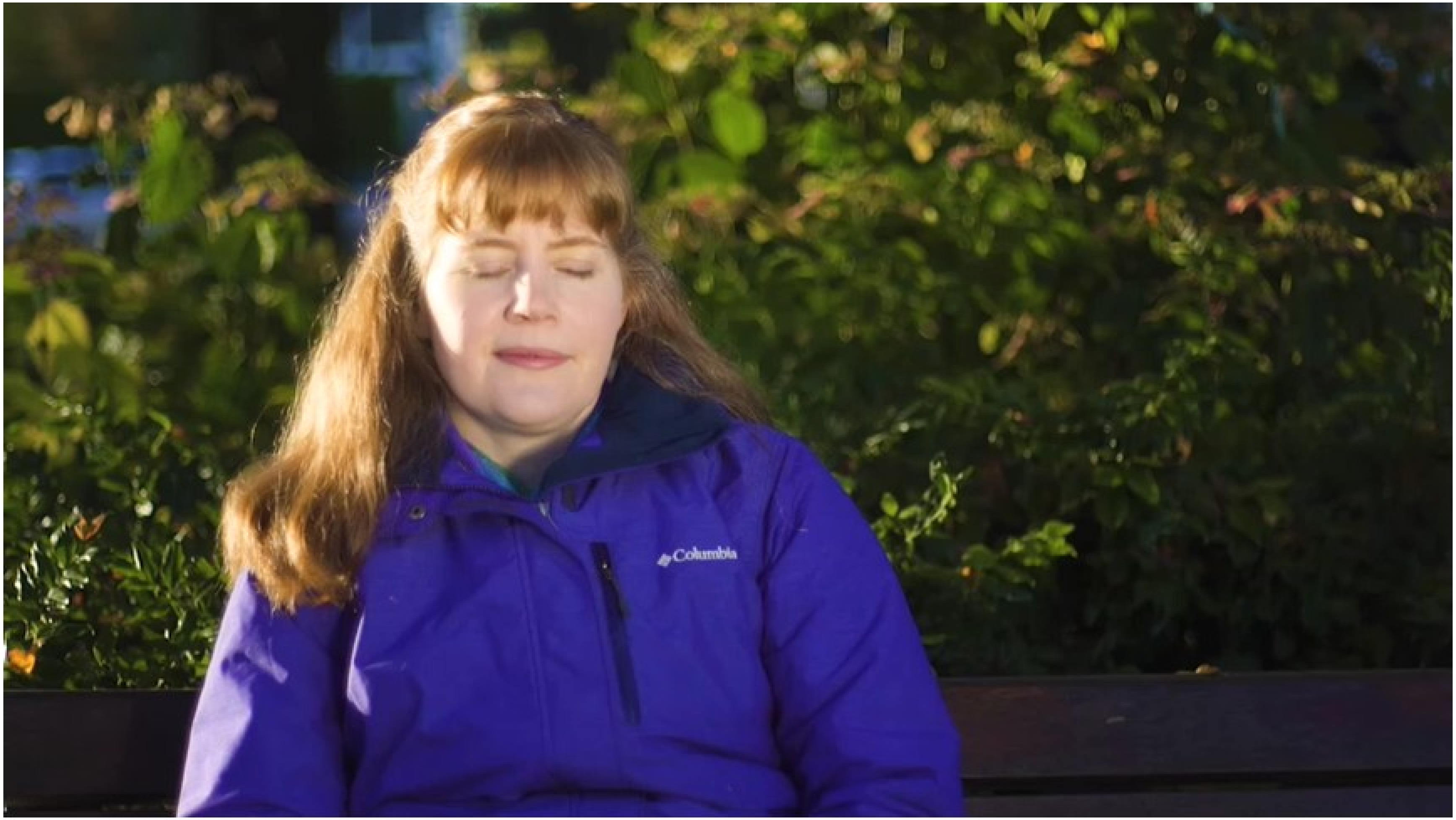

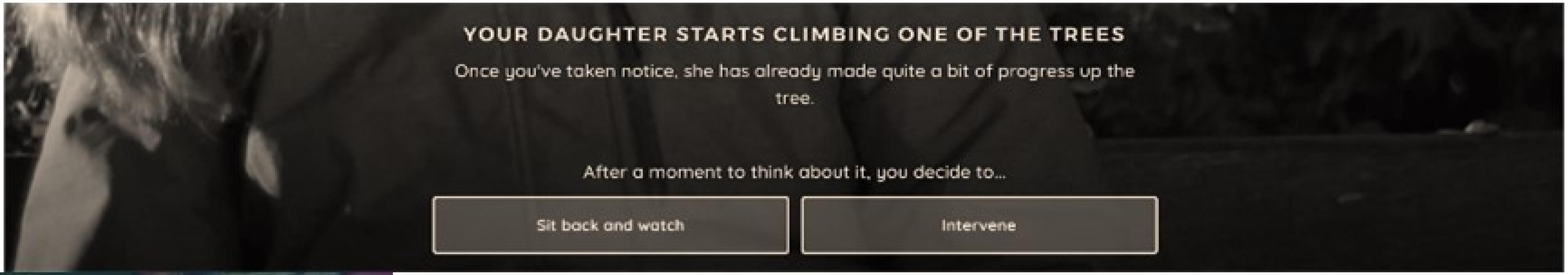
YOUR DAUGHTER STARTS CLIMBING ONE OF THE TREES
Once you've taken notice, she has already made quite a bit of progress up the tree.
After a moment to think about it, you decide to...
Sit back and watch
Intervene

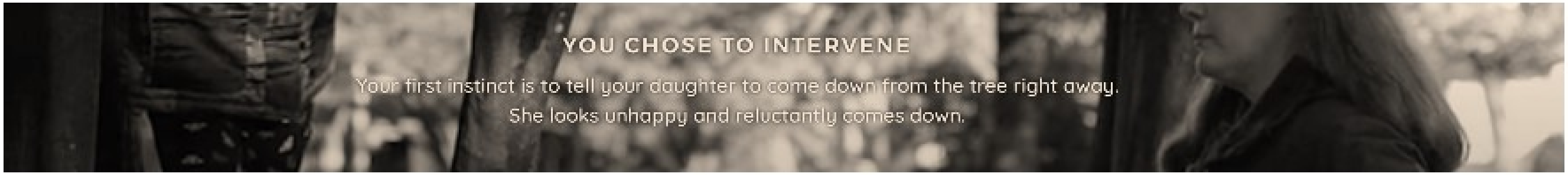
YOU CHOSE TO INTERVENE
Your first instinct is to tell your daughter to come down from the tree right away.
She looks unhappy and reluctantly comes down.

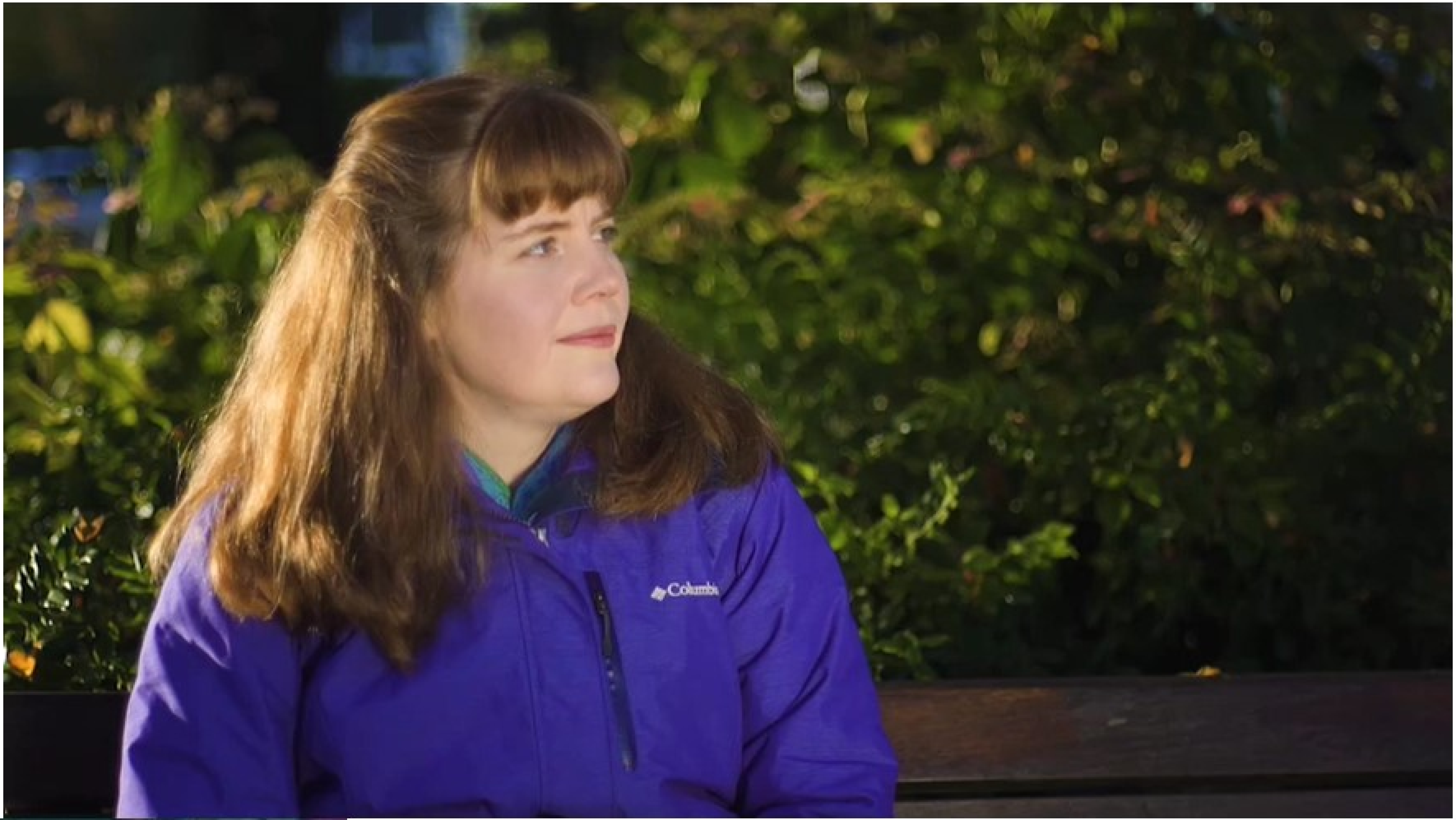

YOU CHOSE TO TRUST YOUR DAUGHTER AND LET HER KEEP CLIMBING

She climbs as high as she is comfortable, and waves happily from her perch. She is very happy with what she achieved. You are proud of her for testing herself and trying something challenging.

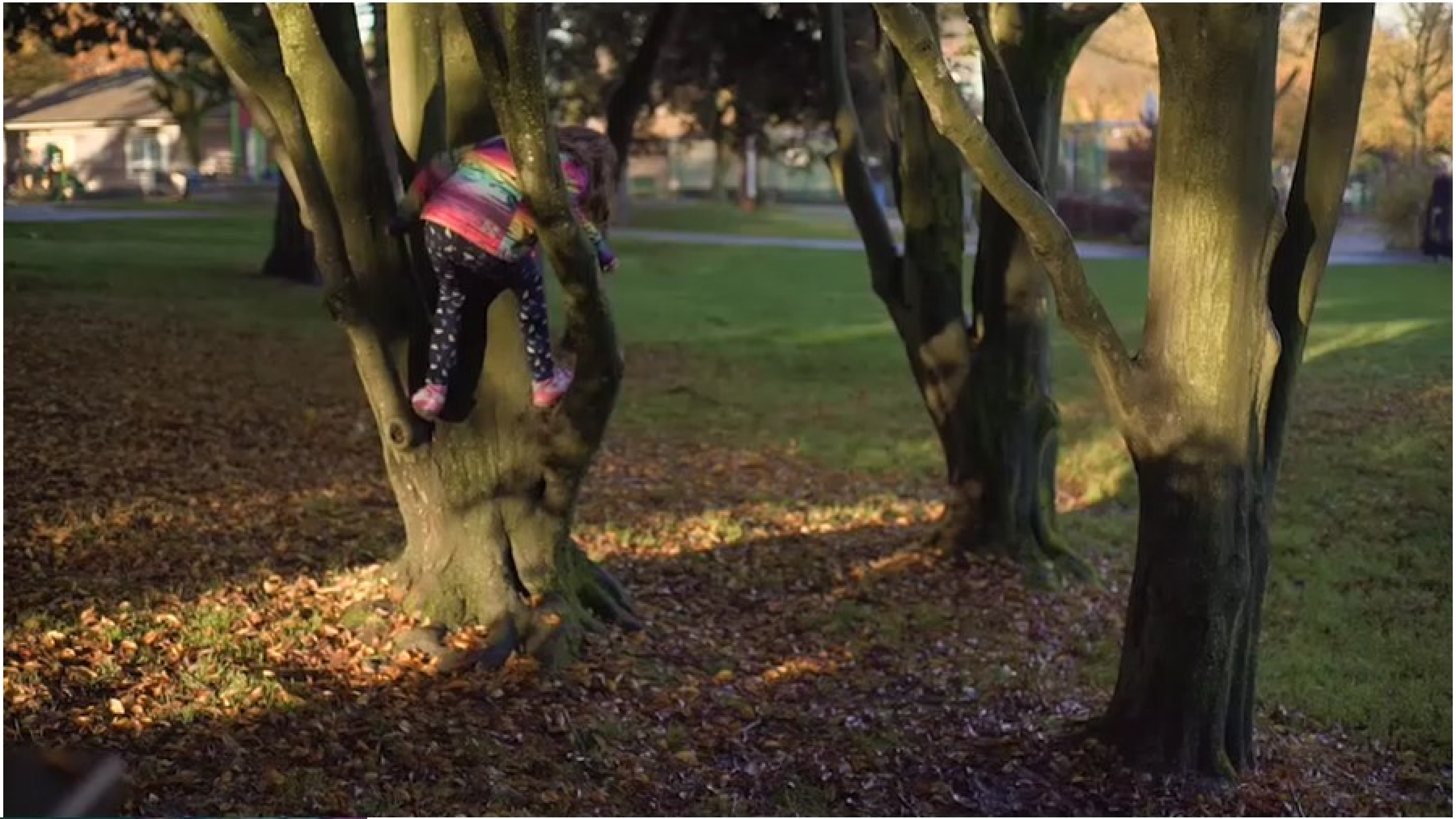

BC Children's Hospital UBC BC INJURY research and prevention unit SIO LAB

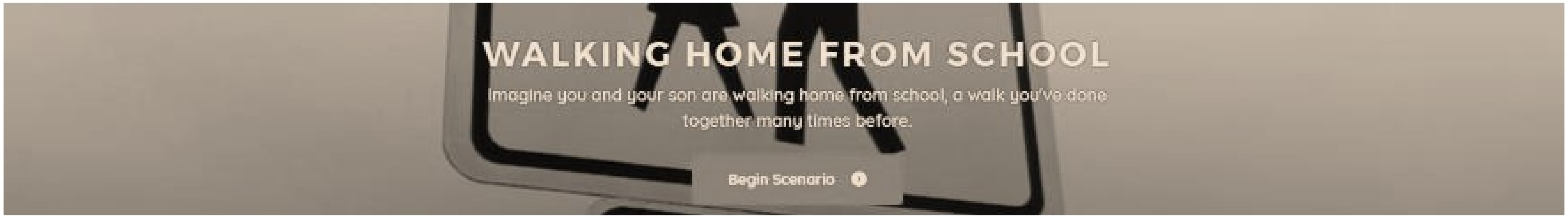

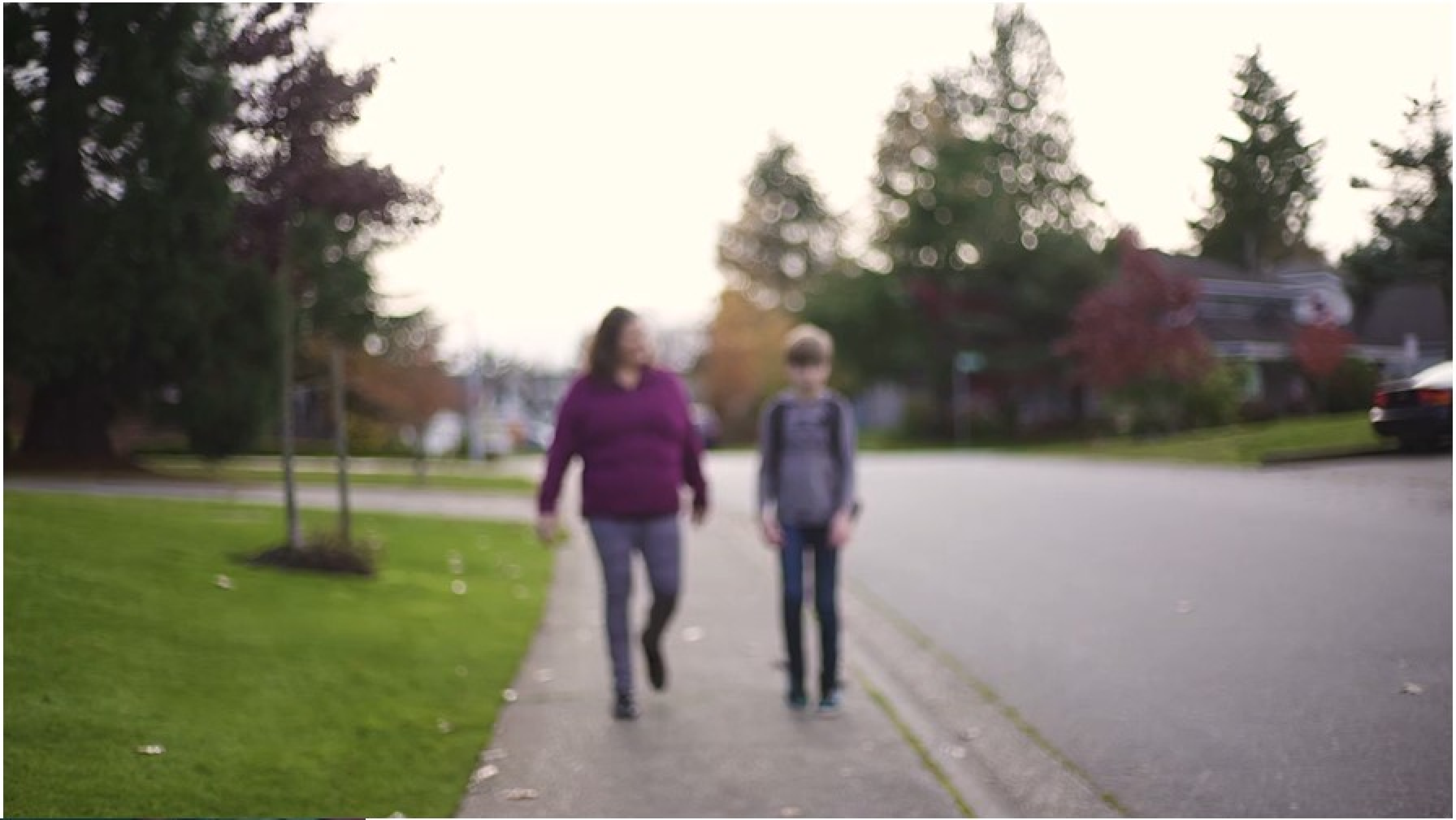

BC Children's Hospital UBC BC INJURY research and prevention unit SIO LAB

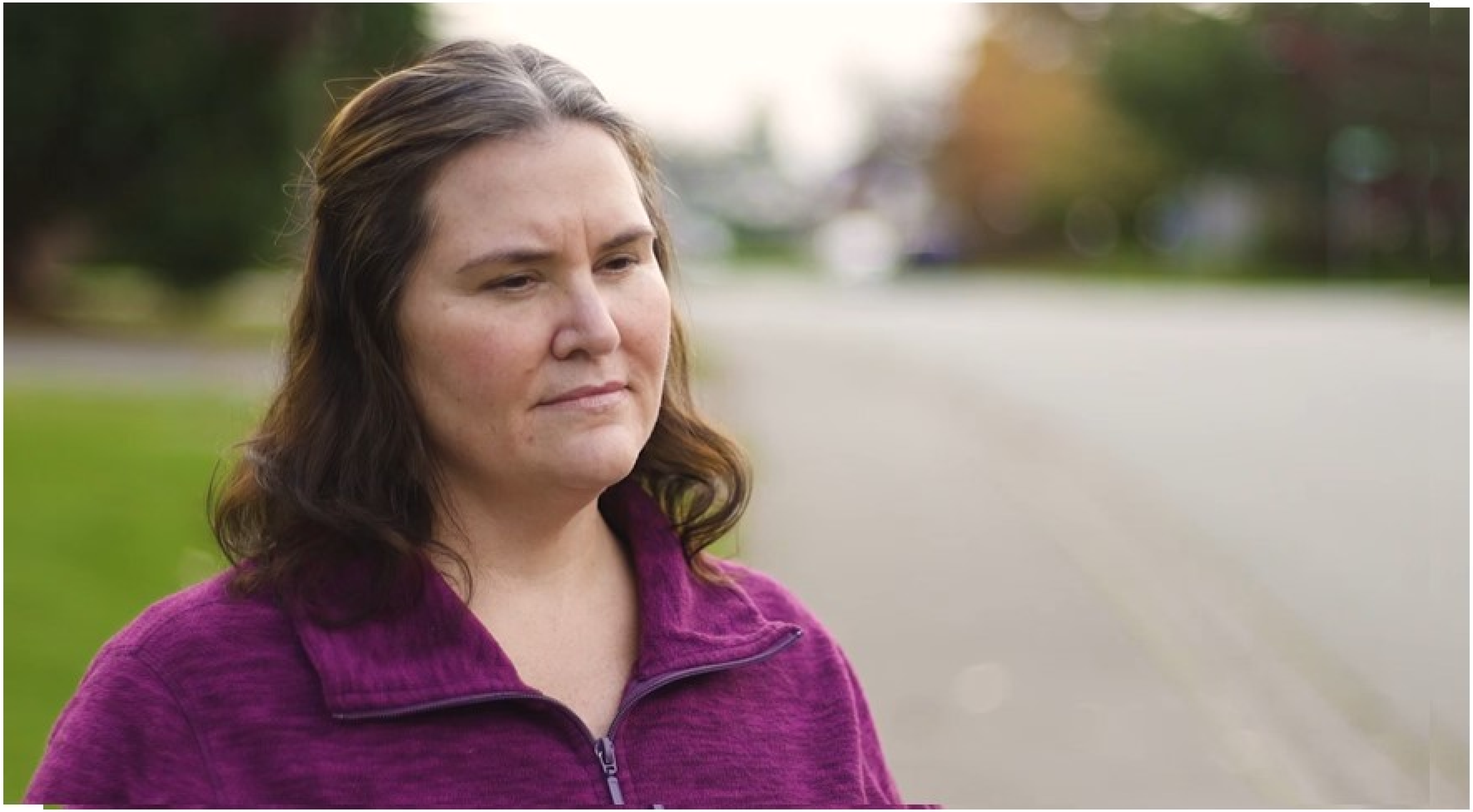

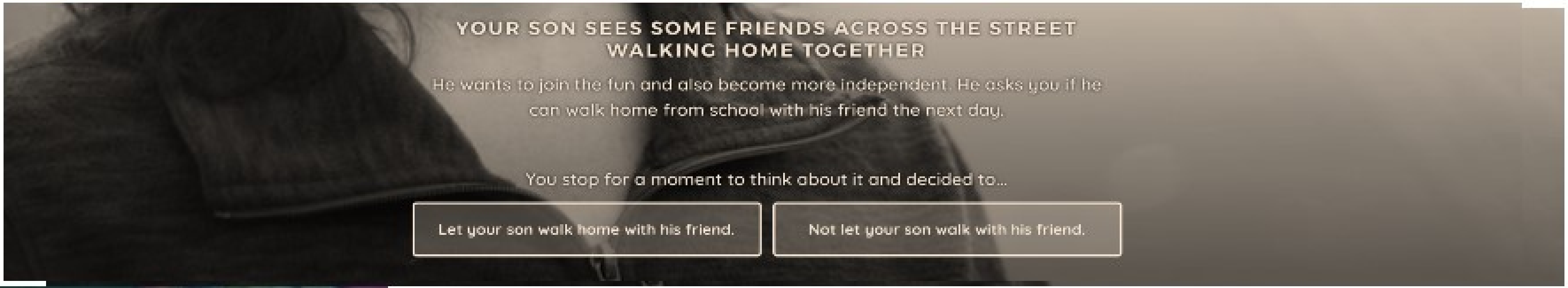

BC Children's Hospital UBC BC INJURY research and prevention unit SIO LAB

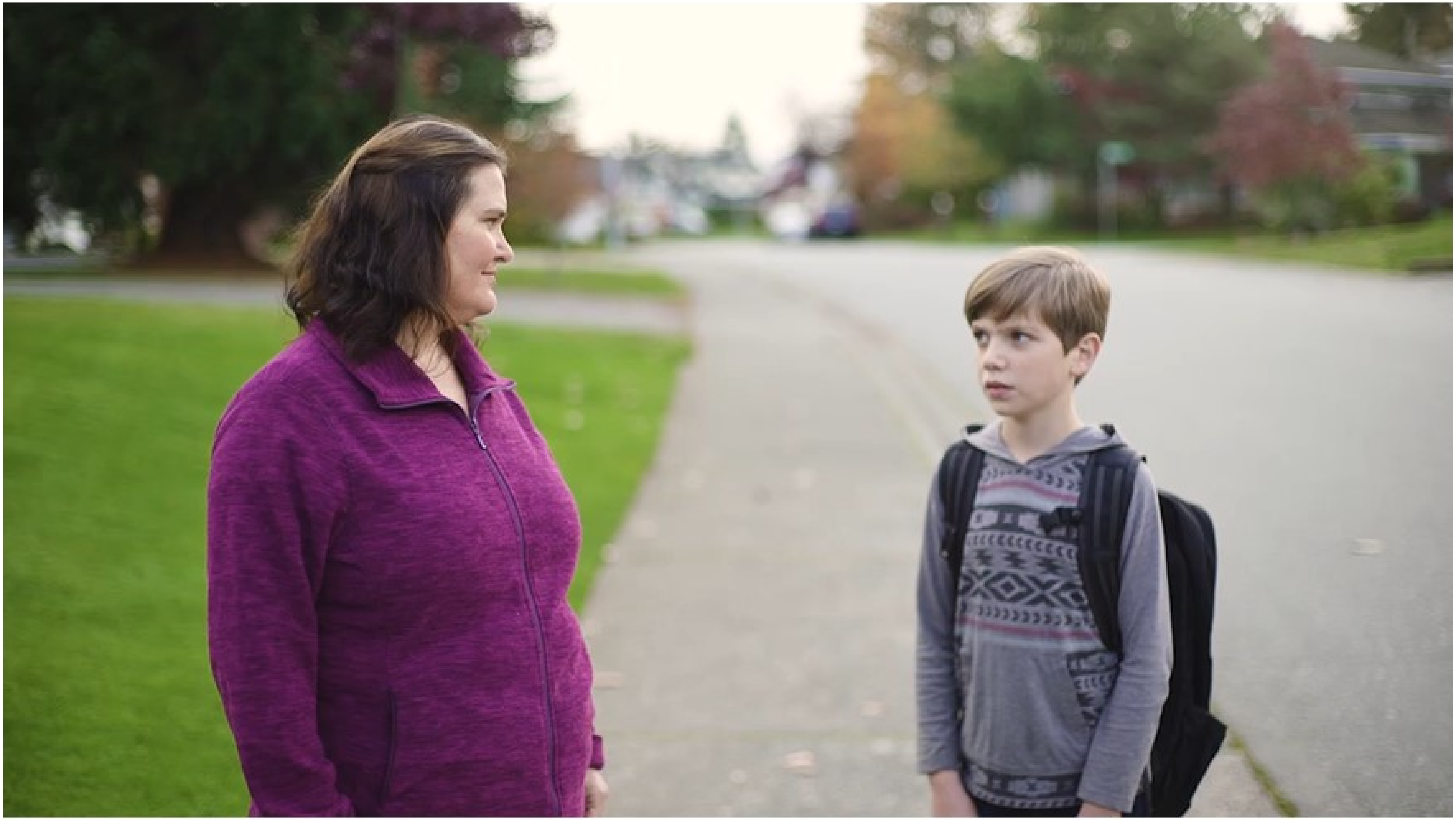

YOU CHOSE TO NOT LET YOUR SON WALK WITH HIS FRIEND

He takes one last look at his friends and with a dejected look on his face, follows behind. He feels you don't trust him and is sad at missing out.

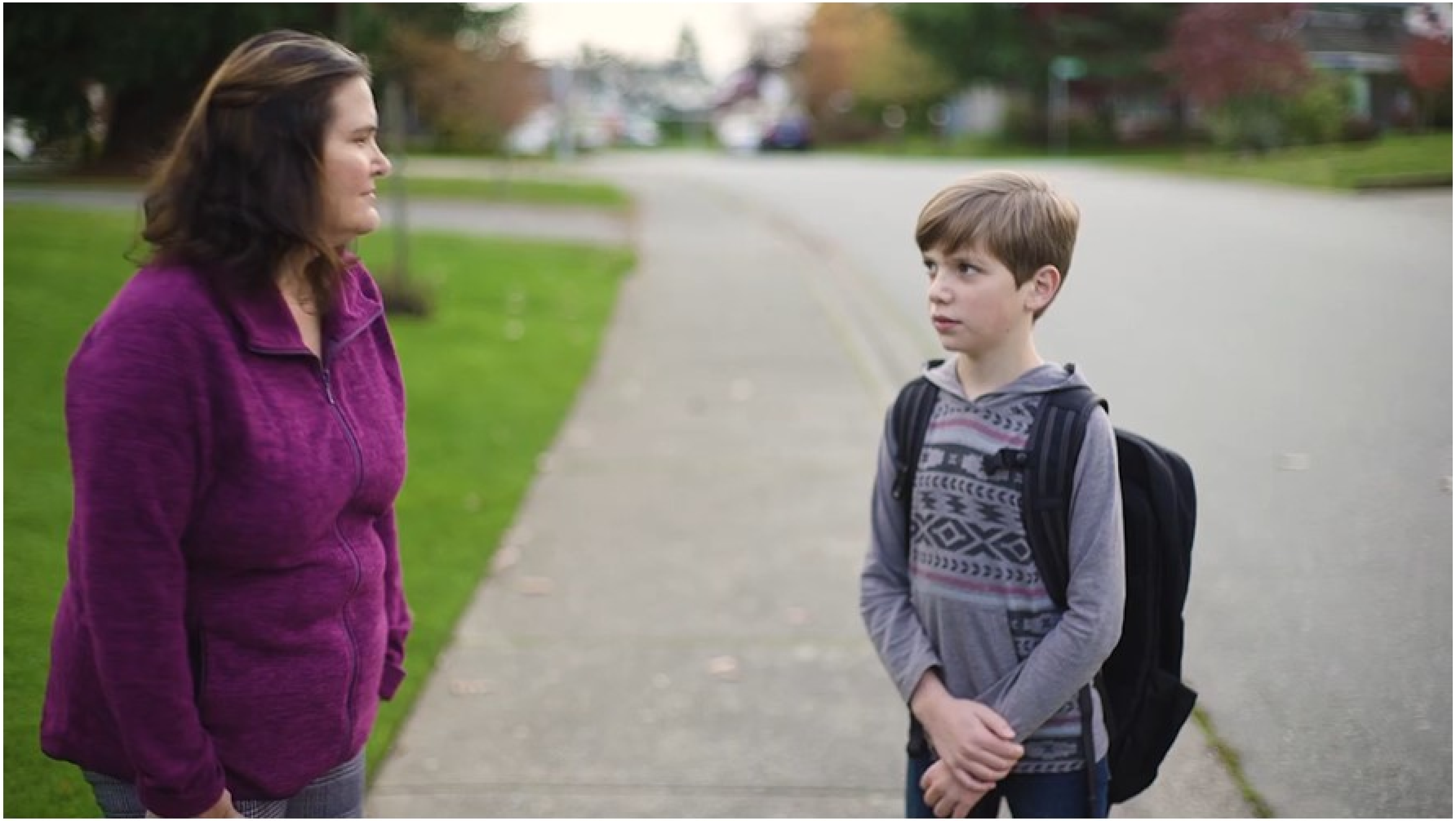

YOU CHOSE TO LET YOUR SON WALK WITH HIS FRIEND

He is excited for the opportunity to show his independence and you are proud of him for showing it.

BC Children's Hospital | UBC | BC INJURY research and prevention unit | SIO LAB

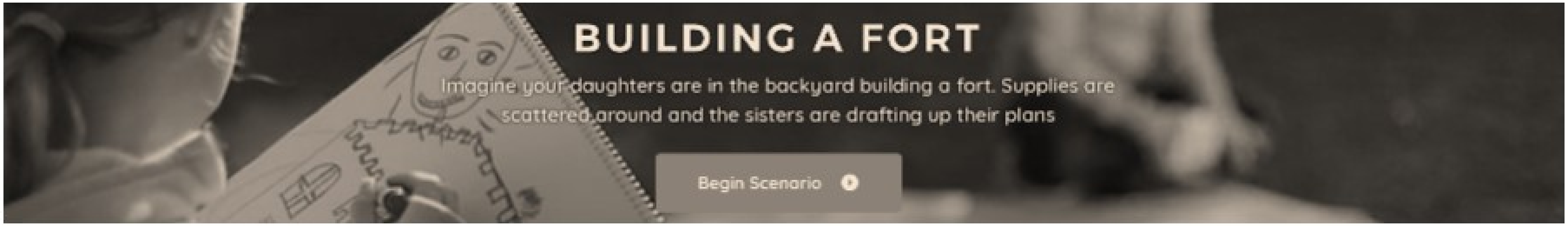
BUILDING A FORT
Imagine your daughters are in the backyard building a fort. Supplies are scattered around and the sisters are drafting up their plans
Begin Scenario

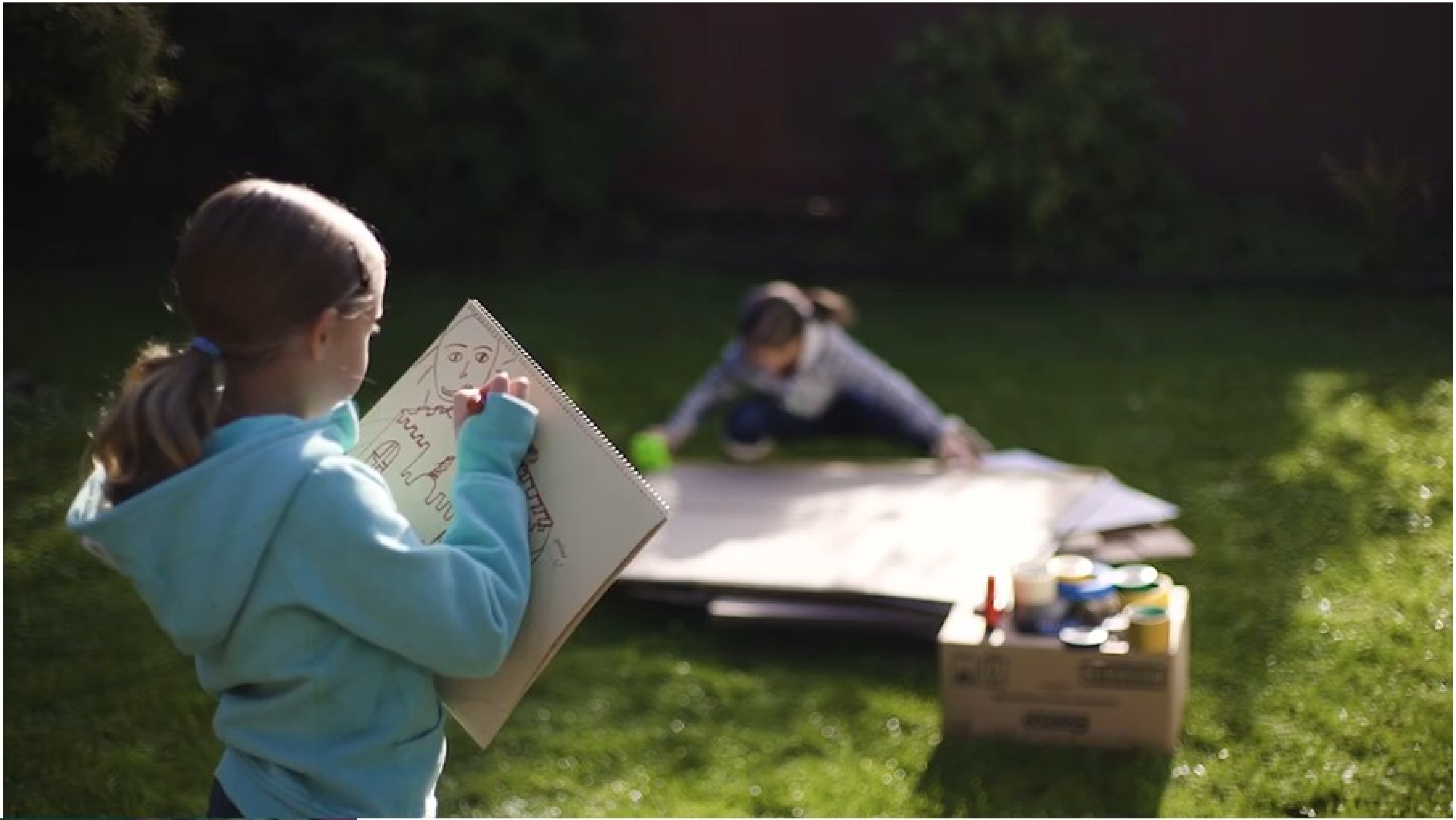

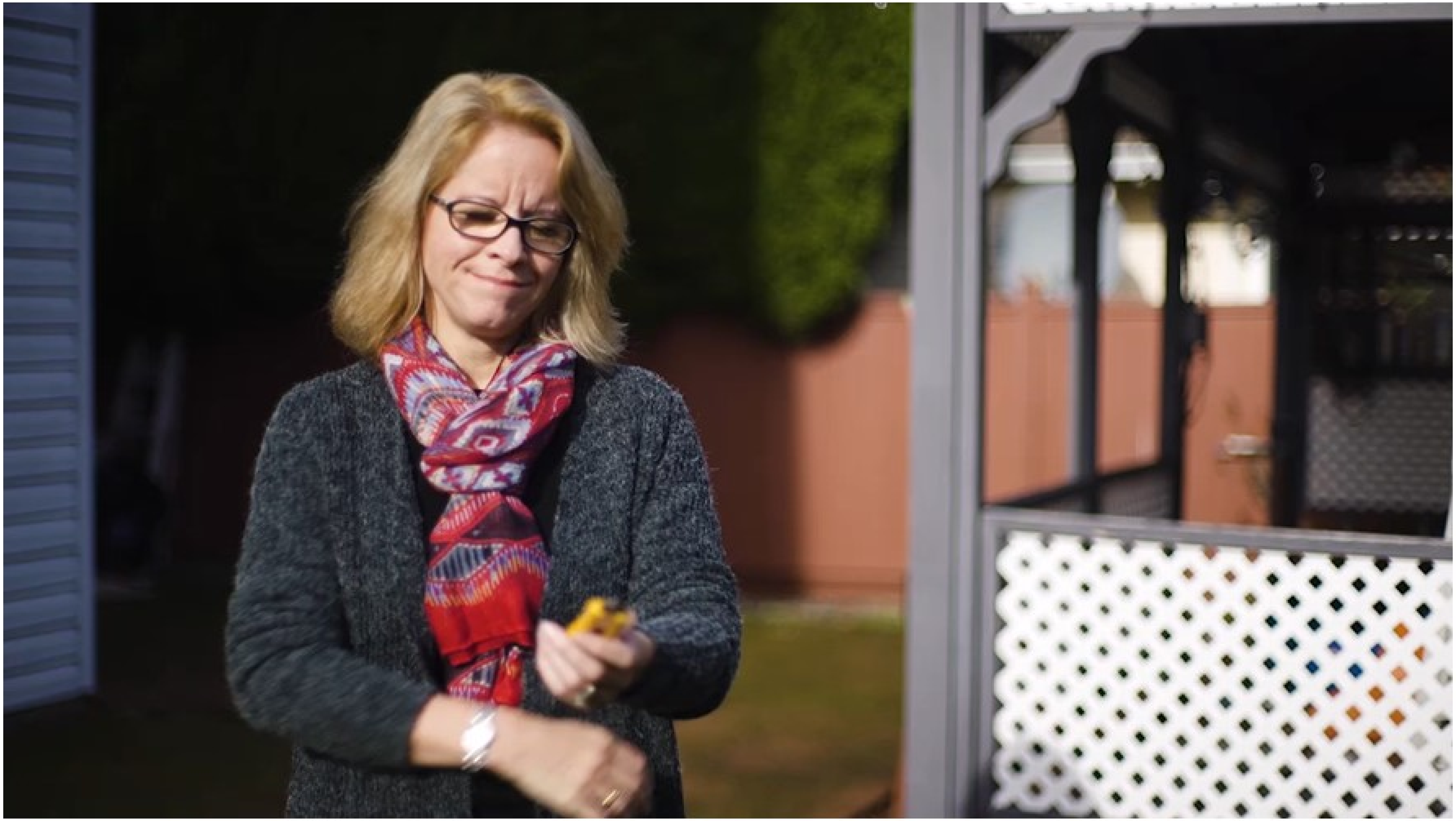

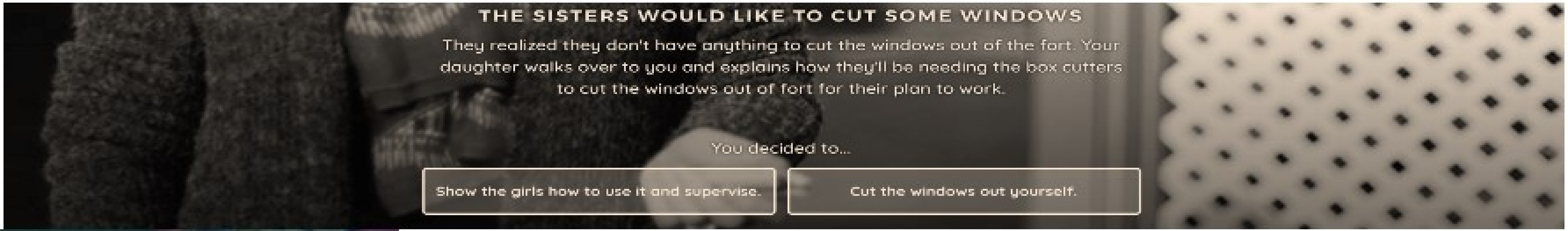

BC Children's Hospital UBC BC INJURY research and prevention unit SIO LAB

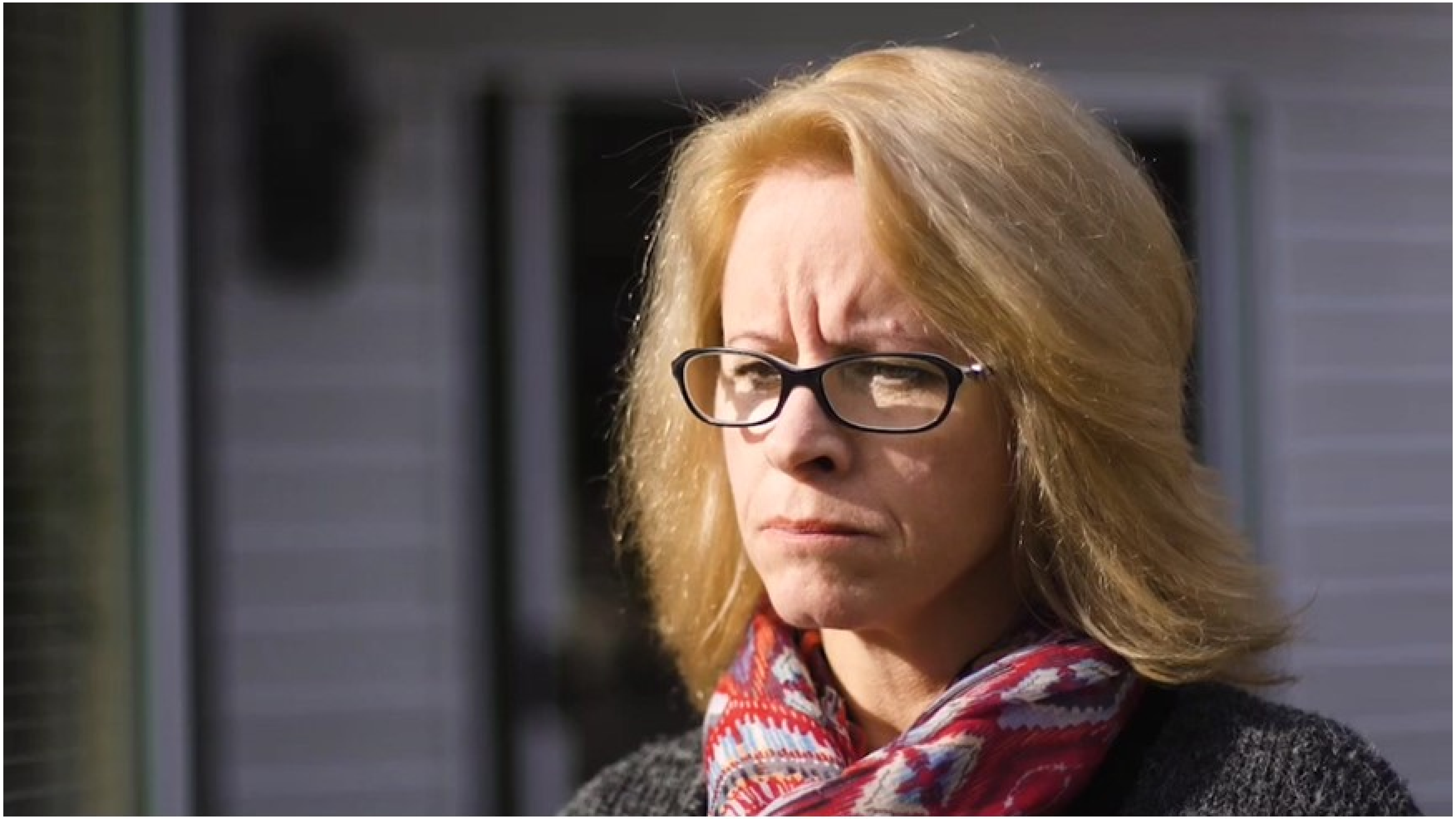

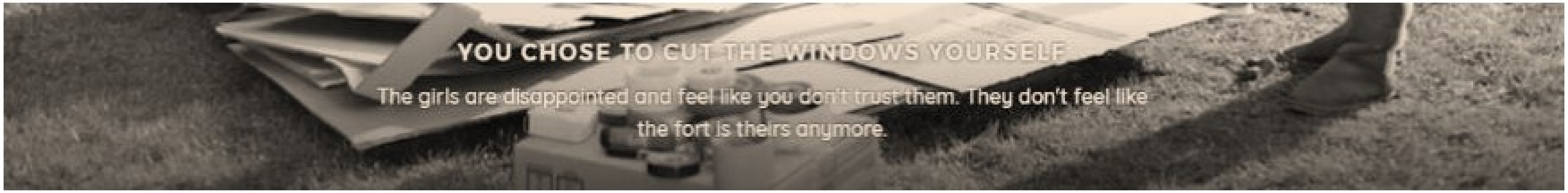
YOU CHOSE TO CUT THE WINDOWS YOURSELF
The girls are disappointed and feel like you don't trust them. They don't feel like the fort is theirs anymore.

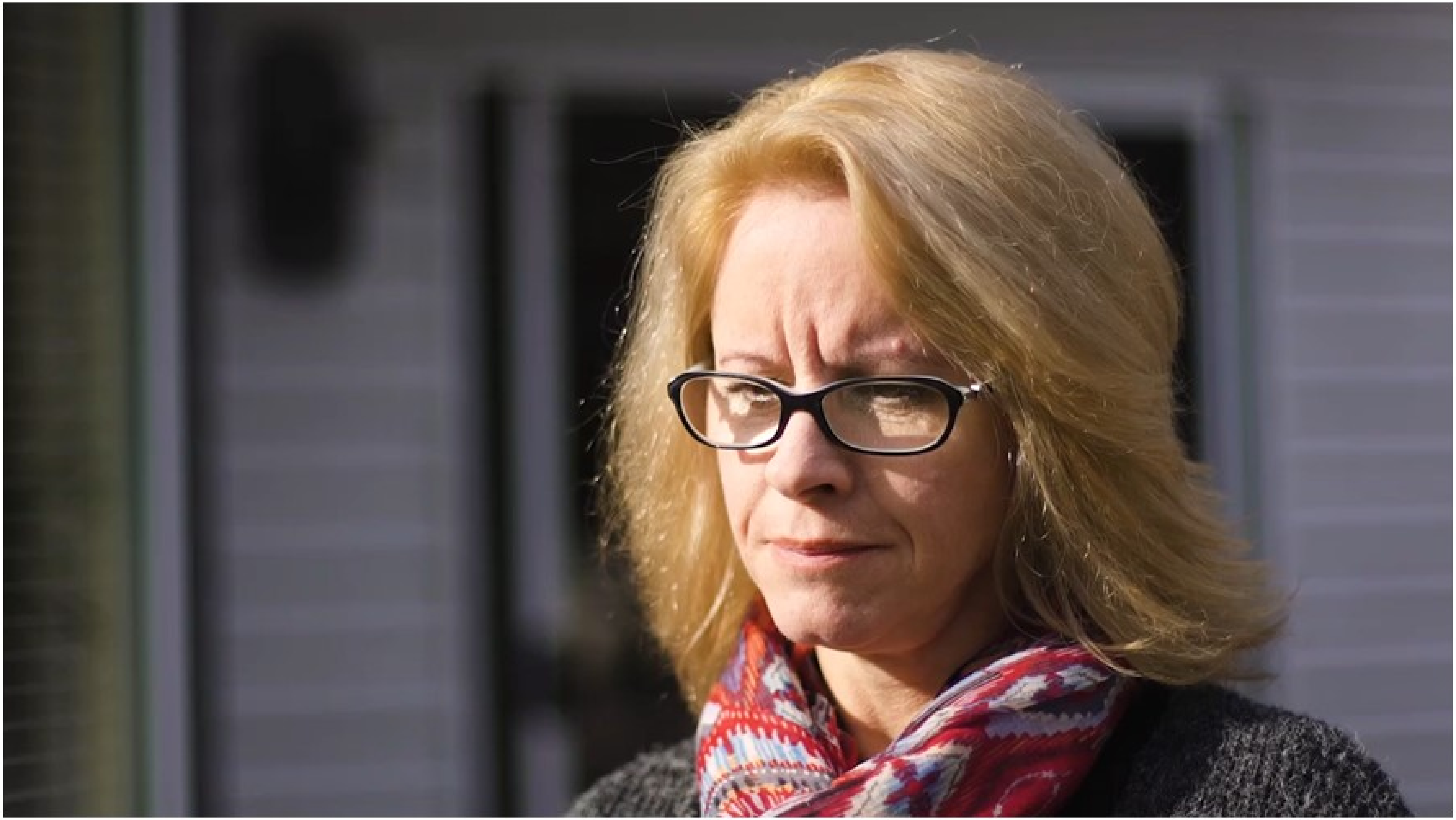

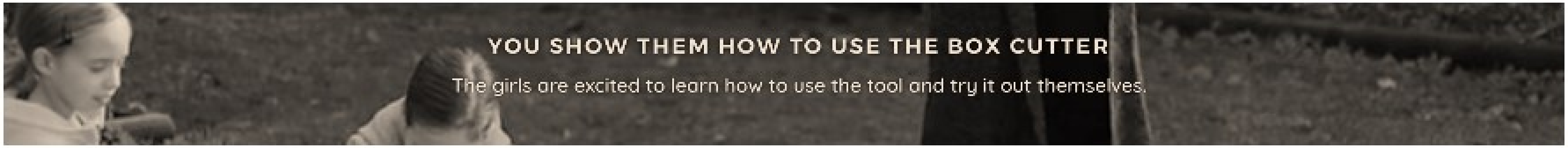

BC Children's Hospital
UBC
BC INJURY research and prevention unit
SIO LAB

Three Scenarios

(worksheet 2-1 to 2-3)

Q

2-1 Climbing a tree

- ☐ Sit back and watch **OR** ☐ Intervene

2-2 Walking home

- ☐ Let him walk home with his friends **OR** ☐ Do not let him walk with his friends

Common Concerns

(worksheet 2-4)

Q

- Remember some of your own difficult moments.
- What got in the way?

- [ ] "I am concerned my child is going to get seriously hurt."
- [ ] "I think my child does not know how to stay safe."
- [ ] "I am worried that someone is going to harm my child, either kidnapping or assault by an adult, or bullying by other children."
- [ ] "I am concerned that other people are going to think I am a bad parent for letting my child take risks."
- [ ] Write down my own answer (if any):

Things That Helped Me Let Go

(worksheet 2-5)

Q

- Think about a time when you let your child take a risk.
- What happened?

☐ “I’ve seen my child manage things like this before and trust that my child can handle it.”

☐ “I believe my child really wants to do this and I want to give my child my support.”

☐ “It is important to me that my child has opportunities to learn, build skills and try new challenges.”

☐ “I know my child really wants to do this and I think it is a good learning opportunity.”

☐ Write down your own answer (if any):

What I want: Revisited

Q

3-1

What I want: Revisited

As I revisit what I want most for my child when they grow up, what I am doing to promote those things?

Is there anything I want to change?

(worksheet 3-2)

Q

- What one thing would you like to do differently?

- How will you do it?

Letting my child climb up a tree

Next time you're at the park, watch your child for at least a few minutes without interfering at all. Get a sense of what they can already do and see how they figure out what they want to do and how they manage challenges, including climbing. Gradually extend the time you watch your child without interfering.

Letting my child use more tools

Let them try using a screwdriver while you supervise. Resist providing guidance unless my child is really struggling. Let my child use hammer and nails. Coach them on safe hammering techniques while they are trying it out. Let them try other tools, with the same gradual learning, such as box cutters and saws.

Letting my child play out in the yard without supervision

Let your child play outside for a few minutes while you watch from the window. Gradually extend this time. Then try not watching out the window.

Letting my child walk to school

Make sure to spend lots of time walking with your child to school and other destinations, while you teach them about road safety and have them model safe behaviours for you. On the walk to school, let them walk far ahead or behind you while you watch how they manage traffic safety. Let them try going on their own or with an older sibling to a nearby destination that doesn't require crossing busy streets. Let them walk home from school alone.

Make time for my child to play outside every day

Schedule time for your child to play outside on their own every day; allowing them to play without constant supervision.

Encourage my child to play outside independently.

Go out with your child while they play. Gradually move farther and farther away from them until they are comfortable with you being inside watching from the window. When they are comfortable, let them know you will be inside the house whenever they need you.

Help I will need

(worksheet 3-3)

Q

Think about help and support you might need to achieve your goal.

Home Journey Map FAQ About Us

OUTSIDEPLAY.ca

Take a risk, go play outside!

An online tool to help parents and caregivers gain the confidence to allow their kids to engage in more outdoor play.

Click to watch video!

BC Children's Hospital UBC BC INJURY research and prevention unit SIO LAB

Already know what Risky Play is and want to get started?

Start Journey

BC Children's Hospital UBC BC INJURY research and prevention unit SIO LAB

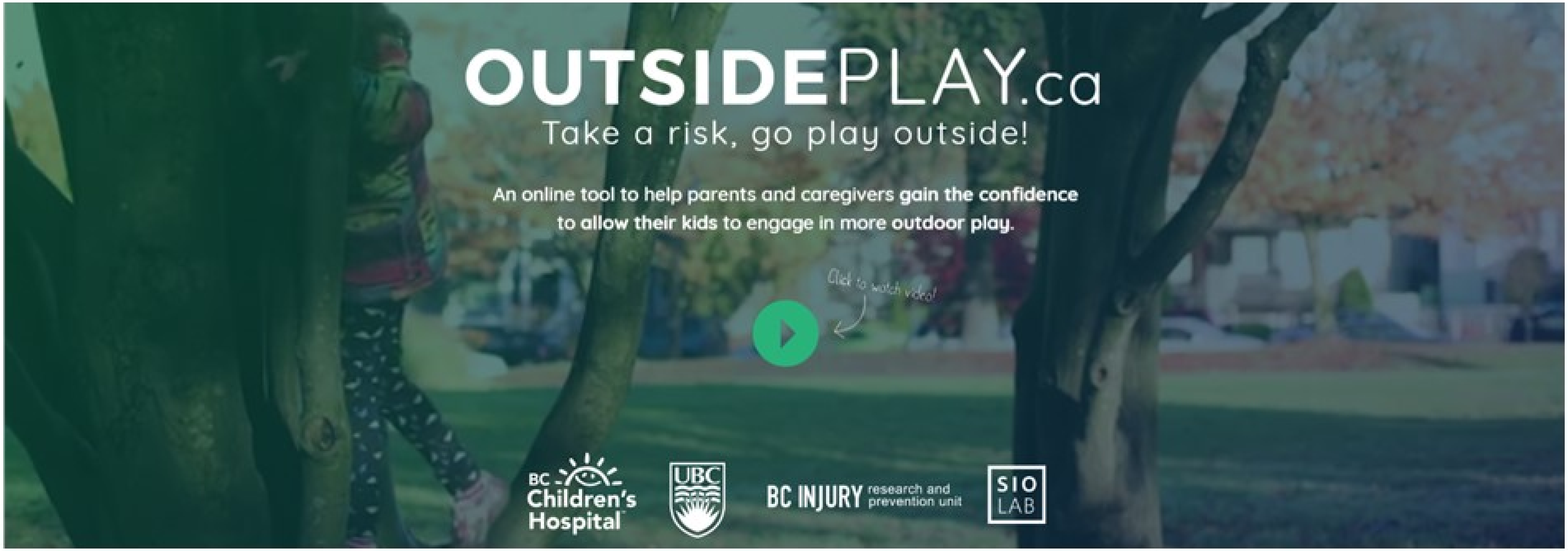

RESOURCES/FAQS

Canadian mortality rates for all unintentional injuries by sex, ages 0-19 years (1950-2009)

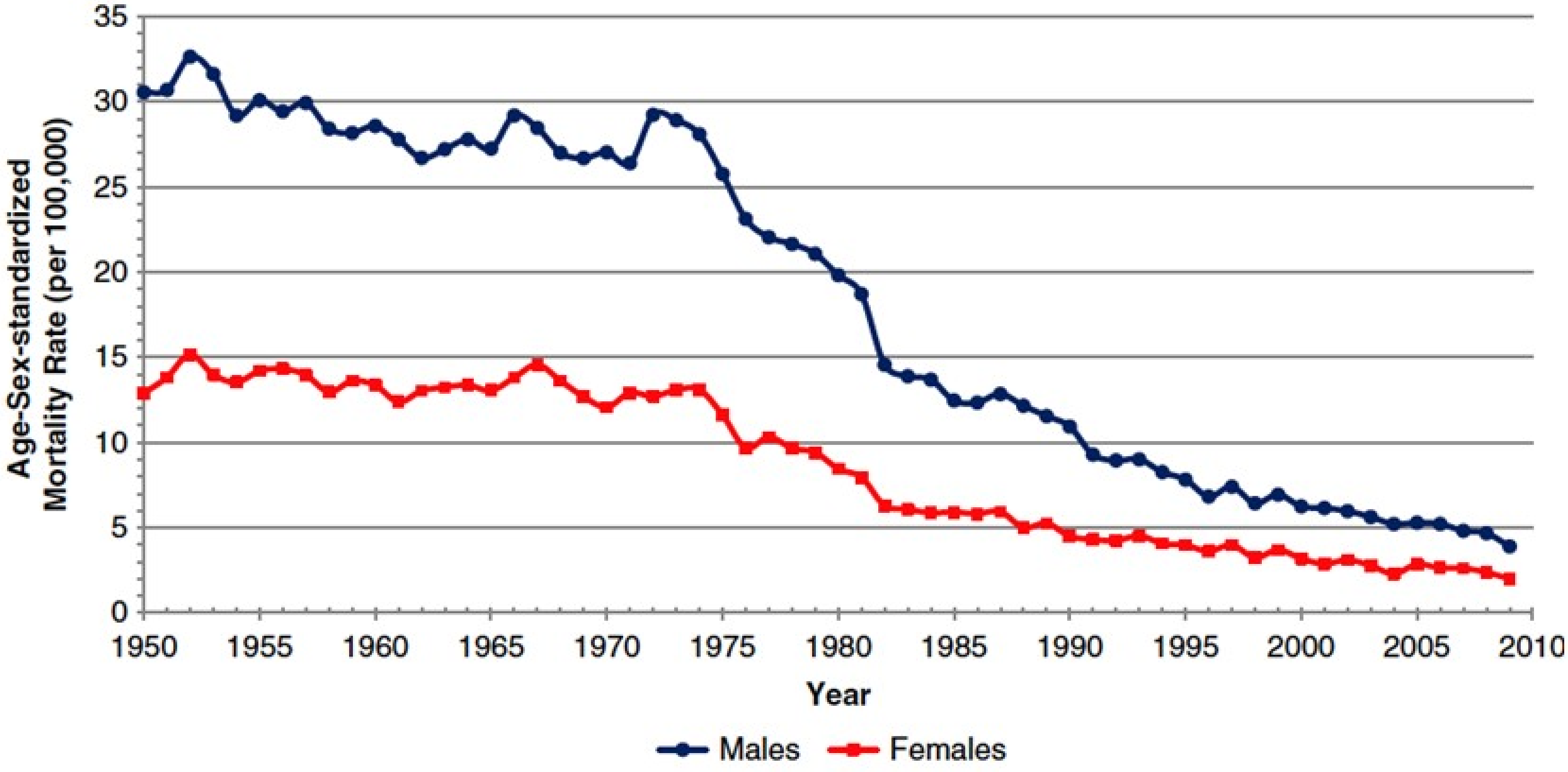

Richmond et al., 2016, CJPH, 107(4-5), e431-7

What is risky play?

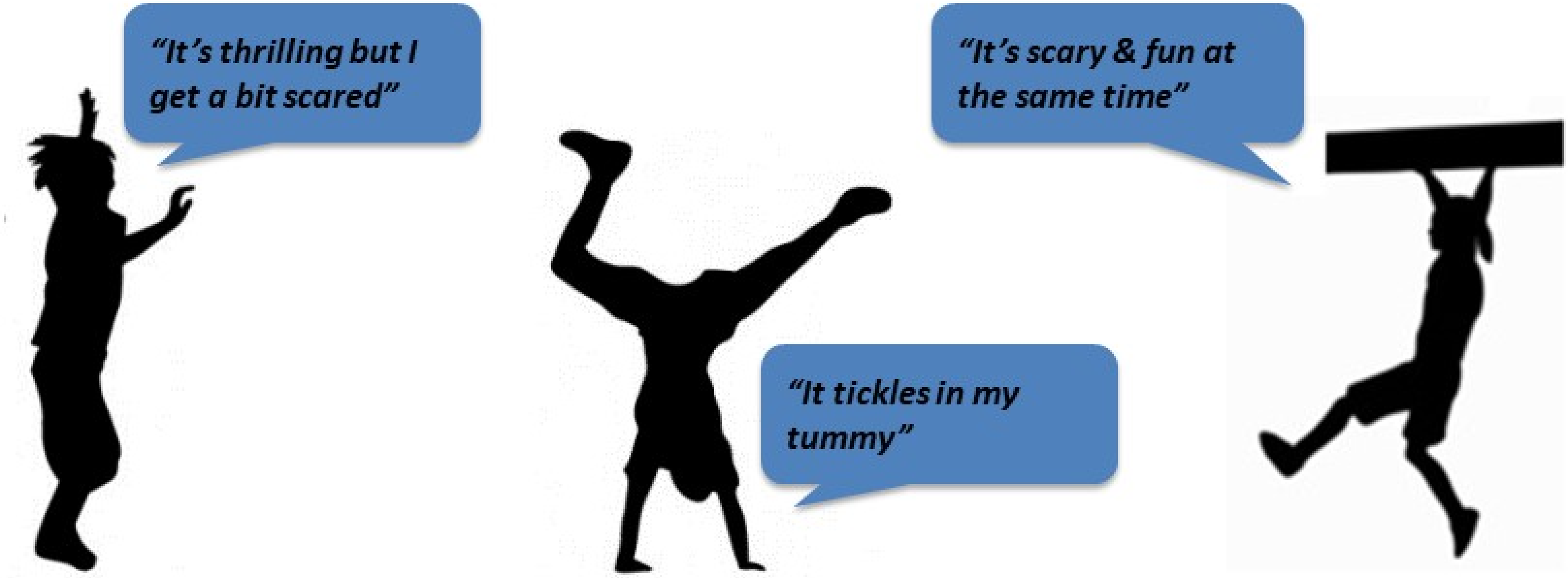

... thrilling & exciting forms of play involving uncertainty and the chance of physical injury

Risky Play

- Play with heights
- Play at high speeds
- Play with tools
- Play with elements
- Play with a chance of getting lost
- Rough and tumble play

BC Children's Hospital
UBC
BC INJURY research and prevention unit
SIO LAB

Q

Risky play sounds like what we used to call playing outside when I was a kid. Why do you need to add the word "risky"?

Q

Why is risky play important?

Q

Should I worry about my child getting seriously hurt?

Q

Should I worry about my child getting kidnapped?

Q

Isn’t my child safer inside at home?

BC Children’s Hospital
UBC
BC INJURY research and prevention unit
SIO LAB

Q

How can I get started if I'm feeling really nervous and afraid of letting my child take risks?

Q

What do I do if someone criticizes me for letting my child do something risky?

Q

Can Child Services investigate me for letting my child do something risky?

Resources

- Position Statement on Active Outdoor Play https://www.participaction.com/en-ca/thought-leadership/research/2015-position-statement-on-active-outdoor-play
- ParticipACTION Report Card on Children and Youth https://www.participaction.com/sites/default/files/downloads/Participaction-2015ReportCard-HighlightReport_1.pdf
- Free Range Kids http://www.freerangekids.com/
- Parachute Injury Prevention http://www.parachutecanada.org/
- Nature and why it's essential for kids' brains http://www.ementalhealth.ca/index.php?m=article&ID=52861

References

1. Sandseter EBH. Categorising risky play - How can we identify risk-taking in children's play? Eur Early Child Educ Res J 2007;15:237–52.
2. Shaw B, Bicket M, Elliott B, Fagan-Watson B, Mocca E, Hillman M. Children's independent mobility: An international comparison. London, England: 2015.
3. Karsten L. It all used to be better? Different generations on continuity and change in urban children's daily use of space. Child Geogr 2005;3:275–90.
4. Tandy CA. Children's diminishing play spaces: A study of inter-generational change in children's use of their neighbourhoods. Aust Geograpical Stud 1999;37:154–64.
5. Inter IKEA Systems. The Play Report 2015. Leiden: 2015.
6. Holt NL, Neely KC, Spence JC, Carson V, Pynn SR, Boyd KA, et al. An intergenerational study of perceptions of changes in active free play among families from rural areas of Western Canada. BMC Public Health 2016;16:829.
7. Brussoni M, Gibbons R, Gray C, Ishikawa T, Sandseter EBH, Bienenstock A, et al. What is the relationship between risky outdoor play and health in children? A systematic review. Int J Environ Res Public Health 2015;12:6423–54.
8. Sandseter EBH, Kennair LEO. Children's risky play from an evolutionary perspective: The anti-phobic effects of thrilling experiences. Evol Psychol 2011;9:257–84.
9. Burdette HL, Whitaker RC. Resurrecting free play in young children: looking beyond fitness and fatness to attention, affiliation, and affect. JAMA Pediatr 2005;159:46–50.
10. Nauta J, Martin-Diener E, Martin BW, van Mechelen W, Verhagen E. Injury risk during different physical activity behaviours in children: A systematic review with bias assessment. Sport Med 2015;45:327–36.
11. BCIRPU. Injury data online tool [Internet]. 2011. Available from: http://data.injuryresearch.bc.ca/datatools/DataAnalysis.aspx
12. Public Health Agency of Canada. Injury in review, 2012 edition: Spotlight on road and transport safety. Ottawa, ON: 2012.
13. Rothman L, Macarthur C, To T, Buliung R, Howard A. Motor vehicle-pedestrian collisions and walking to school: The role of the built environment. Pediatrics 2014;133:776–84.
14. Dimaggio C, Li G. Effectiveness of a Safe Routes to School Program in preventing school-aged pedestrian injury. Pediatrics 2013;131:290–6.

References (con't)

15. Lavrysen A, Bertrands E, Leyssen L, Smets L, Vanderspikken A, De Graef P. Risky-play at school. Facilitating risk perception and competence in young children. Eur Early Child Educ Res J 2015;25:89–105.
16. Ungar M. Helping parents provide children the right amount of risk and responsibility. Am J Fam Ther 2009;37:258–71.
17. Bundy AC, Luckett T, Tranter PJ, Naughton G a., Wyver SR, Ragen J, et al. The risk is that there is "no risk": a simple, innovative intervention to increase children's activity levels. Int J Early Years Educ 2009;17:33–45.
18. Schoeppe S, Duncan MJ, Badland H, Oliver M, Curtis C. Associations of children's independent mobility and active travel with physical activity, sedentary behaviour and weight status: A systematic review. J Sci Med Sport 2013;16:312–9.
19. ParticipACTION Canada. The Biggest Risk is Keeping Kids Indoors: ParticipACTION Report Card on Physical Activity for Children and Youth. Toronto, Ontario: 2015.
20. Cleland V, Crawford D, Baur LA, Hume C, Timperio A, Salmon J. A prospective examination of children's time spent outdoors, objectively measured physical activity and overweight. Int J Obes 2008;32:1685–93.
21. Dalley ML, Rucoe J. The abduction of children by strangers in Canada: Nature and scope. 2003.
22. LeMoyne T, Buchanan T. Does "hovering" matter? Helicopter parenting and its effect on well-being. Sociol Spectr 2011;31:399–418.
23. Rapee RM, Schniering CA, Hudson JL. Anxiety disorders during childhood and adolescence: Origins and treatment. Annu Rev Clin Psychol 2009;5:311–41.
24. Odenweller KG, Booth-Butterfield M, Weber K. Investigating helicopter parenting, family environments, and relational outcomes for Millennials. Commun Stud 2014;65:407–25.
25. Schiffrin HH, Liss M, Miles-McLean H, Geary KA, Erchull MJ, Tashner T. Helping or hovering? The effects of helicopter parenting on college students' well-being. J Child Fam Stud 2013;23:548–57.
26. Wyver S, Tranter P, Naughton G, Little H, Sandseter EBH, Bundy A. Ten ways to restrict children's freedom to play: The problem of surplus safety. Contemp Issues Early Child 2009;11:263–77.
27. Tremblay MS, Gray C, Babcock S, Barnes J, Bradstreet CC, Carr D, et al. Position Statement on Active Outdoor Play. Int J Environ Res Public Health 2015;12:6475–505.

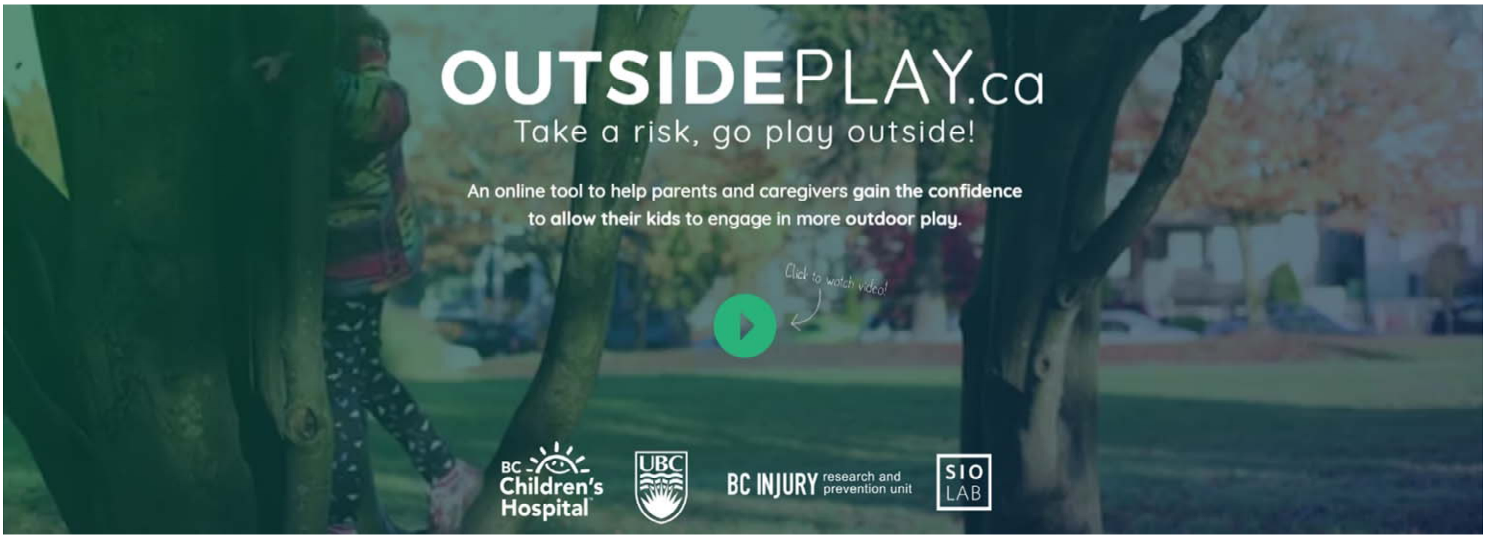

1-1

Introduction

Think about your child (between the age of 6-12). This is the child you'll be thinking about during your journey, and could be your son or daughter.

Child's first name/nickname: ________________________________.

Child's age: __________.

Child's gender: ____________.

1-2

Values & Traits

The most important attributes I want my child to have when they grow up are:

1 ____

2 ____

3 ____

1-3

My child's activities

What are some of my child's favorite things to do on their own or with friends?

1-4

Think back to my own childhood play

What were some of my favorite things to do on my own or with friends growing up? Where was I? Who was typically with me? What was I doing?

1-5

What I got out of it?

Thinking back to what I learned from these experiences and how they influenced me, what did I get out of these play activities?

1-6

Same or different?

How do my child's activities compare to what I remember doing at that age?

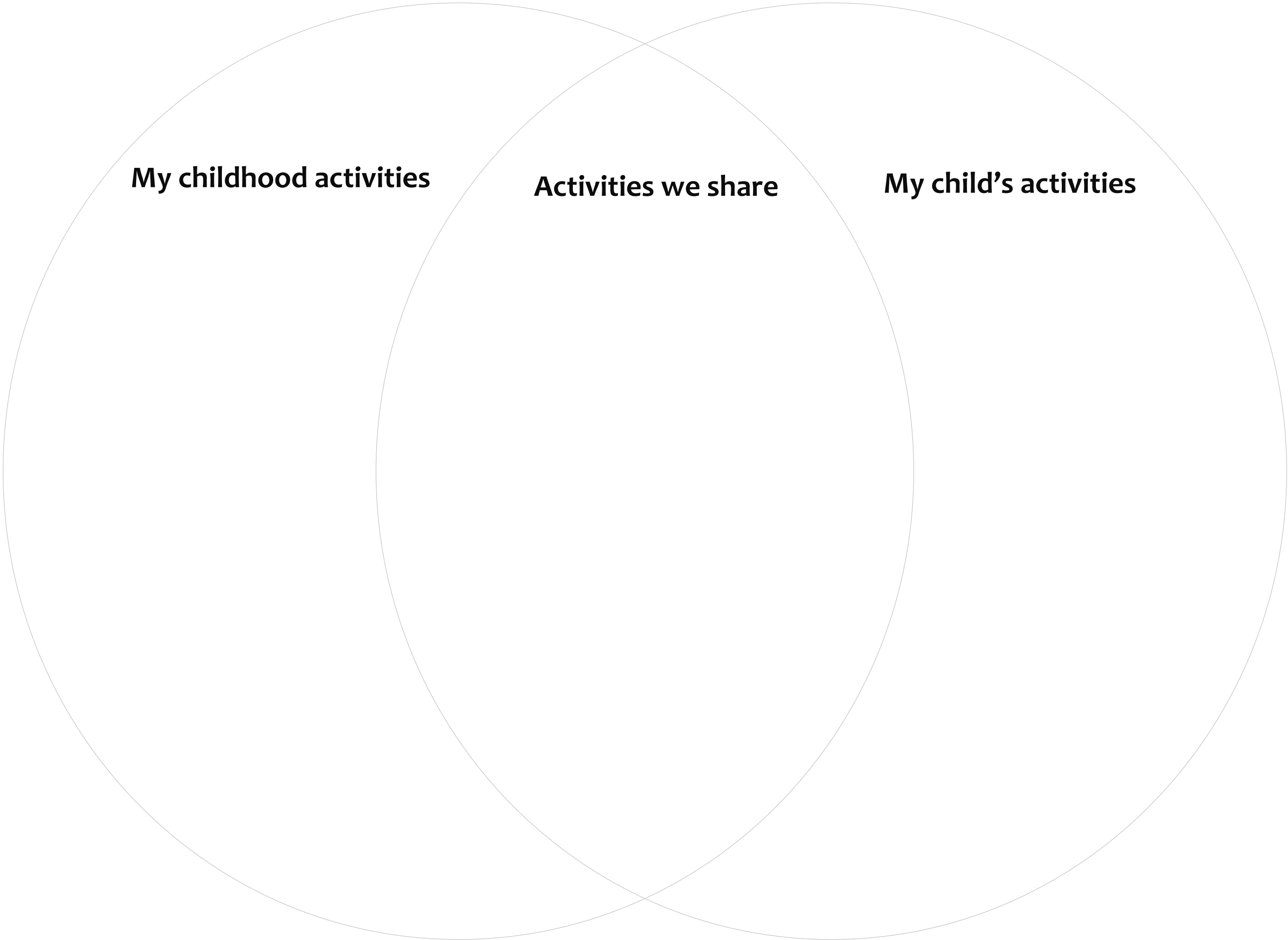

2-1 Climbing a tree

☐ Sit back and watch **OR** Intervene ☐

⇩

2-2 Walking home

☐ Let him walk home with his friends **OR** Do not let him walk with his friends ☐

⇩

2-3 Building a fort

☐ Show them how to use the box cutter and supervise **OR** Cut the windows out myself ☐

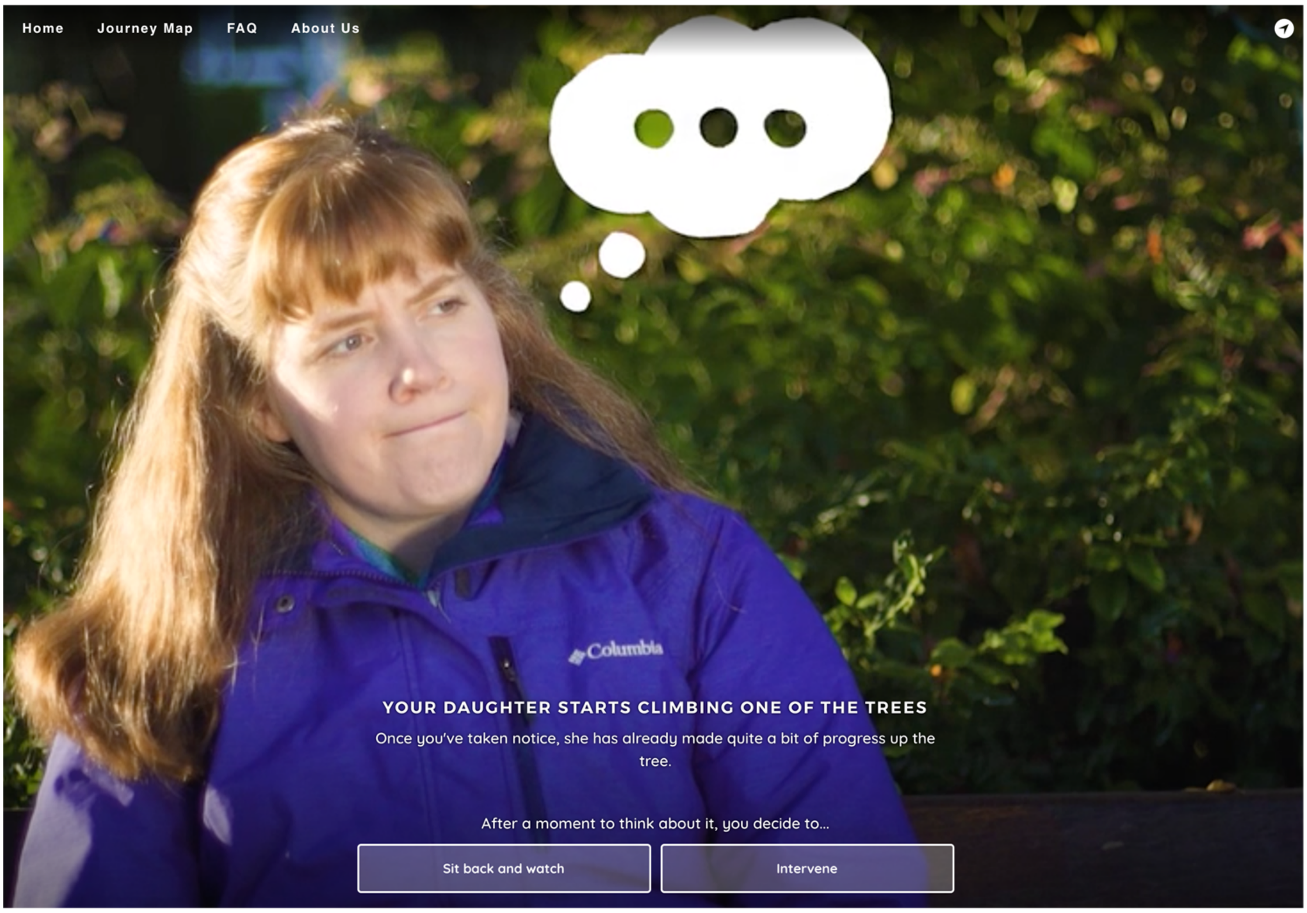

2-4

Common concerns

As parents, our fears can sometimes get in the way of our decisions about the things we let our kids do. In situations like the ones in these scenarios, what are some of the fears that have gotten in my way before? (Select all that apply).

- [ ] "I am concerned my child is going to get seriously hurt."
- [ ] "I think my child does not know how to stay safe."
- [ ] "I am worried that someone is going to harm my child, either kidnapping or assault by an adult, or bullying by other children."
- [ ] "I am concerned that other people are going to think I am a bad parent for letting my child take risks."
- [ ] Write down my own answer (if any):

2-5

Things that helped me let go

In situations like the ones in these scenarios, what are some of the things that helped me let my child keep going? (Select all that apply).

- [ ] "I've seen my child manage things like this before and trust that my child can handle it."
- [ ] "I believe my child really wants to do this and I want to give my child my support."
- [ ] "It is important to me that my child has opportunities to learn, build skills and try new challenges."
- [ ] "I know my child really wants to do this and I think it is a good learning opportunity."
- [ ] Write down my own answer (if any):

3-1 **What I want: Revisited**

As I revisit what I want most for my child when they grow up, what I am doing to promote those things?

3-2 **Is there anything I want to change?**

Goal: ______________________________

Timeline to start: ____________

Steps I will take to achieve this goal.

1 ______________________________

2 ______________________________

3 ______________________________

4 ______________________________

5 ______________________________

3-3 **Help I will need:**

Go Play Outside!

In-Workshop Facilitator Note

Revised on 20171206MB

- **What is OutsidePlay.ca?**

The University of British Columbia, the BC Children's Hospital and the BC Injury Research and Prevention Unit created OutsidePlay.ca to help parents and communities reverse the trend that is limiting children's chances to play outside and take risks in play. OutsidePlay.ca is designed to help users find a more balanced approach for them and their child through understanding the importance of risky play, working to manage the fear that can lead to setting too many limits, and building a plan of action for change.

- **About OutsidePlay.ca workshop…**

The OutsidePlay.ca workshop is for anyone or any group – especially parents and educators – who want to learn the importance of outdoor risky play for children and develop strategies for letting them engage in healthy outdoor play. The primary goal of the OutsidePlay.ca workshop is to help parents and caregivers gain the confidence to allow their kids to engage in more outdoor risky play. Gather up a small group of people interested to learn about children's outdoor risky play and take a journey together to let kids go play outside!

- **What to expect as a workshop facilitator…**

As a workshop facilitator, your main role is to guide your group through the 2-hour OutsidePlay.ca workshop while moderating face-to-face activities and discussions. Your previous knowledge around children's outdoor play would be a great asset but not necessary. This guide should include all the information you may need to host and facilitate an in-person workshop by giving you a step-by-step instruction and providing essential resources you can review beforehand. However, please remember that nothing is set in stone! Every group has its own culture and personalities – and you need to embrace each group's uniqueness and carefully adapt the tool so your workshop accommodate the specific needs and concerns of your group. Perhaps, this is the biggest and most challenging yet exciting part of your role as a OutsidePlay.ca workshop facilitator.

- **Read OutsidePlay.ca workshop resources and FAQs available at:**
    - https://outsideplay.ca/#/faq
    - In this manual page. 33-46.

© 2017 Mariana Brussoni

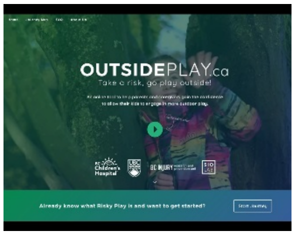

[Slide 1]

- **In preparation…**

Below is a list of the things you will need to supply or have access to in order to facilitate the OutsidePlay.ca workshop:

- ❑ A minimum of 10 participants and maximum of 15
- ❑ Space to accommodate 10-15 people (space for small discussion groups if it's a larger group)
- ❑ Tables and chairs
- ❑ Computer/laptop and display screen/projector to play the OutsidePlay.ca videos
- ❑ Audio to play the OutsidePlay.ca videos [optional]
- ❑ Print materials and some blank paper for participants
- ❑ Pens and makers for participants
- ❑ Flipcharts and markers [optional]
- ❑ Internet connection [optional]

IMPORTANT! OutsidePlay.ca workshop includes a series of video clips to be shown during the workshop. There are two ways of getting the video clips:

1. Download video clips <u>PRIOR</u> to the workshop so you can play them on your computer/laptop. You can download them from https://drive.google.com/open?id=0B_BbuXWioCo6Uk9fWVZGb0JlTDQ [You will find 4 video clips]
2. Download the full version of OutsidePlay workshop PPT with embedded video clips that you can play right off the PPT slides. [130,960KB]

- **On the day of the workshop…**
- Have print materials ready for participants.
- Have this PPT slide on the screen.

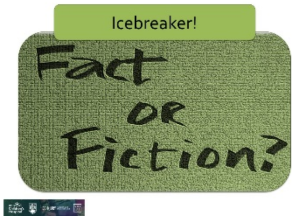

[Slide 2: ~5 min]

- **Guide 'Icebreaker!' activity**
  - Fact or Fiction game: Ask everyone to say two things about their child. One is true and one is not. Your group will guess what is true and what is not.
- **Potential questions to ask (if appropriate to your group)**
  - What is your role with regard to children and children's play?
  - Why did you attend today's workshop?

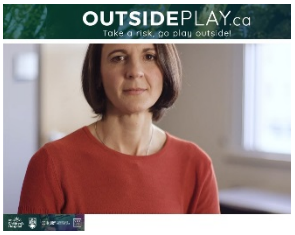

[Slide 3: ~3 min]

- **Play embedded 'Take a risk: Go Play Outside!' video.**

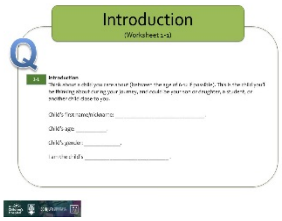

[Slide: 4: ~2 min]

- **Guide 'Introduction' activity** (i.e., Q1-1 on the worksheet).
    - This activity is to be done individually. No group discussion necessary.

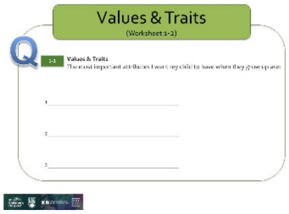

[Slide 5: ~3 min]

- **Guide 'Values & Traits' activity** (i.e., Q1-2 on the worksheet).
    - Ask participants the most important attributes (up to 3) they want their child to have when they grow up.
    - This activity could be done individually or as a group discussion by having volunteers to name 1-2 things from their list.
    - Ask participants to write down the most important attribute on the green Post-It.
- **For example**:
    - Assertive
    - Compassionate
    - Confident
    - Courageous
    - Curious
    - Independent
    - Joyful
    - Kind
    - Learns from mistakes
    - Makes good decisions
    - Passionate
    - Powerful
    - Recognition
    - Resilient
    - Sense of belonging
    - Self-content
    - Socially just
    - Spirituality
    - Imaginative
    - Wealth
    - Wisdom

© 2017 Mariana Brussoni

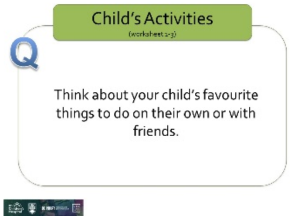

[Slide 6: ~3 min]

- **Guide 'Child's Activities' activity** (i.e., Q1-3 on the worksheet).
    - Ask participants what are some of their child's favorite things to do on their own or with friends.
    - This activity could be done individually or as a group discussion by having volunteers to name 1-2 things from their list.
    - Ask participants to write down the most important attribute on the pink Post-It.

- **See Slide 7 for examples if group struggles.**

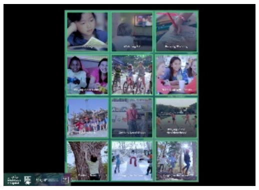

[Slide 7]

- **Some possible examples.**

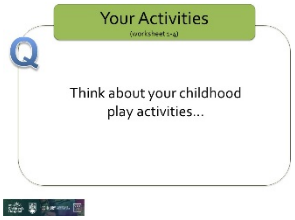

[Slide 8: ~5 min]

- **Facilitated visualization:** Encourage participants to close their eyes and really delve into the memory. Ask them to remember the sights, sounds and smells so they really immerse themselves in the memory. Ask them to think what was their favorite thing to do, something that they couldn't wait to get to. Your role as a facilitator is to help participants mentally go back to their childhood and visualize where they were, who they were with, how they felt when they were playing. [Note: These are rhetorical guided visualization questions to direct them to think about their childhood play].
- **Guide 'Your Activities' activity** (i.e., Q1-4 on the worksheet).
    - This activity is be done individually. No discussion needed.
    - Ask participants to write down the most important attribute on the blue Post-It.

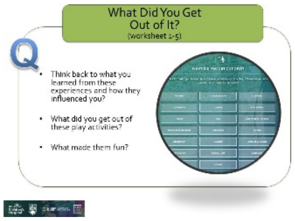

[Slide 9: ~5 min]

- **Guide 'What Did You Get Out of It' activity and discussion** (i.e., Q1-5 on the worksheet).
    - This activity is to be done as a group discussion by asking participants to share their story and discuss some of these questions. Depending on time, invite stories from 2-3 participants.
    - Potential questions to facilitate group discussion:
        - Think back to what you learned from these experiences and how they influenced you.
        - What did you get out of these play activities?
        - What made it fun?
        - Were your parents or other adults present? How did that affected your learning/benefits?
        - In retrospect, should the adults have been there?
        - Do you think your parents were neglectful?
- **See Slide 10 for examples if group struggles.**

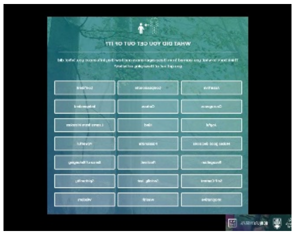

[Slide 10]

- **Some possible examples.**

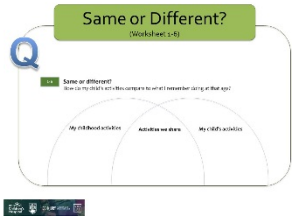

[Slide 11: ~5 min]

- **Guide 'Same or Different?' activity and discussion** (i.e., Q1-6 on the worksheet).
    - This activity is to be done as a group discussion by asking 2-3 participants to share their story and discuss some of these questions.
    - Potential questions to facilitate group discussion:
        - What are activities you share in common with your child?
        - What are activities you did but not your child?
        - What are activities that your child does that you didn't do?
    - Invite 1-2 people to share their stories; or for a larger group, small discussion groups reporting back.

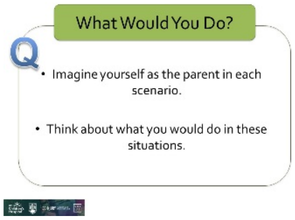

[Slide 12: ~2 min]

- **Explain 'What Would You Do' activity** (i.e., Q2-1 through Q2-3 on the worksheet).
    - Explain that they will be shown 3 scenarios and they'll imagine themselves as the parent in each scenario and think about what they would do in these situations.
    - Instruct participants to write down their choice on the worksheet Q2-1 through Q2-3.

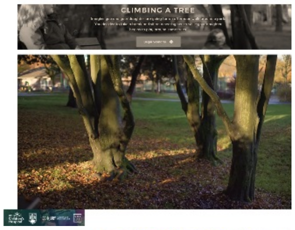

[Slide 13: ~2 min]

- **Read out** " Imagine you and your daughter are going for an afternoon walk around a park. You decided to take a break and sit on a nearby bench while your daughter begins to play around some trees."
- **Play scenario 1 'climbing a tree - intro' segment.**

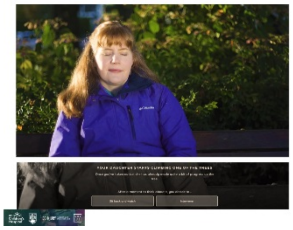

[Slide 14: ~2 min]

- **Read out** "Your daughter starts climbing one of the trees. Once you've taken notice, she has already made quite a bit of progress up the tree. After a moment to think about it, you decide to sit back and watch or intervene."
- **Play scenario 1 'climbing a tree – making the decision'.**
- **Guide 'climbing a tree' activity** (i.e., Q2-1 on the worksheet).
    - This activity is be done individually or have participants vote on which one they would choose. Regardless, show the 'intervene' clip first.

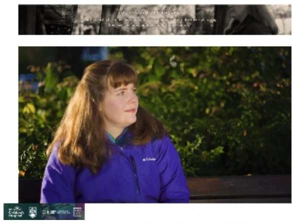

[Slide 15: ~2 min]

- **Read out** "You chose to intervene. Your first instinct is to tell your daughter to come down from the tree right away. She looks unhappy and reluctantly comes down."
- **Play scenario 1 'climbing a tree – intervene' segment.**

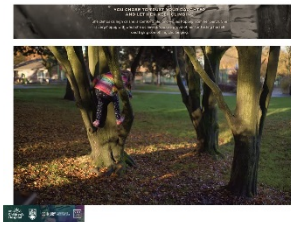

[Slide 16: ~2 min]

- **Read out** "You chose to trust your daughter and let her keep climbing. She climbs as high as she is comfortable and waves happily from her perch. She is very happy with what she achieved. You are proud of her for testing herself and trying something challenging."
- **Play scenario 1 'climbing a tree – sit-back-and-watch' segment.**

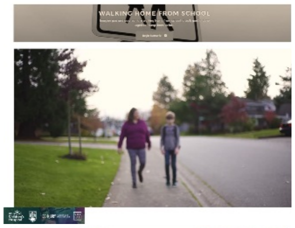

[Slide 17: ~2 min]

- **Read out** "Imagine you and your son are walking home from school, a walk you've done together many times before."
- **Play scenario 2 'walking home - intro' segment.**

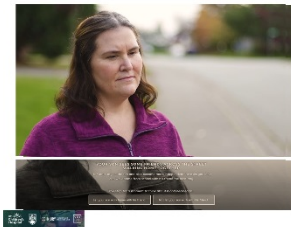

[Slide 18: ~2 min]

- **Read out** "Your son sees some friends across the street walking home together. He wants to join the fun and also become more independent. He asks you if he can walk home from school with his friend the next day. You stop for a moment to think about it and decide to let your son walk home with his friend or not let your son walk with his friend."
- **Play scenario 2 'walking home – making the decision'.**
- **Guide 'walking home' activity** (i.e., Q2-2 on the worksheet).
  - This activity is be done individually or have participants vote on which one they would choose. Regardless, show the 'not let him' clip first.

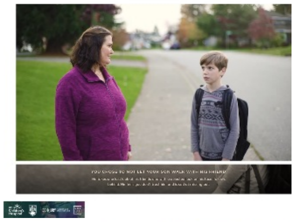

[Slide 19: ~2 min]

- **Read out** "You chose to not let your son walk with his friend. He takes one last look at his friends and with a dejected look on his face, follows behind. He feels you don't trust him and is sad at missing out."
- **Play scenario 2 'walking home – do-not-let-him' segment.**

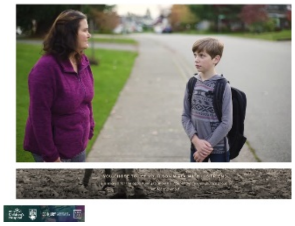

[Slide 20: ~2 min]

- **Read out** "You chose to let your son walk with his friend. He is excited for the opportunity to show his independence and you are proud of him for showing it."
- **Play scenario 2 'walking home – let-him-walk' segment.**

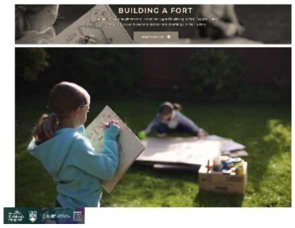

[Slide 21: ~2 min]

- **Read out** "Imagine your daughters are in the backyard building a fort. Supplies are scattered around and the sisters are drafting up their plans."
- **Play scenario 3 'building a fort – intro' segment.**

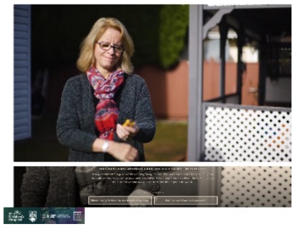

[Slide 22: ~2 min]

- **Read out** "The sisters would like to cut some windows. They realised they don't have anything to cut the windows out of the fort. Your daughter walks over to you and explains how they'll be needing the box cutters to cut the windows out of fort for their plan to work. You decided to show the girls how to use it and supervise or cut the windows out yourself."
- **Play scenario 3 'building a fort – making the decision'.**
- **Guide 'building a fort' activity** (i.e., Q2-3 on the worksheet).
  - This activity is be done individually or have participants vote on which one they would choose. Regardless, show the 'cut the windows out yourself' clip first

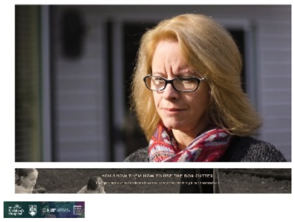

[Slide 23: ~2 min]

- **Read out** "You chose to cut the windows yourself. The girls are disappointed and feel like you don't trust them. They don't feel like the fort is theirs anymore."
- **Play scenario 3 'building a fort – cut-the-windows-out-myself' segment.**

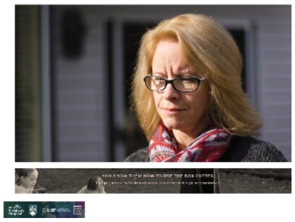

[Slide 24: ~2 min]

- **Read out** "You show them how to use the box cutter. The girls are excited to learn how to use the tool and try it out themselves."
- **Play scenario 3 'building a fort – show-them-how-to-use-the-box-cutter-and-supervise segment.**

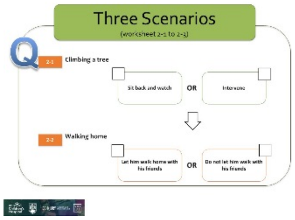

[Slide 25: ~10 min]

- **Guide discussion going over participant responses to 'Three Scenarios' activity (i.e., Q2-1 through Q2-3): Ask how many people choose to intervene or not for each scenario.**
  - **This activity is be done as a group discussion by asking 2-3 participants to share their story and discuss some of these questions.**
  - **Potential questions to facilitate group discussion:**
    - **Why did you choose to intervene? What are the main concerns that led you to intervene?**
    - **Why did you choose to let kids do what they want to do? What made you decide to let them do that they want to do?**
    - **How do you think the child perceived the situation? (Note: Make sure to explore the child's mind for both the 'intervene' and 'allow' situations.)**

- **Across the three scenarios, do you see any patterns around your concerns?**

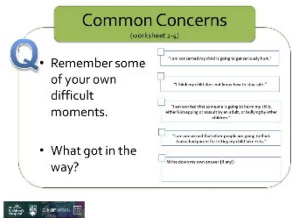

[Slide 26: ~10 min]

- **Guide 'Common Concerns?' activity and discussion** (i.e., Q2-4 on the worksheet).
  - This activity is to be done as a group discussion by asking 2-3 participants to share their story and discuss some of these questions.
  - Spend more time on this topic if time permits. This is more important than discussion for Slide 25.
  - Potential questions to facilitate group discussion:
    - As parents, our fears can sometimes get in the way of our decisions about the things we let our kids do. Remember some of your own difficult moment of letting kids do what they wanted to do. What got in the way?
    - Can you identify your main concerns and fears?

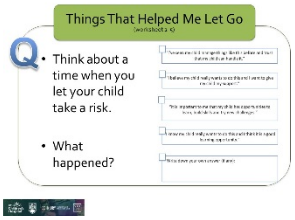

[Slide 27: ~10 min]

- **Guide 'Things That Helped Me Let Go' activity and discussion** (i.e., Q2-5 on the worksheet).
  - This activity is to be done as a group discussion by asking 2-3 participants to share their story and discuss some of these questions.
  - Encourage participants to think about a time when they let their child take a risk.
  - Spend more time on this topic if time permits. This is more important than discussion for Slide 25.
  - Potential questions to facilitate group discussion:
    - What happened?
    - What are some of the things that helped you let your child keep going?
    - How did you know your child was ready?
- If you encounter any participant who says they've never let their child take a risk, take it as a teachable opportunity and put daily activities that are risky (e.g., walking down the street, riding in a car) into perspective.

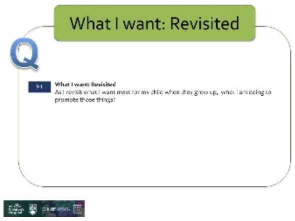

[Slide 28: ~5 min]

- **Guide 'What I want: Revisited…' activity** (i.e., Q3-1 on the worksheet)
  - Guide participants to refer back to their answers to Q1-2 on the worksheet. This acts as a prompt to remind them what they've identified as the most important for their child when they grow up.
  - Then, guide them to reflect on what they are doing to promote those things.
  - This acts as a lead-in to the discussion on the next slide where they'll be reviewing what they want to change.
  - This activity is be done individually.

© 2017 Mariana Brussoni

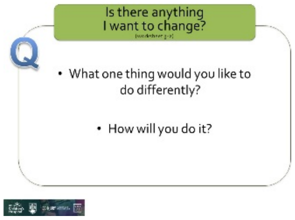

[Slide 29: ~10 min]

- **Guide 'Is there anything I want to change?' activity and discussion** (i.e., Q3-2 on the worksheet)
  - The purpose of this activity is to make a detailed and feasible action plan as way for participants to make the change. In order to do that, as a facilitator, you guide them to reflect on their responses to Q2-4 and Q2-5 on worksheet and support them to 1) come up with one broad goal they want to achieve; and 2) break this goal down to smaller/achievable steps.
  - You could ask for a volunteer to discuss with the group the change they want to make and talk through how they're going to do it. This should be a group discussion where everyone can chime in with their own thoughts and suggestions – not just putting that one person on the spot to come up with answers.
  - Here are a few questions you can guide group discussion so they come up with their own answer and plan.
    - Is there anything you want to change? What one thing would you like to do differently? How will you do it?
    - You now have the opportunity to craft an action plan based on your worksheet. Your action arising from your plan can be as large or small as you like, as long as they're realistic for you.
    - Set yourself one goal and pick some different steps that can help you overcome some of your fears.
- **Coming up with a goal can be challenging for some participants. See Slide 30 for examples if group struggles.**

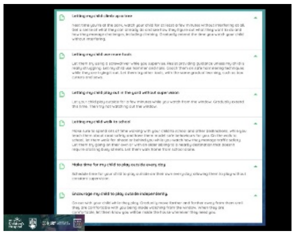

[Slide 30]

- **Some possible examples.**

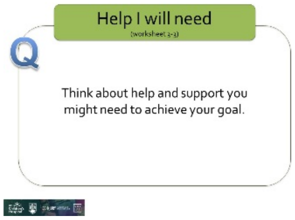

[Slide 31: ~5 min]

- **Guide 'Help I will need' activity and discussion** (i.e., Q3-3 on the worksheet)
  - Guide participants to think about help they might need to achieve their goal. For example:
    - Talk with their partner, family, friends, or neighbours about letting children engage in more outdoor risky play.
    - Encourage their partner, family friends, or neighbours to take the OutsidePlay.ca tool.
    - Find a mentor or a supportive social network that understands your goal and supports your values.

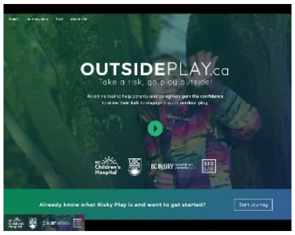

[Slide 32: 5+ min]

- **Ask if there is any questions or issues they want to discuss.**
- **Wrapping up.**
  - Let participants know that they can revisit all this material and their journey at https://outsideplay.ca and encourage them to share it with their friends and community.
- **Q&A**: See Slide 33-46 for answers to common questions and potential discussion points.

© 2017 Mariana Brussoni
