# Supplementary material for: A Web-Based and In-Person Risk Reframing Intervention to Influence Mothers’ Tolerance for, and Parenting Practices Associated With, Children’s Outdoor Risky Play: Randomized Controlled Trial
Source: J Med Internet Res. 2021 Apr 27;23(4):e24861. doi: 10.2196/24861 (PMC8114163; doi:10.2196/24861)
Supplement: Multimedia Appendix 4 [file jmir_v23i4e24861_app4.pdf]

Statistical differences for other sociodemographic characteristics

1-week after the intervention

Age: *P*=.71; ethnicity: *P*=.92; marital status: *P*=.26; education: *P*=.09; employment: *P*=.21; home dwelling: *P*=.82; income: *P*=.24; exposure to risky play information: *P*=.25; child age: *P*=.28.; child sex: *P*=.68; weekday outdoor time: *P*=.34; weekend outdoor time: *P*=.93.

3 months after the intervention

Age: *P*=.84; ethnicity: *P*=.89; marital status: *P*=.38; education: *P*=.09; employment: *P*=.41; home dwelling: *P*=.90; income: *P*=.26; exposure to risky play information: *P*=.14; child age: *P*=.28; child sex: *P*=.59; weekday outdoor time: *P*=.41; weekend outdoor time: *P*=.88.

When **comparing those who completed the intervention with those who did not**, the following were obtained: age: *P*=.11; ethnicity: *P*=.08; marital status: *P*=.98; education: *P*=.34; home dwelling: *P*=.33; income: *P*=.65; exposure to risky play information: *P*=.40; child age: *P*=.09; child sex: *P*=.97; child’s disability/chronic condition: *P*=.59; weekday outdoor time: *P*=.79; weekend outdoor time: *P*=.25
